# Encapsulation of Linear and Cyclic Alkanes in Water Within a Dimeric Deep‐Cavity Sulfonatocalix[4]arene Capsule

**DOI:** 10.1002/chem.71682

**Published:** 2026-09-11

**Authors:** Nico Schneider, Sebastiano Casalino, Aldrik H. Velders, Rosa M. Gomila, Antonio Frontera, Stefan Kubik

**Affiliations:** ^1^ Fachbereich Chemie – Organische Chemie RPTU University Kaiserslautern‐Landau Kaiserslautern Germany; ^2^ Laboratory of BioNanoTechnology Wageningen University Wageningen the Netherlands; ^3^ Departament de Química Universitat De Les Illes Balears Baleares Spain

**Keywords:** alkanes, hydrophobic effect, molecular capsule, sulfonatocalix[4]arene, water

## Abstract

The tetrasulfonated deep‐cavity calix[4]arene **1**, bearing a second row of benzene rings around its upper rim, self‐assembles into a dimeric capsule in water upon encapsulation of small hydrophobic guests. ^1^H NMR spectroscopy shows that the two calixarene subunits interdigitate, creating an elongated hydrophobic cavity that accommodates linear and cyclic alkanes, as well as benzene and hexafluorobenzene. NMR competition experiments establish a guest size‐dependent stability sequence: among the linear alkanes, complex stability peaks at *n*‐hexane and decreases for both shorter and longer homologs, whereas the cycloalkane complexes become steadily more stable from cyclopentane to cyclooctane, with the cyclooctane complex outcompeting the *n*‐octane complex. Benzene forms a comparatively stable complex that is displaced by cyclohexane and cycloheptane, while hexafluorobenzene is in turn displaced by benzene. DFT calculations (BP86‐D4(COSMO = water)/def2‐SVP) of substitution energies and packing coefficients reproduce these trends qualitatively, particularly for the larger guests, and rationalize the guest size dependence in terms of a shift from single‐ to bis‐subunit CH···π contacts, and, for hexafluorobenzene, a central binding position that minimizes F···π interactions. Together, these results establish deep‐cavity calix[4]arene **1** as a versatile, hydrophobic effect‐driven capsule for the recognition of small apolar molecules in water.

## Graphical abstract

Captured in water. A tetrasulfonated deep‐cavity calix[4]arene encapsulates linear and cyclic alkanes, as well as benzene and hexafluorobenzene, in water by forming a self‐assembled dimeric capsule. The binding affinity is guest size‐dependent. For linear alkanes, the affinity peaks at *n*‐hexane, while cyclooctane is the most strongly bound cycloalkane.

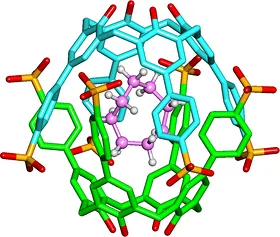

## 1 Introduction

Molecular capsules are hollow structures formed from two or more appropriately shaped building blocks by noncovalent self‐assembly [1, 2, 3]. Capsule formation creates a confined space, often shielded from the surrounding solvent, which is available for the incorporation of one or more guest molecules. More robust hollow structures, called coordination or covalent cages, can be formed through metal coordination [4] or covalent bonds [5, 6, 7], respectively. An early example of a molecular capsule is Rebek's molecular tennis ball, comprising two self‐assembled C‐shaped bis(glycoluril) moieties [8]. While the cavity of this capsule is small, only allowing the incorporation of one small molecule, such as chloroform or methane, related systems, such as the molecular softball or the molecular jelly doughnut, feature larger cavities [1]. A cavity of approximately 425 Å^3^ is produced by a homodimeric capsule formed from two deep cavitand subunits [9]. This capsule can include two aromatic guests or linear alkanes in their extended or coiled conformation, depending on the match between the length of the cavity and the alkane chain length.

Capsules can alter the reactivity of the included guest, stabilize thermodynamically unfavorable conformations and short‐lived intermediates, and mediate intra‐ or intermolecular reactions [10]. For example, the large hexameric capsules formed from resorcin[4]arene subunits have been shown by Tiefenbacher and others to mediate chemical transformations of the guest molecules that differ significantly with respect to reactivity and selectivity from the corresponding reactions outside the capsule [11, 12, 13]. Other examples of dimeric capsules include the self‐assembling calix[4]arene tetraurea developed by Rebek [14] and Böhmer [15], the self‐assembling calix[4]pyrroles described by Ballester [16] and related systems [17, 18, 19]. In addition, dimeric resorcin[4]arene‐derived capsules have been observed in the solid state, which are in some cases stabilized by the guest molecule [20, 21].

Most capsules feature hydrogen bonds between the building blocks. Such capsules are typically stable only in apolar media. Furthermore, investigations are usually performed in the presence of solvent molecules that are too large to enter the cavities. In water, hydrogen bonds are generally too weak to mediate capsule formation. However, it has been shown that it can be induced by guest binding. For example, incorporating long‐chain alkanes (C11–C14) into Rebek's deep cavitand results in an alkane‐filled, dimeric capsule that also assembles in water [22, 23]. In other capsules, halogen or chalcogen bonds contribute to the self‐assembly [24, 25].

Capsule formation entirely driven by the hydrophobic effect has been demonstrated by Gibb for the octa acid cavitand [26]. This cavitand has an extended hydrophobic surface along the cavity that is enlarged by filling the cavity with a hydrophobic guest molecule. When these complexes dimerize in water, the water molecules at the cavity opening are released, thus mediating capsule formation. Alternatively, octa acid dimers are stabilized by the binding of large guest molecules, such as longer alkanes, that span the two capsule halves. Other examples of capsules in water were reported by Shionoya [27] and Yoshizawa [28]. These capsules mediate the dissolution of guest molecules that would normally be insoluble in water. The guests are thus stabilized and protected in a manner similar to how capsid proteins encapsulate and protect the genetic information of viruses.

In the course of our work on supramolecular scavengers for nerve agents [29, 30], we prepared the tetrasulfonated 5,11,17,23‐tetraphenylcalix[4]arene **1** (Scheme 1), which we expected to bind organophosphates, such as VX or Novichoks, more strongly than a tetrasulfonatocalix[4]arene lacking the second row of benzene units. Such deep‐cavity calix[4]arenes have been described before, but their binding properties have not been investigated in detail. An analogous pentaphenylcalix[5]arene lacking the sulfonate groups has been shown to form a sandwich complex with C_60_ in the solid state [31]. Rebek and coworkers showed that a deep calix[4]arene with urea groups along the cavity opening forms a hydrogen‐bonded dimer in CD_2_Cl_2_, which can bind hydrophobic guests [32]. In our binding studies, we found that apolar guests induce **1** to form a dimeric capsule in water. NMR spectroscopy and computational studies suggested that two molecules of **1** interdigitate upon capsule formation, bringing two concave surfaces together to form a hollow structure that can host various guest molecules. Capsule formation is driven by the dehydration of the guests and parts of the capsule halves, with potential additional contributions coming from aromatic interactions between the interdigitating side arms of **1**. To investigate this capsule motif, we systematically screened a series of hydrophobic molecules as guests, aiming to establish a relationship between capsule formation and guest size. The results of these investigations are reported here.

**SCHEME 1:**
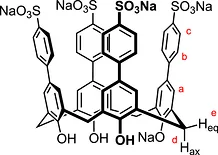
Structure of the pentasodium salt of 5,11,17,23‐(4‐sulfonatophenyl)‐25,26,27,28‐tetrahydroxycalix[4]arene **1**.

## 2 Results and Discussion

### 2.1 Synthesis and Characterization

The synthesis of **1** has been described before [33]. In the previous work, **1** was obtained by macrocyclization of 4‐phenylphenol with formaldehyde under the conditions established for the synthesis of *tert*‐butylcalix[4]arene [34], followed by sulfonation. We instead preferred an approach starting from commercial *tert*‐butylcalix[4]arene (Scheme 2). This compound was dealkylated with AlCl_3_ and then brominated to yield 5,11,17,23‐tetrabromo‐25,26,27,28‐tetrahydroxycalix[4]arene **3** by following known procedures [35, 36]. For the introduction of the four phenyl groups, **3** was treated with an excess of phenylboronic acid in the presence of tetrakis(triphenylphosphine) palladium(0) [37]. The intermediate **4** was sulfonated using sulfuric acid, yielding only the product with the sulfonic acid groups in the 4‐positions of the phenyl moieties without substantial formation of regioisomeric byproducts [33]. In the final step, this tetrasulfonic acid was converted into the corresponding sodium salt by ion exchange.

**SCHEME 2:**
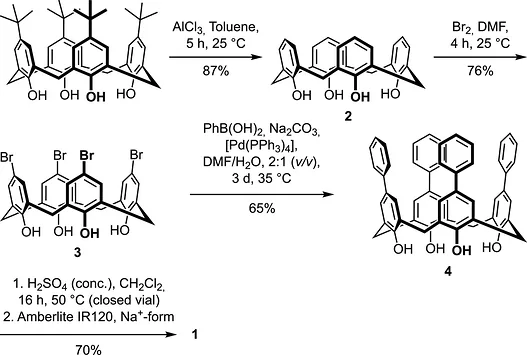
Synthesis of **1**.

The ^1^H NMR spectrum of **1** recorded in CD_3_OD is shown in Figure 1a. This spectrum was consistent with the expected averaged *C*_4v_ symmetric structure. The signal of the methylene protons at 4.1 ppm (red dot) was broad, presumably indicating a slow cone‐to‐cone inversion, but the three aromatic signals were sharp. The two multiplets (apparent doublets of the AA'XX' system, blue dots) belong to the protons of the *para*‐substituted phenylene groups, while the singlet (green dot) belongs to the protons of the inner benzene units.

**FIGURE 1:**
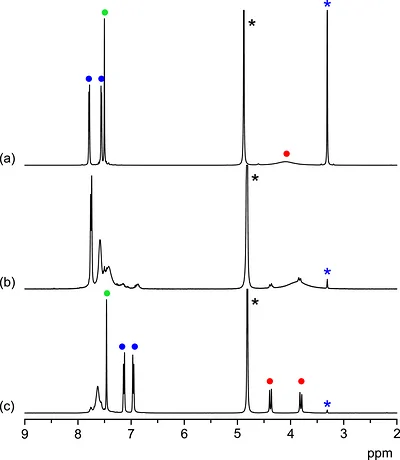
^1^H NMR spectrum of **1** recorded in CD_3_OD (a), 1 vol% CD_3_OD in D_2_O (b), and 1 vol% CD_3_OD in D_2_O in the presence of 10 molar equiv of CHCl_3_ (c). The signals of residual water and solvent protons are marked with black and blue asterisks, respectively. Dots indicate the signals of H^b^ and H^c^ (blue), H^a^ (green), and the methylene protons H^d^ and H^e^ (red) of **1**.

A more complex signal pattern was observed when the ^1^H NMR spectrum was recorded in D_2_O (containing 1 vol% of CD_3_OD as an internal standard) (Figure 1b). Under these conditions, not only the methylene signal but also the aromatic signals were broadened. Small, sharper signals were also present, which were absent in spectra recorded in D_2_O that did not contain CD_3_OD, or in neat CD_3_OD. The broadening of the signals suggested an influence of the solvent on the conformational equilibrium of **1** and/or an aggregation. Accidentally, we also recorded a spectrum of a D_2_O solution containing traces of CHCl_3_ and were surprised to see that the spectrum simplified under these conditions (Figure 1c). A few broad signals remained in the aromatic region, but all other signals were sharp, mirroring the signal pattern observed in CD_3_OD. Notably, the methylene protons produced two distinct doublets, indicating that the cone‐to‐cone inversion was substantially slowed down. In addition, the aromatic signals assigned to the protons in the outer benzene units appeared at a significantly higher field than in the spectrum obtained in CD_3_OD. These spectral changes suggested an interaction between CHCl_3_ and the calix[4]arene. Unfortunately, more detailed information could not be obtained, which is why we decided to screen other apolar guests, such as benzene, as well as cyclic and acyclic alkanes.

### 2.2 Binding Studies With Benzene

The binding studies with benzene benefited from its water solubility, which allowed us to precisely control the ratio of host and guest in the aqueous solution. ^1^H NMR spectroscopic binding studies were performed by adding between 0.5 and 3.0 molar equiv of benzene to a 4 mM solution of **1** in 1 vol% CD_3_OD in D_2_O. The spectra obtained are shown in Figure 2.

**FIGURE 2:**
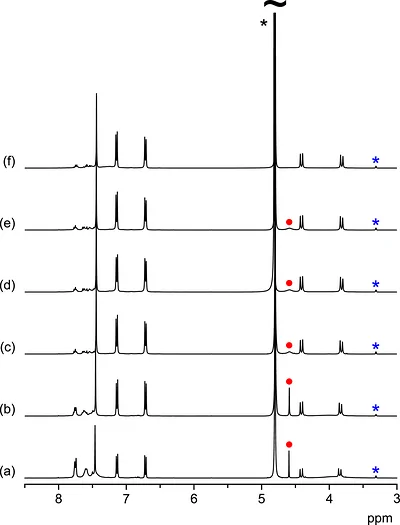
^1^H NMR spectra of **1** recorded in 1 vol% CD_3_OD in D_2_O in the presence of 0.5 (a), 1.0 (b), 1.5 (c), 2.0 (d), 2.5 (e), and 3.0 (f) molar equiv of benzene. The signal of the bound benzene is marked with a red dot, and the signals of residual water and solvent protons are marked with black and blue asterisks, respectively.

When only 0.5 molar equiv of benzene was present, the calixarene signals partially sharpened, although a few of the signals remained broad as in the spectrum recorded in D_2_O. The sharp signals resembled those observed in the spectrum with chloroform. An additional signal appeared at 4.60 ppm, which we assigned to the signal of the bound guest. In the absence of **1**, the chemical shift of the benzene protons was 7.43 ppm (Figure S1). Thus, the benzene signal moved upfield by 2.83 ppm.

The spectrum obtained in the presence of 1 molar equiv of benzene was similar, but the broad calixarene signals were less pronounced. Notably, the integral ratio of the calixarene signals and the bound benzene signal suggested that two molecules of **1** were bound to one molecule of benzene in the form of a benzene⊂(**1**)_2_ complex. As the benzene concentration increased, the signal of bound benzene broadened and disappeared at higher equivalents. We attribute this behavior to an interplay of thermodynamics and kinetics. The formation of the benzene⊂(**1**)_2_ is a two‐step process involving the initial formation of the 1:1 benzene⊂**1** complex. The NMR spectra in Figure 2 (and our other binding studies) indicate that there is a large thermodynamic driving force for the formation of the higher complex. However, this complex can only form to the extent that the 1:1 complex is present. Since the binding of benzene to one molecule of **1** is presumably weak in water, substantial formation of this complex and the subsequent formation of the benzene⊂(**1**)_2_ complex require high benzene concentrations. However, the rate of guest exchange becomes faster as the amount of free guest in solution increases, explaining the broadening and disappearance of the signal of bound guest at higher benzene concentrations.

The stability of the benzene complex was quantified using isothermal titration calorimetry by titrating a benzene solution to a solution of **1** in water. The thermogram in Figure 3 illustrates that complex formation was exothermic. The corresponding isotherm had an inflection point at 0.5, confirming the expected stoichiometry of the benzene⊂(**1**)_2_ complex. The single sharp transition of the curve indicated a cooperative binding process with no major amounts of intermediate species. Because of this sharp transition, no attempts were made to obtain the stepwise equilibria constants. Fitting the isotherm to a 1:2 substrate/receptor model with SupraFit [38] yielded a log *K*_a_ of 7.7 ± 0.1 (Δ*G*° = –44.2 ± 0.6 kJ mol^−1^) for the overall binding process, a binding enthalpy Δ*H*° of –55.1 ± 1.7 kJ mol^−1^ and a binding entropy *T*Δ*S*° of –10.9 ± 1.2 kJ mol^−1^. The negative binding enthalpy combined with an adverse entropic term is typical for binding processes in water and indicates that hydrogen bonds between water molecules, which had to be given up when hydrating the individual binding partners, were recovered upon complex formation (nonclassical hydrophobic effect) [39, 40]. Since we only determined the *K*_a_ of the overall binding process, we have no information about the magnitude and ratio of the stepwise binding constants. However, given the steep ITC isotherm with a clear inflection point at 0.5, it is reasonable to assume that the second binding constant is significantly larger than the first.

**FIGURE 3:**
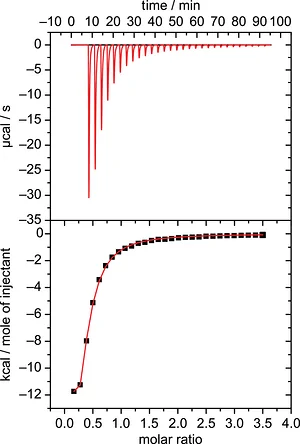
Thermogram and binding isotherm obtained by isothermal titration calorimetry for the complex of **1** with benzene in H_2_O at 298 K.

### 2.3 Binding Studies With Cycloalkanes

Due to the low solubility of cycloalkanes in water, we performed binding studies with cyclopentane, cyclohexane, cycloheptane, and cyclooctane by adding 10 molar equiv of a neat cycloalkane to the 4 mM stock solution of **1**. The mixture was shaken at room temperature for 1 h to ensure efficient mixing of the immiscible liquids. However, selected measurements indicated that complex formation was instantaneous, and the time delay was probably not required. Figure 4 shows the ^1^H NMR spectra recorded of these solutions.

**FIGURE 4:**
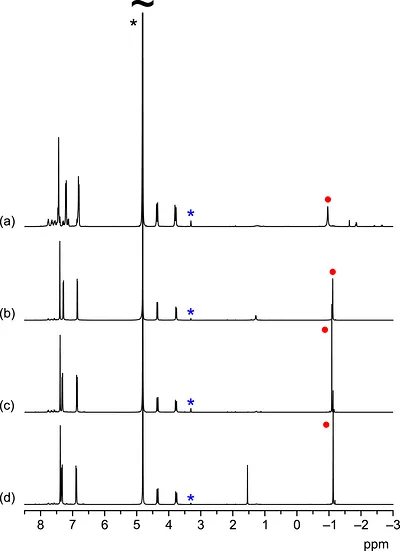
^1^H NMR spectra of **1** recorded in 1 vol% CD_3_OD in D_2_O in the presence of cyclopentane (a), cyclohexane (b), cycloheptane (c), and cyclooctane (d). The signals of residual water and solvent protons are marked with black and blue asterisks, respectively. The proton signals of the bound guests are marked with red dots.

All spectra featured one prominent set of signals, consistent with the symmetrical structure of **1** and reminiscent of the spectrum obtained in chloroform‐containing D_2_O. In addition, a singlet was consistently observed in the negative chemical shift region, suggesting the complexation of the cycloalkanes. Note that these spectral changes only occurred in water. When the ^1^H NMR spectra were recorded in CD_3_OD, practically no changes were observed when **1** was mixed with cyclooctane or *n*‐hexane (Figures S39 and S40), clearly demonstrating that the interactions in water were driven by the hydrophobic effect.

Signal integration showed that the alkane/calixarene ratio was 1:2 in all cases (Figures S4–S7), consistent with the encapsulation of the cycloalkanes inside a dimeric capsule with the composition cycloalkane⊂(**1**)_2_. All spectra, particularly those obtained with cyclopentane, exhibited small sets of signals in the aromatic region with complex signal patterns. The cyclopentane spectrum also contained minor signals close to the signal of bound guest. The comparison with the spectrum recorded in the presence of *n*‐pentane (*vide infra*) showed that these signals were unlikely to be caused by traces of the respective linear alkane. According to a COSY spectrum, they belonged to at least three additional complex species. Thus, at least four different complexes were formed when cyclopentane was present, of which the one producing the singlet at –0.96 ppm dominated. All complexes were in slow exchange, producing separate signals in the NMR spectra.

The origin of the small, strongly upfield signals in the cyclopentane spectrum is currently unclear. It cannot be ruled out that they originated from impurities in the cycloalkane. Alternatively, they could indicate the co‐encapsulation of another guest in the capsule containing the smallest cycloalkane. Calculations indicate that co‐encapsulation of one water molecule is indeed possible and associated with a favorable packing coefficient (*vide infra*). The small signals in the aromatic regions of the spectra, which were barely visible in some cases, could also not be assigned due to their complexity. Further studies are necessary to elucidate the nature of these minor species. In this work, we focused on investigating the predominant binding mode of **1**, which, according to the spectra in Figure 4, involved the encapsulation of the cycloalkanes by two subunits of the calixarene.

All guests produced singlets after complex formation, indicating that they retain sufficient mobility and flexibility in their complexes to render all protons NMR spectroscopically indistinguishable. A signal of the free guest was only clearly visible in the spectrum of cyclooctane, presumably because of evaporation of unbound guest molecules in the other samples. To assess the extent to which the cycloalkane signals were shifted when binding to **1**, their chemical shifts in the absence of **1** were determined in independent measurements. Table 1 summarizes the results.

**TABLE 1:** Chemical shifts in ppm of the calixarene and cycloalkane protons in the spectra in Figure 4. The chemical shifts of the free alkanes were determined using the same procedure but without the addition of **1**. For the signal assignment, see Scheme 1. The chemical shifts of the apparent doublets of the aromatic protons and the doublets of the methylene protons are specified as the center of the two signals.

	δ(H^a^)	δ(H^b^)	δ(H^c^)	δ(H^d^)	δ(H^e^)	δ(free guest)	δ(bound guest)	Δδ^a^
Cyclopentane	7.44	6.81	7.21	4.37	3.79	1.48	−0.96	2.44
Cyclohexane	7.40	6.85	7.29	4.36	3.77	1.40	−1.12	2.52
Cycloheptane	7.39	6.86	7.32	4.35	3.78	1.53	−1.09	2.62
Cyclooctane	7.39	6.89	7.33	4.35	3.77	1.54	−1.13	2.67

^a^Δ*δ* = *δ*(free guest)–*δ*(bound guest)

According to Table 1, the aromatic signals of **1** shifted continuously as the guest became larger. The Δ*δ* of the guest signal increased in the same direction. However, both effects were small, suggesting that the environment of the guests inside the capsule remained similar, despite their different sizes. Thus, these complexes differ from the cycloalkane complexes of a cylindrical cavitand reported by Rebek, for which pronounced size‐dependent Δ*δ* values were observed because the larger cycloalkanes occupied space in the periphery of the cavity where the shielding effects were less pronounced than at the bottom [41, 42]. In the case of **1**, all cycloalkane protons experienced the same environment on average, due to the dimeric nature of the capsules and the tumbling of the guests.

To obtain structural information about the complexes, we recorded ROESY spectra. In these spectra, the expected intramolecular cross peaks between calixarene protons were observed that allowed the assignment of the pseudoaxial and pseudoequatorial methylene protons. In addition, all four ROESY spectra contained intermolecular cross peaks between the signal of the bound guest and the three signals of the aromatic protons of **1**. As an example, the relevant region of the ROESY spectrum obtained for the cyclooctane complex is depicted in Figure 5a. For the other ROESY spectra, see Figures S8–S11.

**FIGURE 5:**
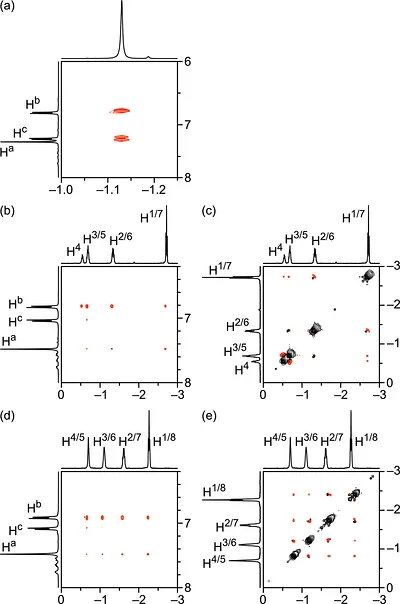
Sections of the ROESY spectra of **1** (4 mM) treated with 10 molar equiv of cyclooctane (a), *n*‐heptane (b, c), and *n*‐octane (d, e) in 1 vol% CD_3_OD in D_2_O (*T* = 298 K, mixing time 500 ms) The spectra (a), (b), and (d) show the cross peaks between guest signals and the aromatic signals of **1**. The spectra (c) and (e) show the intramolecular cross peaks of the *n*‐heptane and *n*‐octane signals, respectively.

The strongest cross peak was always to H^b^, but the intensity difference between the three cross peaks was only pronounced for cyclopentane and cyclohexane and almost disappeared for cyclooctane. These cross peaks strongly suggested a spatial proximity of the cycloalkanes to both the inner and outer aromatic units of **1** but did not indicate a specific binding mode, presumably because the cyclic guests rotate inside the capsule relatively freely. In the spectra containing well separated aromatic signals (cyclopentane, cyclohexane) cross peaks were also observed between the H^a^ and H^c^ signals, suggesting that two subunits of **1** interdigitated when forming the complex, bringing the H^a^ protons close to the H^c^ protons of the other capsule half, and *vice versa*. This interdigitation was presumably also the cause for the upfield shift of the aromatic calixarene signals upon capsule formation. Interestingly, the ROESY spectrum of the cyclopentane complex also showed cross peaks between the minor signals of **1** and the cycloalkane, confirming that at least some of these signals were likely caused by small amounts of additional complex species (Figure S8b).

We also recorded DOSY spectra of the cycloalkane complexes (Figures S12–S15). As an example, the corresponding spectrum of the cyclooctane complex is shown in Figure 6. In all cases, we observed that the species producing the signals assigned to the capsule and the bound guest diffused together, confirming that they were part of the same complex. The species producing additional minor signals in the aromatic region of the spectra diffused slightly faster, suggesting that they could be partly produced by free calixarenes or 1:1 complexes. The diffusion coefficient of the capsule was practically independent of the cycloalkane that was bound (Table S2). Thus, the structure of the capsule was apparently not strongly affected by the size of the guest. With respect to the bound guest, cycloheptane and cyclooctane had the same diffusion coefficients as the capsule after complex formation. The two smaller cycloalkanes diffused slightly faster than the capsule, which we attribute to exchange processes with free cycloalkanes or cycloalkanes in the additional liquid phase. We also quantitatively determined the hydrodynamic radius of the cyclooctane capsule by recording a DOSY spectrum in the presence of a 6G‐PAMAM‐OH dendrimer with a known diameter (Figure S16) [43]. According to this measurement, the cycloctane⊂(**1**)_2_ capsule had a hydrodynamic diameter of 3.2 nm.

**FIGURE 6:**
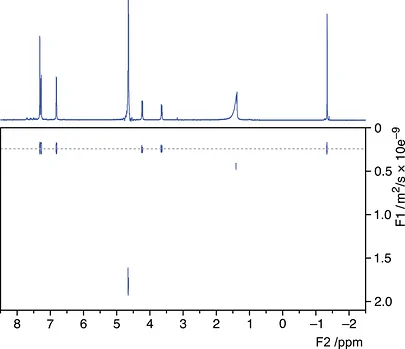
DOSY spectrum of **1** (4 mM) treated with 10 molar equiv of cyclooctane in 1 vol% CD_3_OD in D_2_O (*T* = 300 K).

We qualitatively established a sequence of complex stability using NMR spectroscopic competition measurements. To this end, the NMR samples used to record the spectra in Figure 4, which contained 10 molar equiv of a cycloalkane, were treated with an additional 10 molar equiv of the next higher cycloalkane. In Figure 7, the ^1^H NMR spectra of the individual cyclopentane and cyclohexane complexes of **1** are compared with the spectrum of the mixture. As can be seen, only the signals of the cyclohexane complex are visible in the latter spectrum, indicating that the cyclohexane complex had entirely replaced the cyclopentane complex. Although partial evaporation of the cycloalkanes during equilibration cannot be completely ruled out, a sample containing only cyclopentane, when treated under otherwise identical conditions, still showed the typical NMR spectroscopic signature of the complex. Therefore, we concluded that the cyclohexane complex is more stable than the cyclopentane complex. Similar results were obtained in the other measurements: the next higher cycloalkane always outcompeted the smaller one (Figures S17, S18). Consequently, the stability of the complexes gradually increased from cyclopentane to cyclooctane.

**FIGURE 7:**
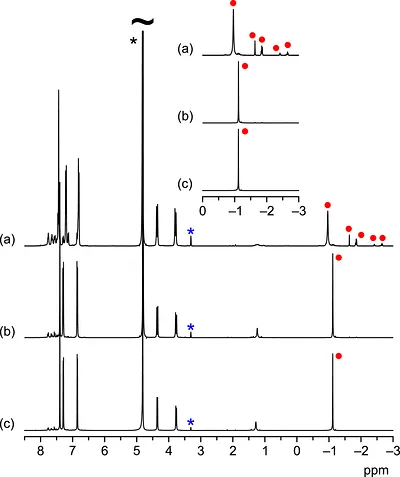
^1^H NMR spectra of **1** (4 mM) in 1 vol% CD_3_OD in D_2_O treated with 10 molar equiv of cyclopentane (a), cyclohexane (c), and cyclopentane followed by the addition of 10 molar equiv of cyclohexane (b). The inset shows the enlarged region of the spectra containing the signals of the bound guest. The signals of residual water and solvent protons are marked with black and blue asterisks, respectively. The proton signals of the bound guest are marked with red dots.

For benzene, we assessed the effect of cyclohexane and cycloheptane on the ^1^H NMR spectrum of **1** in the presence of 10 molar equiv of benzene. In both cases, the addition caused the appearance of the signal of the bound cycloalkane (Figures S2 and S3). In addition, the signals of the aromatic calixarene protons and those of the methylene protons shifted to the positions observed in the spectra recorded in the presence of cyclohexane and cycloheptane. Thus, the cycloalkanes caused the dissociation of the benzene complex, suggesting that the cycloalkane complexes were more stable.

### 2.4 Binding Studies With Linear Alkanes

In the binding studies with acyclic guests, *n*‐alkanes ranging from *n*‐pentane to *n*‐decane were used. As with the cycloalkanes, ^1^H NMR spectra were recorded of solutions of **1** in 1 vol% CD_3_OD in D_2_O to which 10 molar equiv of an alkane were added. The ^1^H NMR spectra obtained are shown in Figure 8 and Figures S19–S24.

**FIGURE 8:**
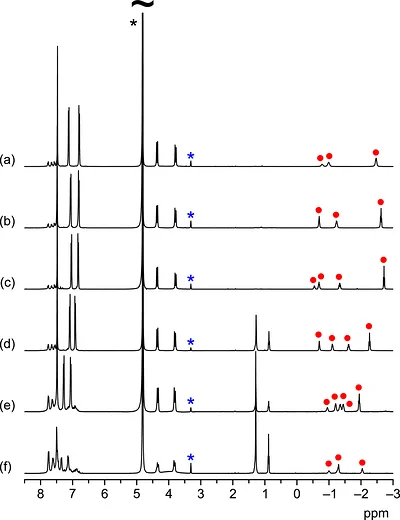
^1^H NMR spectra of **1** recorded in 1 vol% CD_3_OD in D_2_O in the presence of *n*‐pentane (a), *n*‐hexane (b), *n*‐heptane (c), *n*‐octane (d), *n*‐nonane (e), and *n*‐decane (f). The signals of residual water and solvent protons are marked with black and blue asterisks, respectively. The proton signals of the bound guest are marked with red dots.

The spectra were similar to those obtained in the presence of cycloalkanes (Figure 4), featuring sharp signals for the calixarene protons, which account for conformational stabilization and capsule formation in the presence of the guests. Additionally, a series of signals in the negative ppm range was observed in each spectrum, which were assigned to the bound alkanes. These signals were sharp for *n*‐hexane, *n*‐heptane, and *n*‐octane and broader for the shorter and longer guests, suggesting an intermediate exchange rate and presumably a lower stability of the corresponding complexes. Signal integration was consistent with an alkane⊂(**1**)_2_ stoichiometry of the complexes, except for *n*‐nonane and *n*‐decane. In the case of these two alkanes, the signal pattern produced by **1** was complex, especially in the aromatic region, suggesting that these alkanes were too large to be encapsulated efficiently between two subunits of **1**.

The spreading out of signals produced by protons along an aliphatic chain is a typical effect of guest confinement in a receptor cavity and accounts for the coiling and/or bending of the chain to adapt to the available space. This phenomenon has been observed previously for alkane complexes, such as those of Rebek's cavitands [44] and Gibb's octa acid [45]. The extent to which the protons were shielded provided information about the binding mode. In the complexes of **1**, shielding was greatest for the terminal end groups of the alkanes and significantly smaller for protons in the middle of the chain. Thus, the end groups were presumably located closer to the bottom of the bowl‐shaped cavity of **1**, while the aliphatic chain resided closer to the center region.

The chemical shifts of the calixarene signals in the *n*‐alkane complexes, as well as the extent of the complexation‐induced shifts of the methyl signal of the guest, are summarized in Table S1. The chemical shifts of **1** were comparable to those in Table 1, suggesting that the capsules containing cycloalkanes and *n*‐alkanes had similar structures. However, the extent to which the methyl signal of the guest shifted upon complex formation was substantially greater for some *n*‐alkanes than the signal shifts observed for the cycloalkanes. For instance, the methyl signal shifted upfield by 3.48 and 3.57 ppm upon encapsulation of *n*‐hexane and *n*‐heptane, respectively, whereas the signals of cyclohexane and cycloheptane shifted only by 2.52 and 2.62 ppm. This difference presumably had structural reasons. In the case of the cycloalkanes, the magnetic anisotropy of the aromatic subunits surrounding the cavity influenced all guest protons equally. By contrast, the arrangement of the linear alkanes inside the cavity was probably more defined, as suggested by the large spreading of the signals in the ^1^H NMR spectra. Thus, the *n*‐alkanes filled the available space completely, with their methyl groups likely spending a significant amount of time deep inside the calixarene cavities. This effect was most pronounced for *n*‐heptane, where the methyl protons exhibited the largest upfield shift. These shifts progressively decreased for alkanes shorter or longer than *n*‐heptane.

ROESY spectra were recorded of the *n*‐heptane and *n*‐octane complexes of **1**. In both spectra, intramolecular cross peaks between the guest signals were observed, which account for a restricted conformational mobility of the alkanes and a prevalence of arrangements in which remote protons along the chain come together (Figure 5c,e and Figures S25 and S26). The guest signals also had intermolecular cross peaks with the aromatic proton signals of **1**. For both alkanes, the cross peaks to the H^b^ signal were the strongest, as also observed for the cycloalkanes. In contrast, the cross peaks to the outer protons H^c^ were weak and only clearly visible for protons in the middle of the chain (H^3/5^ for *n*‐heptane, and H^3/6^ and H^4/5^ for *n*‐octane). The cross peaks to H^a^ had an intermediate intensity and showed a clear trend, becoming weaker with increasing distance from the methyl groups. Thus, the ROESY spectrum indicated that the terminal methyl groups were closest to the inner aromatic units of **1**, consistent with the large upfield shift they experienced upon encapsulation, while the middle parts of the chains were closer to the peripheral aromatic units. The DOSY spectrum of the *n*‐heptane complex confirmed that the capsule and the bound guest were diffusing together (Figure S27). The diffusion coefficient of the capsule was the same as that observed for the capsules of the cycloalkanes (Table S2). The bound *n*‐heptane diffused slightly slower, which we again attributed to exchange processes.

Competition experiments were performed to evaluate the stability of the *n*‐alkane complexes by adding the next higher *n*‐alkane homolog or the corresponding cycloalkane to an *n*‐alkane complex. In the experiments with *n*‐alkanes (Figures S28–S32), we observed that adding *n*‐hexane to the *n*‐pentane complex shifted the equilibrium almost entirely toward the *n*‐hexane complex. However, minor amounts of the *n*‐pentane complex were still visible after equilibration. According to signal integration, the ratio of the *n*‐pentane and *n*‐hexane complexes was approximately 1:6. Because the solubility of the two alkanes in water differs, and the exact amount of alkane in the aqueous solution may also be affected by the volatility of the guests, this ratio does not necessarily provide quantitative information about the relative stabilities of the two complexes. However, the presence of both complexes in the spectrum suggested that their stability difference is smaller than if one complex had completely outcompeted the other, as in the case of cyclopentane and cyclohexane.

The NMR spectrum of the *n*‐hexane–*n*‐heptane mixture also clearly showed that both complexes were present, this time with a ratio of approximately 3:1 in favor of the *n*‐hexane complex. The other spectra showed that *n*‐heptane outcompeted *n*‐octane and that *n*‐octane outcompeted *n*‐nonane and *n*‐decane. Thus, the stability of the complexes between **1** and linear alkanes appeared to peak at *n*‐hexane, while the complexes of the shorter and longer alkanes were less stable.

Consistent with this sequence and the previously observed stability order of the cycloalkane complexes, the competition experiments with linear and cyclic alkanes (Figures S33–S36) showed that the *n*‐pentane complex was more stable than the cyclopentane complex. Interestingly, while the cyclopentane complex was completely absent in the corresponding NMR spectrum, signals of the minor cyclopentane complexes were still visible. Thus, these complexes seem to be more stable than the predominant cyclopentane complex. In the competition experiment with *n*‐hexane and cyclohexane, both complexes were visible at an *n*‐hexane/cyclohexane ratio of 2:1, indicating the *n*‐hexane complex is more stable than the cyclohexane complex. For heptane, the order reversed in favor of the complex of the cyclic alkane, with only traces of the *n*‐heptane complex visible. The stable cyclooctane complex completely outcompeted the *n*‐octane complex.

### 2.5 Binding Studies With Fluorinated Guests

We also investigated whether **1** encapsulates fluorinated compounds using hexafluorobenzene and perfluoro‐*n*‐hexane as representative guests. The presence of hexafluorobenzene in an aqueous solution of **1** caused the calixarene signals to sharpen, as also observed for the nonfluorinated alkanes (Figure S37). Additionally, the hexafluorobenzene signal shifted upfield in the ^19^F NMR spectrum from –164.5 ppm in the absence to –164.7 ppm in the presence of **1** (Figure S38). These effects were consistent with an encapsulation of hexafluorobenzene, although a stoichiometry of the complex could not be derived because of the absence of a signal of the guest in the ^1^H NMR spectrum. After adding benzene to the solution of the hexafluorobenzene complex, the aromatic and methylene signals of **1** shifted to the positions observed in the spectrum of the benzene complex. Thus, the hexafluorobenzene complex dissociated in the presence of benzene, leading to the formation of the presumably more stable benzene complex. Perfluoro‐*n*‐hexane did not produce changes in the ^1^H NMR spectrum when added to **1**, indicating that the linear fluorinated alkane was not bound.

### 2.6 Computational Characterization of Capsule Formation

Figure 9 illustrates the general architecture of the capsules obtained computationally. As shown in Figure 9a for the empty capsule (**1**)_2_, the two calixarene subunits of **1** (distinguished in blue and green) interdigitate extensively along their upper rims, giving rise to a closed, roughly ellipsoidal capsule with overall dimensions of 16.4 × 20.4 Å. These distances were measured from the most distant O‐atoms in two perpendicular directions without considering van der Waals radii. Considering that the cages are dynamic in solution and extensively hydrated due to the negatively charged groups, the larger diameter determined experimentally by DOSY is in reasonable agreement.

**FIGURE 9:**
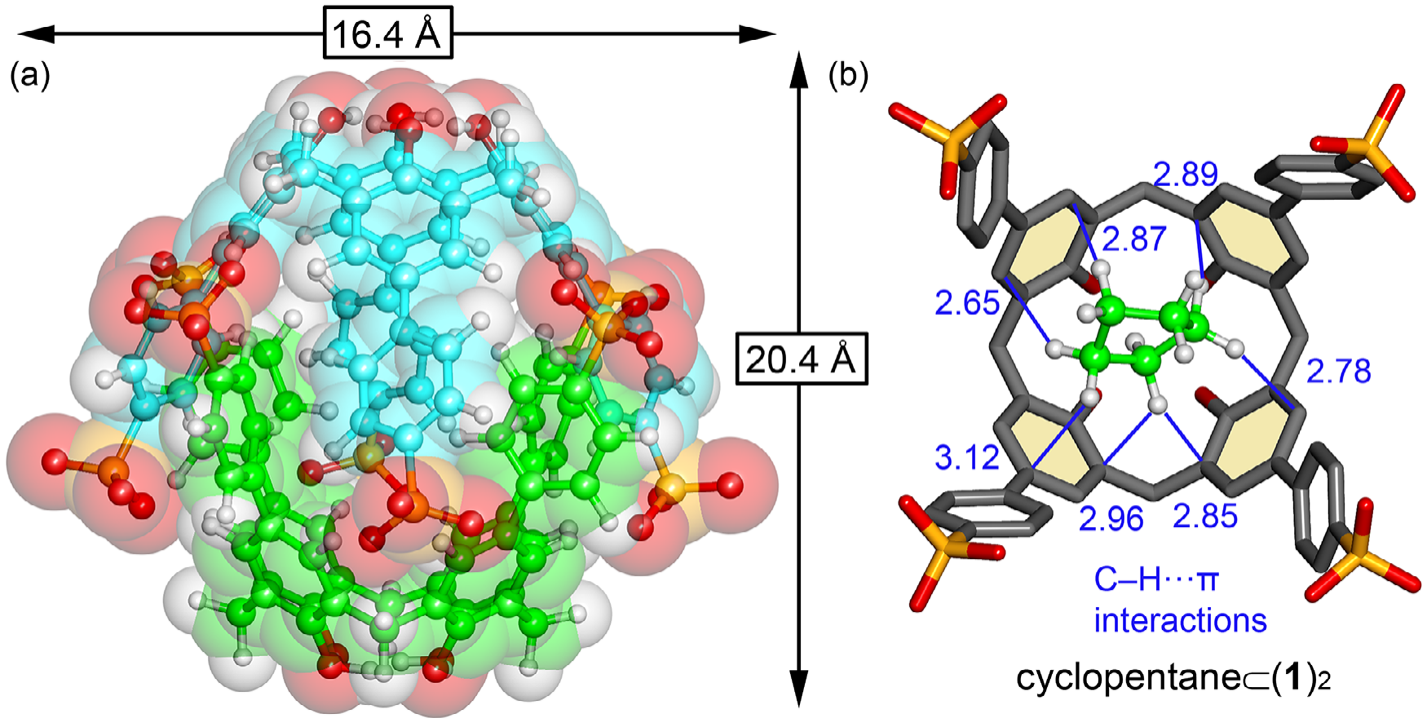
(a) Optimized structure of the capsule (**1**)_2_, shown as a transparent CPK/ball‐and‐stick overlay with the two calixarene subunits of **1** distinguished in blue and green, illustrating the interdigitation of the aromatic walls and the overall dimensions of the capsule (16.4 × 20.4 Å). (b) CH···π contacts (Å) in the cyclopentane complex, shown for one calixarene subunit only for clarity. Distances to the nearest C‐atom of the ring are indicated in Å.

The interdigitation of the two capsule halves creates an elongated hydrophobic cavity in which the guests are encapsulated. Figure 9b highlights the network of CH···π contacts between a guest and the aromatic walls of **1**, using cyclopentane⊂(**1**)_2_ as a representative example and showing one calixarene subunit only for the sake of clarity. Multiple such contacts are observed, with H···C distances ranging from 2.65 to 3.12 Å, consistent with the CH···π interactions generally invoked to rationalize the encapsulation of hydrophobic guests by this class of host.

To obtain a computational counterpart to the experimentally derived stability sequence and to gain further insight into the structural details of complex formation, the complexes of **1** with all cyclic and acyclic alkanes as well as with benzene and hexafluorobenzene were optimized at the BP86‐D4(COSMO = water)/def2‐SVP level of theory (see Supporting Information for details). Because the pronounced conformational flexibility of the monomeric calixarene subunits precludes a reliable direct calculation of binding or interaction energies, the relative affinity of the different guests for the capsule was instead assessed from substitution energies (Δ*E*_sub_) calculated for the reaction guest⊂(**1**)_2_ + *n*‐octane → *n*‐octane⊂(**1**)_2_ + guest, using the *n*‐octane complex as internal reference. Because both sides of this formal substitution reaction involve a guest‐filled capsule of similar overall composition, contributions from conformational flexibility, solvation, and entropy largely cancel, so that the calculated Δ*E*_sub_ values approximate relative rather than absolute binding energies. A negative Δ*E*_sub_ value indicates that substitution of the guest by *n*‐octane is thermodynamically favorable, that is, that the *n*‐octane complex is more stable than the complex of the guest in question, whereas a positive value indicates the opposite. In addition to Δ*E*_sub_, Table 2 lists the packing coefficients of the complexes, that is, the percentage of the interior capsule volume occupied by the guest, which provides a purely geometric, independent measure of the goodness of fit between host and guest.

**TABLE 2:** Interior volume of the capsule (*V*((**1**)_2_)) and volume of the guest (*V*(guest)) in each complex (both in Å^3^), packing coefficient, and substitution energy (Δ*E*_sub_, kcal mol^−1^) calculated for the reaction guest⊂(**1**)_2_ + *n*‐octane → *n*‐octane⊂(**1**)_2_ + guest at the BP86‐D4(COSMO = water)/def2‐SVP level of theory.

Guest	*V*((1)_2_) (Å^3^)	*V*(guest) (Å^3^)	Packing coefficient (%)	Δ*E*_sub_ (kcal mol^−1^)
Cyclopentane	204	82	40	−3.2
Cyclohexane	204	100	49	−4.7
Cycloheptane	213	117	55	−1.7
Cyclooctane	220	134	61	2.2
*n*‐Pentane	190	95	50	−6.3
*n*‐Hexane	218	112	51	−5.2
*n*‐Heptane	226	129	57	−3.8
*n*‐Octane	233	149	64	0.0
Benzene	192	82	43	−7.4
Hexafluorobenzene	212	98	46	−1.0

Table 2 indicates that the capsule formed by **1** can adapt the cavity volume to the included guest. However, the changes are not very pronounced, consistent with the minor differences in the complexation induced shifts observed in the ^1^H NMR spectroscopic binding studies and the comparable diffusion constants of the complexes. Δ*E*_sub_ is negative for all guests except cyclooctane, for which a positive value of 2.2 kcal mol^−1^ was obtained, identifying the cyclooctane complex as the most stable of the entire series. This agrees with the experimental finding that the cyclooctane complex completely outcompeted the *n*‐octane complex in competition experiments. For the cycloalkanes, the calculations reproduce the pronounced increase in stability from cycloheptane (–1.7 kcal mol^−1^) to cyclooctane (2.2 kcal mol^−1^), consistent with experiment, but predict cyclohexane (–4.7 kcal mol^−1^) to be marginally less stable than cyclopentane (–3.2 kcal mol^−1^), which is opposite to the experimentally established sequence cyclopentane < cyclohexane. For the *n*‐alkanes, Δ*E*_sub_ increases monotonically from *n*‐pentane (–6.3 kcal mol^−1^) to *n*‐octane (0.0 kcal mol^−1^, reference), suggesting a steady increase in stability with chain length. This trend does not reproduce the experimentally observed maximum in stability at *n*‐hexane, after which the complexes become gradually less stable for *n*‐heptane and *n*‐octane. Comparison of linear and cyclic alkanes of the same carbon number shows good agreement between calculation and experiment for the two larger pairs: both computationally and experimentally, cycloheptane is more stable than *n*‐heptane, and cyclooctane is markedly more stable than *n*‐octane. For the two smaller pairs, however, the calculations predict cyclopentane and cyclohexane to be slightly more stable than *n*‐pentane and *n*‐hexane, respectively, which is opposite to the experimentally derived order (*n*‐pentane > cyclopentane; *n*‐hexane > cyclohexane). Benzene gives the most negative Δ*E*_sub_ value of the entire series (–7.4 kcal mol^−1^), identifying its complex as the least stable one, in agreement with the observation that both cyclohexane and cycloheptane displace bound benzene in competition experiments. Hexafluorobenzene (–1.0 kcal mol^−1^), however, is calculated to form a more stable complex than benzene, whereas the experimentally observed displacement of the hexafluorobenzene complex by benzene suggests the reverse order. Together with the reversed order found for the smaller cycloalkane/*n*‐alkane pairs, this shows that, although the calculated and experimental stability sequences agree reasonably well overall, particularly for the larger guests, agreement is not perfect. This is not unexpected given that the substitution‐energy approach, while minimizing errors from conformational flexibility and solvation, still neglects entropic contributions that likely play a non‐negligible role for these highly flexible host–guest systems.

The optimized structures shown in Figure 10 rationalize the trends in the packing coefficients, which increase from 40% for the smallest guest, cyclopentane, to 64% for *n*‐octane, the largest guest included in this comparison. According to Rebek's 55% rule, a packing coefficient of about 55% should be ideal for complex formation [46]. While the packing coefficients in Table 2 are distributed around this value, there is no exact correlation between their magnitude and complex stability. Complexes of lower stability (cyclopentane, benzene, hexafluorobenzene) are associated with smaller packing coefficients <50%, but the trend is less clear for the larger guests. Especially the large packing coefficient obtained for the cyclooctane complex is not necessarily consistent with its high stability, suggesting that other factors contribute to complex stability.

**FIGURE 10:**
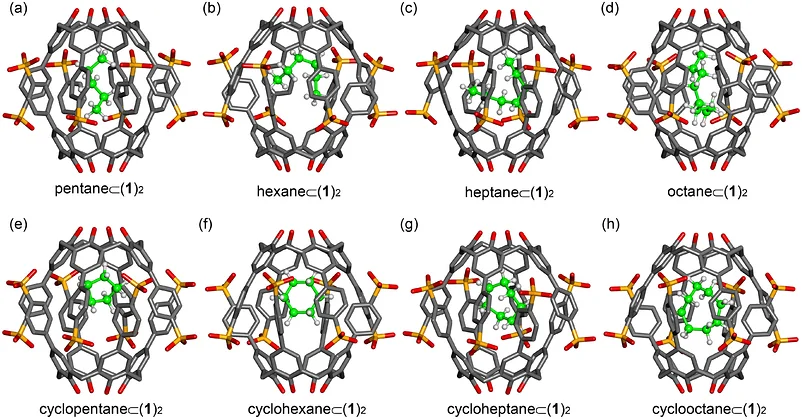
Optimized structures (BP86‐D4(COSMO = water)/def2‐SVP) of the complexes of **1** with *n*‐pentane (a), *n*‐hexane (b), *n*‐heptane (c), *n*‐octane (d), cyclopentane (e), cyclohexane (f), cycloheptane (g), and cyclooctane (h). Hydrogen atoms of **1** are omitted for clarity.

To assess whether co‐encapsulation of solvent could rationalize the residual cavity space observed for the smaller guests, we calculated the packing coefficients for cyclopentane, cyclohexane, *n*‐pentane, and *n*‐hexane in the presence of one and two co‐encapsulated water molecules. In the presence of a single water molecule, the packing coefficients for cyclopentane (53%), cyclohexane (57%), and *n*‐pentane (57%) increase substantially from their empty‐cavity values (40%, 49%, and 50%, respectively) and align remarkably well with Rebek's empirical 55% rule. By contrast, inclusion of two water molecules leads to packing values between 63% and 70%, indicating unfavorable steric congestion. These results suggest that the inclusion of an explicit, single internal water molecule is sterically optimal for the smallest guests (C5–C6), providing a geometric rationale for the minor complex species detected experimentally in the ^1^H NMR spectra of cyclopentane. Because standard continuum solvent models cannot capture the discrete thermodynamics of these ternary guest‐water assemblies or differential entropic costs, the binary substitution energies inevitably diverge from experiment for the smallest homologs. Thus, the DFT calculations serve primarily as a qualitative structural model, while the experimental NMR competition data provide the definitive benchmark for capsule stability in solution.

The complexes of the smaller guests in Figure 10 illustrate that the guest is located mainly within one calixarene subunit and interacts primarily with the aromatic walls of this half of the capsule. As guest size increases, the guest increasingly spans the length of the cavity and establishes contacts with the aromatic walls of both calixarene subunits, maximizing the number of CH···π contacts. This structural evolution parallels the ROESY data, which show intermolecular cross peaks between the guest signals and the aromatic proton signals of **1** for all guests investigated, with the largest guests showing contacts distributed over the entire aromatic surface of the capsule. In the complexes of the fluorinated guests (Figure S42), hexafluorobenzene is instead located centrally within the cavity, a position that minimizes unfavorable F···π contacts with the aromatic walls of **1** (Figure S43).

## 3 Conclusion

The tetrasulfonated deep‐cavity calix[4]arene **1** self‐assembles into a well‐defined dimeric capsule in water that encapsulates small hydrophobic guests, including linear and cyclic alkanes, and benzene. Binding studies with fluorinated hydrocarbons have only provided evidence of the complexation of hexafluorobenzene so far. ^1^H NMR spectroscopy establishes that the two calixarene subunits interdigitate to form an elongated hydrophobic cavity, and ROESY experiments show that the guests are held close to the center of this cavity through extensive contacts with the aromatic walls. Isothermal titration calorimetry and, in particular, a systematic series of NMR competition experiments allowed us to establish detailed stability sequences for both the linear and cyclic alkane guests, revealing that complex stability peaks at *n*‐hexane among the linear alkanes and increases steadily from cyclopentane to cyclooctane among the cycloalkanes, with the latter completely outcompeting the *n*‐octane complex. Related competition experiments with benzene and hexafluorobenzene further delineate the relative affinities of aromatic and fluorinated guests.

DFT calculations of substitution energies and packing coefficients provided a computational counterpart to these experimental trends and, together with the optimized structures, offered useful structural insight into how guest size and shape govern the number and nature of the CH···π contacts formed within the capsule. However, due to the conformational flexibility of the interdigitated calixarene walls, combined with the likely importance of entropic and solvation contributions that are difficult to capture within standard DFT calculations, the experimental stability sequences could only be reproduced in part, particularly for the larger guests. This underscores that the subtle, guest‐size‐dependent thermodynamics of hydrophobically driven encapsulation in water established here rest primarily on the experimental data.

In a broader context, deep‐cavity calix[4]arene **1** is not only a novel building block for producing molecular capsules in water, but also an attractive system for future work on binding processes in water. For example, this calixarene is an ideal tool to study how salts, which influence the hydration of solutes to varying degrees and in different ways, affect binding processes that are associated with the burial of hydrophobic surfaces. This work is ongoing, and the results will be reported in due time.

## Funding

This study was supported by Bundesamt für Ausrüstung, Informationstechnik und Nutzung der Bundeswehr.

## Conflicts of Interest

The authors declare no conflicts of interest.

## Supporting information



The authors have cited additional references within the Supporting Information [47, 48, 49, 50, 51, 52, 53, 54, 55].
**Supporting File 1**: chem71682‐sup‐0001‐SuppMat.zip.

**Supporting File 2**: chem71682‐sup‐0002‐SuppMat.pdf.

## Data Availability

The data that supports the findings of this study are available in the Supporting Information of this article.
