# Supplementary material for: Encapsulation of Linear and Cyclic Alkanes in Water Within a Dimeric Deep‐Cavity Sulfonatocalix[4]arene Capsule
Source: Chemistry. 2026 Sep 11;32(42):e71682. doi: 10.1002/chem.71682 (PMC13755158; doi:10.1002/chem.71682)
Supplement: Supplementary file 1 [file CHEM-32-e71682-s002.pdf]

Chem. Eur. J.

Encapsulation of Linear and Cyclic Alkanes in Water within a Dimeric Deep-Cavity Sulfonatocalix[4]arene Capsule

Nico Schneider,[a] Sebastiano Casalino,[a] Aldrik H. Velders,[b] Rosa M. Gomila,[c] Antonio Frontera,[c] Stefan Kubik*,[a]

[a] *Fachbereich Chemie – Organische Chemie, RPTU University Kaiserslautern-Landau, Erwin-Schrödinger-Str. 54, 67663 Kaiserslautern, Germany.*

[b] *Laboratory of BioNanoTechnology, Wageningen University, PO Box 8038, Wageningen 6700 EK, The Netherlands.*

[c] *Departament de Química, Universitat de les Illes Balears Crta de Valldemossa km 7.5, 07122 Palma de Mallorca, Baleares, Spain.*

CONTENT

Synthesis of **1** S2
Spectra of **1** S6
Binding Studies S9
  Binding of Benzene S11
  Binding of Cycloalkanes S14
  ROESY Spectra of Cycloalkane Complexes S18
  DOSY Spectra of Cycloalkane Complexes S23
  Competition Experiments with Cycloalkanes S28
  Binding of *n*-Alkanes S30
  ROESY Spectra of *n*-Alkane Complexes S37
  DOSY Spectrum of *n*-Heptane Complex S39
  Competition Experiments with Linear Alkanes S41
  Competition Experiments with Linear Alkanes and Cycloalkanes S46
  Binding of Hexafluorobenzene S50
  NMR Spectra in Methanol S52
ITC Measurements S54
Details of the Computational Studies S56
References S58

Synthesis of 1

General details. Analyses were carried out as follows: melting points, Müller SPM-X 300; NMR, Bruker Avance III 400 and Bruker Avance 600, the spectra were referenced to the residual solvent signals (DMSO-d_6: δ_H = 2.50 ppm, δ_C = 39.5 ppm; $CDCl_3$: δ_H = 7.26 ppm, δ_C = 77.2 ppm; CD_3OD: δ_H = 3.31 ppm, δ_C = 49.0), assignments confirmed by HSQC and HMBC spectra;[1] ESI-MS, amaZon speed ETD; elemental analysis, Elementar vario Micro cube. The cycloalkanes, *n*-alkanes, benzene, and hexafluorobenzene were purchased at the best obtainable purity: cyclopentane (TCI, ≥97.0%), cyclohexane (TCI, ≥99.5%), cycloheptane (TCI, ≥98.0%), cyclooctane (TCI, ≥98.0%), *n*-pentane (TCI, standard for GC, ≥99.5%), *n*-hexane (TCI, standard for GC, ≥99.5%), *n*-heptane (TCI, standard for GC, ≥99.5%), *n*-octane (TCI, standard for GC, ≥99.5%), *n*-nonane (TCI, standard for GC, ≥99.5%), *n*-decane (TCI, standard for GC, ≥99.5%), benzene (Carl Roth, p.a., ≥99.5%), hexafluorobenzene (Sigma-Aldrich, NMR grade, ≥ 99.5%).

25,26,27,28-Tetrahydroxycalix[4]arene[2]

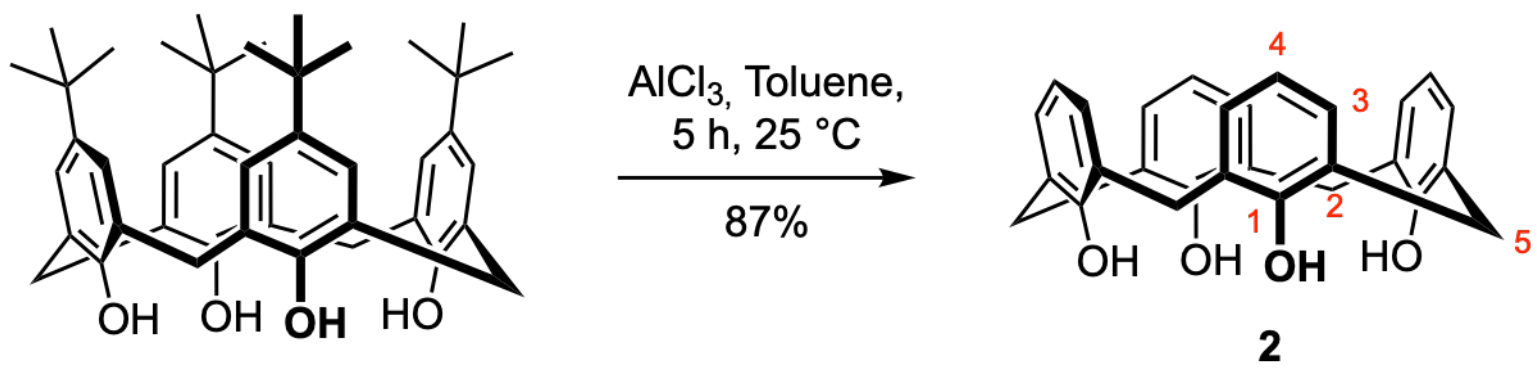

Scheme S1: Synthesis of **2**.

tert-Butylcalix[4]arene (29.9 g, 46.0 mmol, 1 equiv) and phenol (8.6 g, 92.0 mmol; 2 equiv) were suspended in toluene (460 mL). Argon was bubbled through the resulting suspension for 15 min. The suspension was stirred vigorously with a KPG stirrer, $AlCl_3$ (30.7 g, 230 mmol, 5 equiv) was added and the reaction mixture was stirred for 5 hours at 25 °C. During this time, a stream of argon was passed over the mixture and vigorous stirring was continued. The mixture was slowly added to cold hydrochloric acid (600 mL, 1 M). Solids or resins precipitated during the reaction were dissolved in a mixture of hydrochloric acid and chloroform and combined with the hydrochloride acid layer. The phases were separated and the aqueous phase was extracted twice with chloroform (2 × 200 mL). The combined organic layers were washed with hydrochloric acid (1 M, 2 × 250 mL) and H_2O (2 × 250

mL) and dried over Na_2SO_4. The solvent was removed under reduced pressure, the residue was mixed with MeOH (300 mL), and the mixture was stored overnight at –18 °C.

The precipitated solid was filtered off, washed with methanol, and dried under vacuum. According to ^{1}H NMR, the crude product contained approx. 1% non-dealkylated by-products. For purification, the solid was recrystallized from chloroform/methanol. To this end, it was dissolved in hot chloroform (10 mL chloroform/g crude product). The solution was filtered while hot and reheated to reflux. At this temperature, methanol was added until the solution became cloudy. The mixture was allowed to cool slowly and stored overnight in a freezer. The solid was filtered off, thoroughly washed with methanol, and dried.

During washing with methanol, solid material precipitated again from the mother liquor. The mother liquor was left to stand at room temperature for 5 h, the solid material was filtered off and recrystallized again as described above. A product was thus obtained that, according to ^{1}H NMR, contained only trace amounts of non-dealkylated by-products. The solid precipitated from the mother liquor during the second recrystallization was filtered off, dried and reused in a subsequent reaction batch. Yield: 17.0 g (87%, off-white solid); mp: >250 °C; ^{1}H NMR (400 MHz, $CDCl_3$, 25 °C): δ 10.21 (s, 4H, OH), 7.06 (d, ^3J = 7.6 Hz, 8H, H-3), 6.74 (t, ^3J = 7.6 Hz, 4H, H-4), 4.27 (b, 4H, H-5), 3.56 (b, 4H, H-5) ppm; ^{13}C NMR (101 MHz, $CDCl_3$, 25 °C): δ 148.9 (C-1), 129.1 (C-3), 128.4 (C-2), 122.4 (C-4), 31.9 (C-5) ppm.

5,11,17,23-Tetrabromo-25,26,27,28-tetrahydroxycalix[4]arene[3]

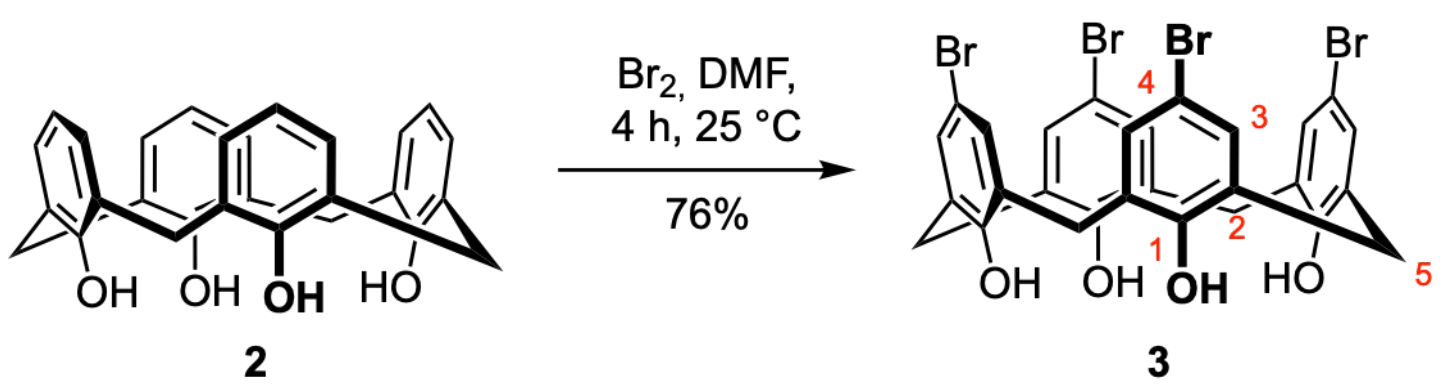

Scheme S2: Synthesis of **3**.

Calix[4]arene **2** (1.00 g, 2.36 mmol, 1 equiv) was dissolved in DMF (40 mL) and a solution of bromine (0.73 mL, 14.1 mmol, 6 equiv) in DMF (10 mL) was added dropwise. The mixture was stirred for 4 h at room temperature. Methanol (40 mL) was added, and the mixture was stirred for further 30 min at room temperature. The precipitated solid was filtered off, washed with methanol

and dried *in vacuo*. Yield: 1.33 g (76%, colorless solid); mp: >200 °C; ^1H NMR (600 MHz, DMSO-d_6, 25 °C): δ 7.84 (s, 8H, H-3), 3.80 (s, br, 8H, H-5) ppm; ^{13}C NMR (151 MHz, DMSO-d_6, 25 °C): δ 150.3 (C-1), 131.0 (C-3), 130.9 (C-2), 111.5 (C-4), 30.0 (C-5) ppm; MS (ESI–) *m/z* (%): 738.8 (100) $[M–H]^–$ (isotope pattern consistent with the presence of four bromine atoms).

5,11,17,23-Tetraphenyl-25,26,27,28-tetrahydroxycalix[4]arene[4]

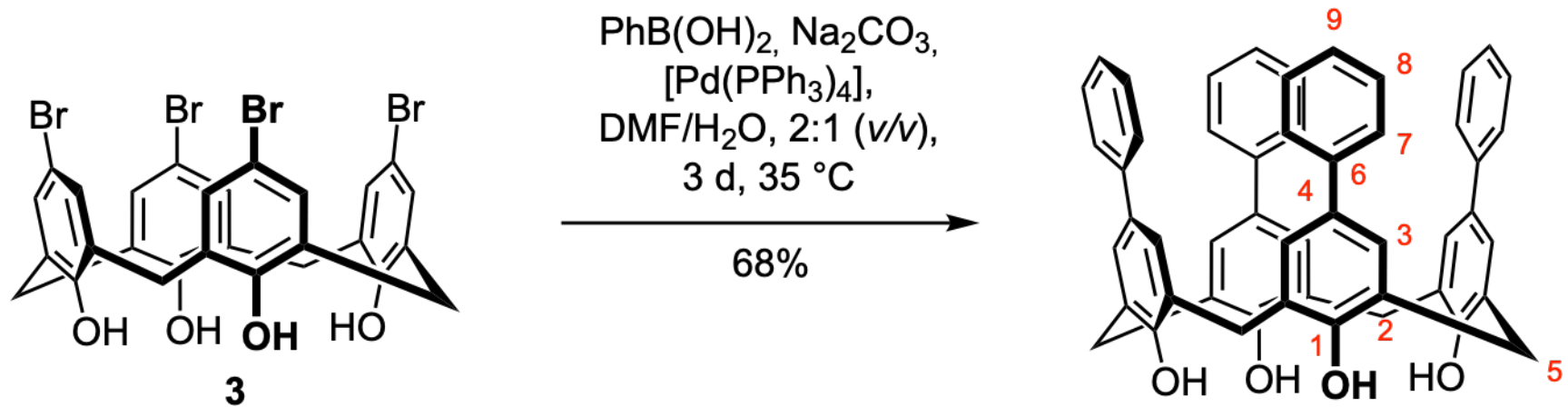

Scheme S3: Synthesis of **4**.

5,11,17,23-Tetrabromo-25,26,27,28-tetrahydroxycalix[4]arene **3** (434 mg, 0.59 mmol, 1 equiv) and phenylboronic acid (428 mg, 3.5 mmol, 6 equiv) were dissolved in degassed DMF (40 mL) under an atmosphere of nitrogen. A solution of sodium carbonate (7.43 g, 70.1 mmol, 120 equiv) in degassed H_2O (20 mL) was added, followed by $[Pd(PPh_3)_4]$ (68 mg, 0.06 mmol, 0.1 equiv). The mixture was stirred for 3 d at 35 °C under nitrogen. Afterward, chloroform (120 mL) was added, and the mixture was washed with hydrochloride acid (1 M, 2 × 60 mL), water (2 × 60 mL), brine (2 × 60 mL), and water (1 × 60 mL). The organic layer was dried over Na_2SO_4, and the solvent was removed under reduced pressure. The residue was triturated with methanol, the solid was separated by centrifugation, and dried *in vacuo*. It was purified by column chromatography (SiO_2, dichloromethane/*n*-hexane, 1:1 (*v/v*)). Yield: 290 mg (68%, colorless solid); mp: >200 °C; ^1H NMR (400 MHz, DMSO-d_6, 25 °C): δ 7.57 (s, 8H, H-3), 7.50 (apparent d (AA'MM'X), 8H, H-7), 7.36 (apparent t (AA'MM'X), 8H, H-8), 7.23 (t, ^3J = 7.3 Hz, 4H, H-9), 4.04 (s, b, 8H, H-5) ppm; ^{13}C NMR (101 MHz, DMSO-d_6, 25 °C): δ 149.5 (C-1), 140.0 (C-6), 133.4 (C-4), 129.1 (C-2), 128.6 (C-8), 127.2 (C-3), 126.5 (C-9), 126.3 (C-7), 31.0 (C-5) ppm; MS (ESI–) *m/z* (%): 727.2 (100) $[M–H]^–$, 651.2 (8) $[M–C_6H_4–H]^–$; CHN calculated for $C_{52}H_{40}O_4$ (M.W. 728.87) C, 85.69; H, 5.53; found C, 85.31; H, 5.47.

Pentasodium 5,11,17,23-tetra(4-sulfonatophenyl)-25,26,27,28-tetrahydroxycalix[4]arene[5]

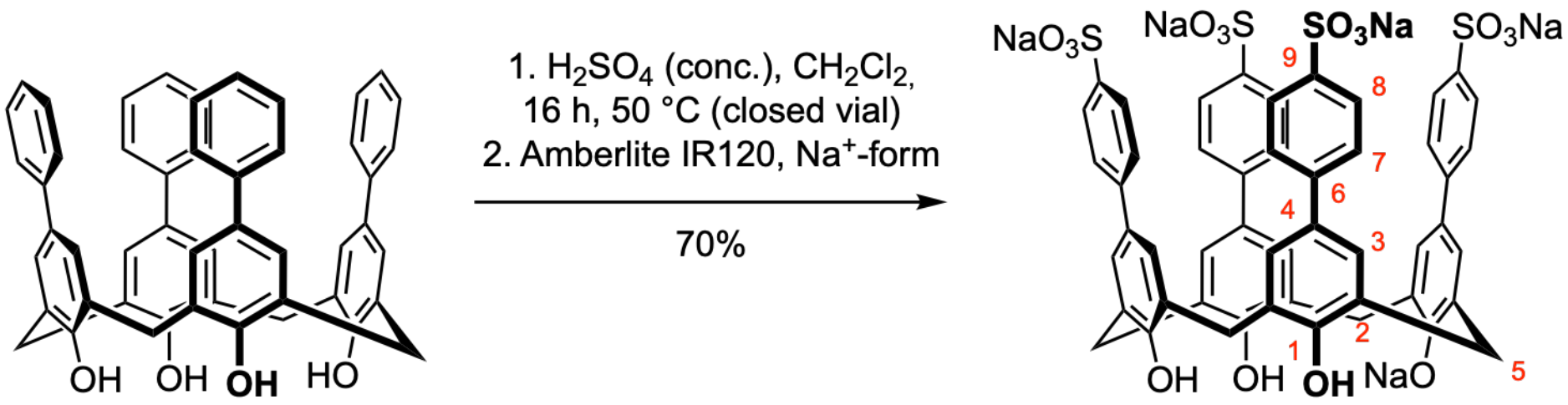

4 **1**

Scheme S4: Synthesis of **1**.

5,11,17,23-Tetraphenyl-25,26,27,28-tetrahydroxycalix[4]arene **4** (250 mg, 0.35 mmol, 1 equiv) was dissolved in dichloromethane (7 mL) and the solution was heated to 50 °C in a 50 mL headspace vial sealed with a septum. Concentrated H_2SO_4 (0.26 mL, 4.9 mmol, 14 equiv) was added and the mixture was stirred for 16 h at 50 °C. The solvent was decanted, dichloromethane (5 mL) was added, the flask shaken and the solvent decanted. This washing step was repeated two more times. The residue was then triturated with diethyl ether (5 mL) to give a powder, which was separated by centrifugation. The solid was suspended in diethyl ether (5 mL) and again separated by centrifugation. This washing step was repeated twice. The product was dried *in vacuo*. Afterward, it was dissolved in water/methanol, 1:1 (*v/v*) (2 mL), and the solution was eluted over an ion exchange resin (Amberlite IR-120, Na+ form). The fractions containing the product were combined and evaporated to dryness. The residue was dissolved in methanol (10 mL), diethyl ether (10 mL) was added, and the precipitate collected by centrifugation. The solid was suspended in diethyl ether (20 mL) and again separated by centrifugation. This washing step was repeated twice more. The product was dried *in vacuo*, dissolved in water (10 mL) and the solution was freeze-dried. Yield: 280 mg (0.24 mmol, 70%, colorless solid); mp: >200 °C; ^1H NMR (600 MHz, CD_3OD, 25 °C): δ 7.79 (apparent d (AA'XX'), 8H, H-8), 7.56 (apparent d (AA'XX'), 8H, H-7), 7.50 (s, 8H, H-3), 4.10 (s, b, 8H, H-5) ppm; ^{13}C NMR (151 MHz, CD_3OD, 25 °C): δ 152.0 (C-1), 144.1/144.0 (C-6/C-9), 134.5 (C-4), 130.9 (C-2), 128.6 (C-3), 127.4 (C-7), 127.3 (C-8), 32.8 (C-5) ppm; MS (ESI–) *m/z* (%): 260.9 (100) $[M–5Na+H]^{4–}$, 266.3 (60) $[M–4Na]^{4–}$, 355.5 (86) $[M–4Na+H]^{3–}$, 362.8 (88) $[M–3Na]^{3–}$, 544.8 (26) $[M–3Na+H]^{2–}$, 555.8 (30) $[M–2Na]^{2–}$; CHN calculated for $C_{52}H_{35}Na_5O_{16}S_4·8.5\ H_2O$ (M.W. 1312.16) C, 47.60 ; H, 3.99; S, 9.77; found C, 47.27; H, 3.62; S, 10.16.

Spectra of 1

^{1}H NMR (600 MHz, CD_3OD)

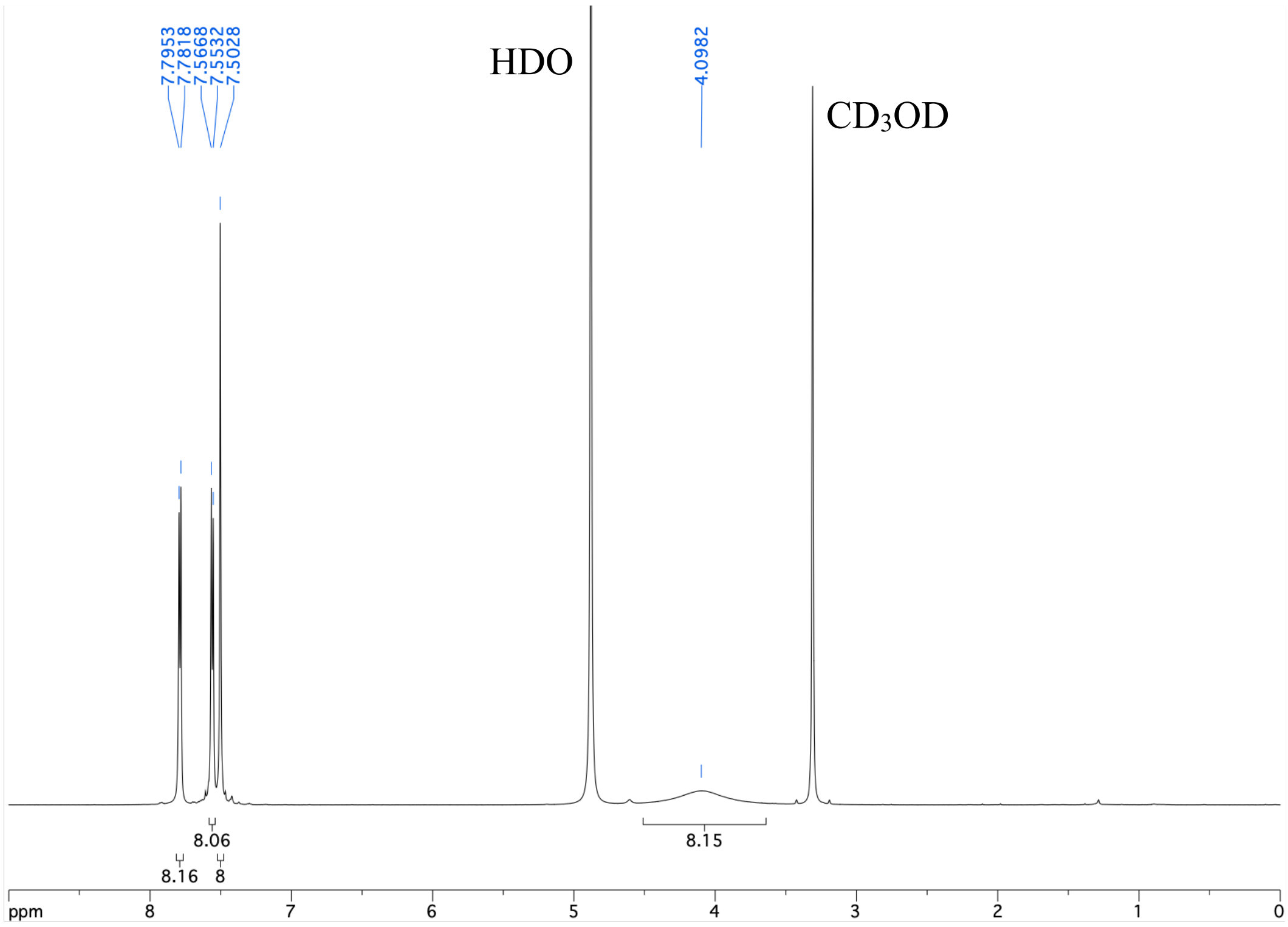

^{13}C NMR (151 MHz, CD_3OD)

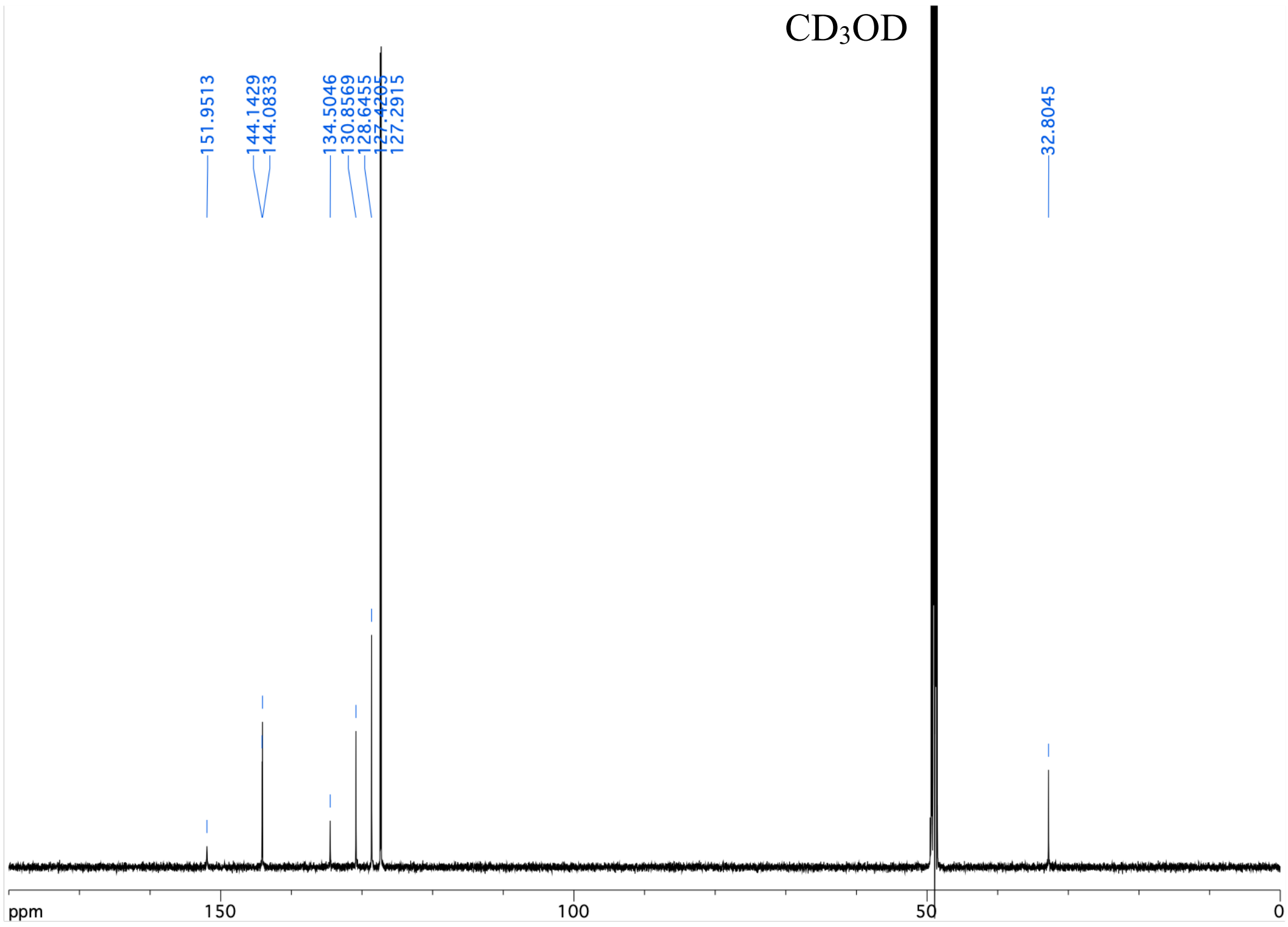

MS (ESI/TOF$^-$)

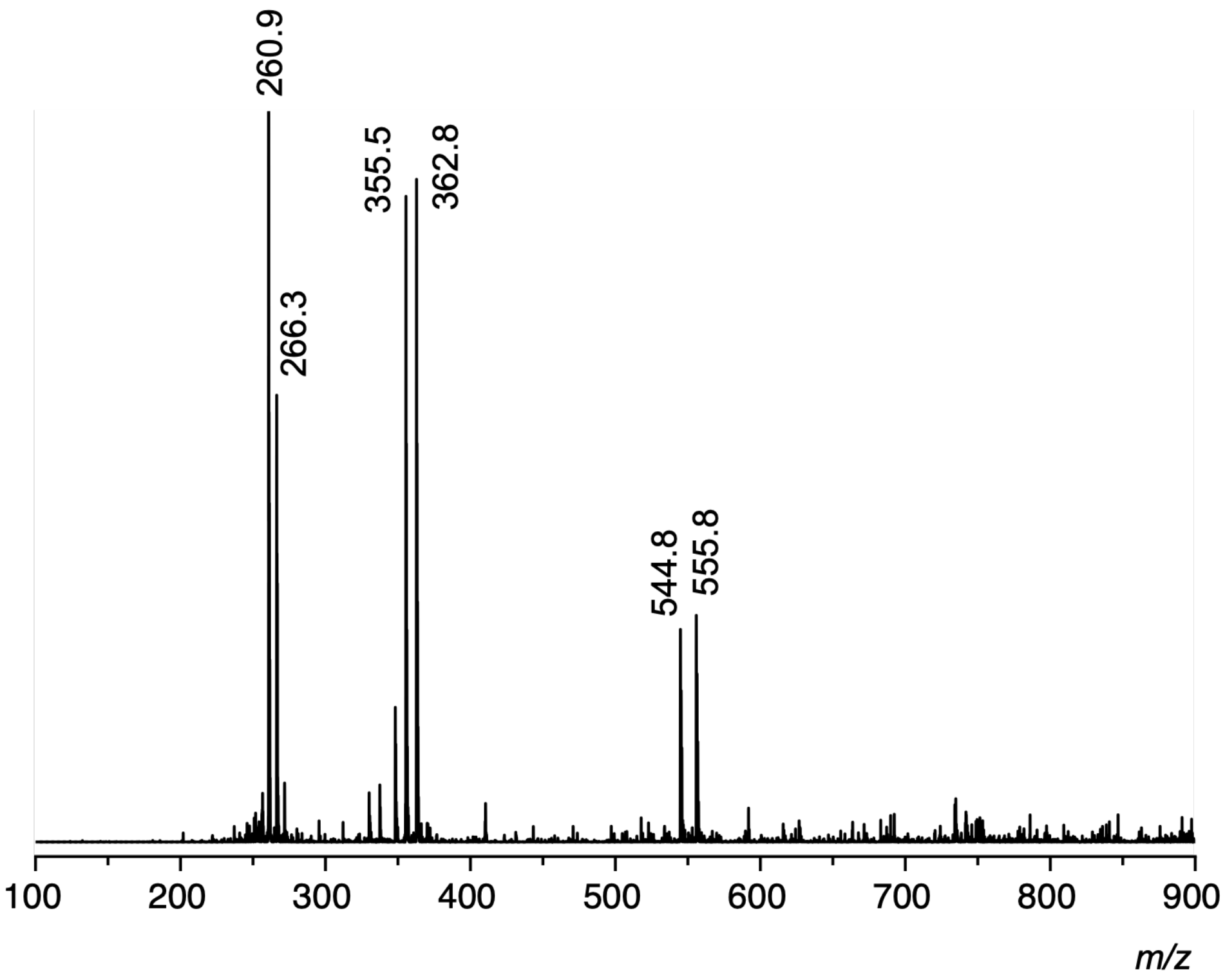

		m/z calcd.	*m/z exp.*	*Int.*
$[M–5Na+H]^{4–}$	$C_{52}H_{36}O_{16}S_4^{4–}$	261.0	260.9	100
$[M–4Na]^{4–}$	$C_{52}H_{35}NaO_{16}S_4^{4–}$	266.5	266.3	60
$[M–4Na+H]^{3–}$	$C_{52}H_{36}NaO_{16}S_4^{3–}$	355.7	355.5	86
$[M–3Na]^{3–}$	$C_{52}H_{35}Na_2O_{16}S_4^{3–}$	363.0	362.8	88
$[M–3Na+H]^{2–}$	$C_{52}H_{36}Na_2O_{16}S_4^{2–}$	545.0	544.8	26
$[M–2Na]^{2–}$	$C_{52}H_{35}Na_3O_{16}S_4^{2–}$	556.0	555.8	30

HPLC

152 NS486+TFA_neu2

	NS486+TFA_neu2		10,0
Vial Number:	BC12	*Channel:*	UV_VIS_3
Sample Type:	unknown	*Wavelength:*	280
Control Program:	PG 10-90 Flowpos1	*Bandwidth:*	1
Quantif. Method:	default	*Dilution Factor:*	1,0000
Recording Time:	9.6.2026 14:57	*Sample Weight:*	1,0000
Run Time (min):	41,00	*Sample Amount:*	1,0000

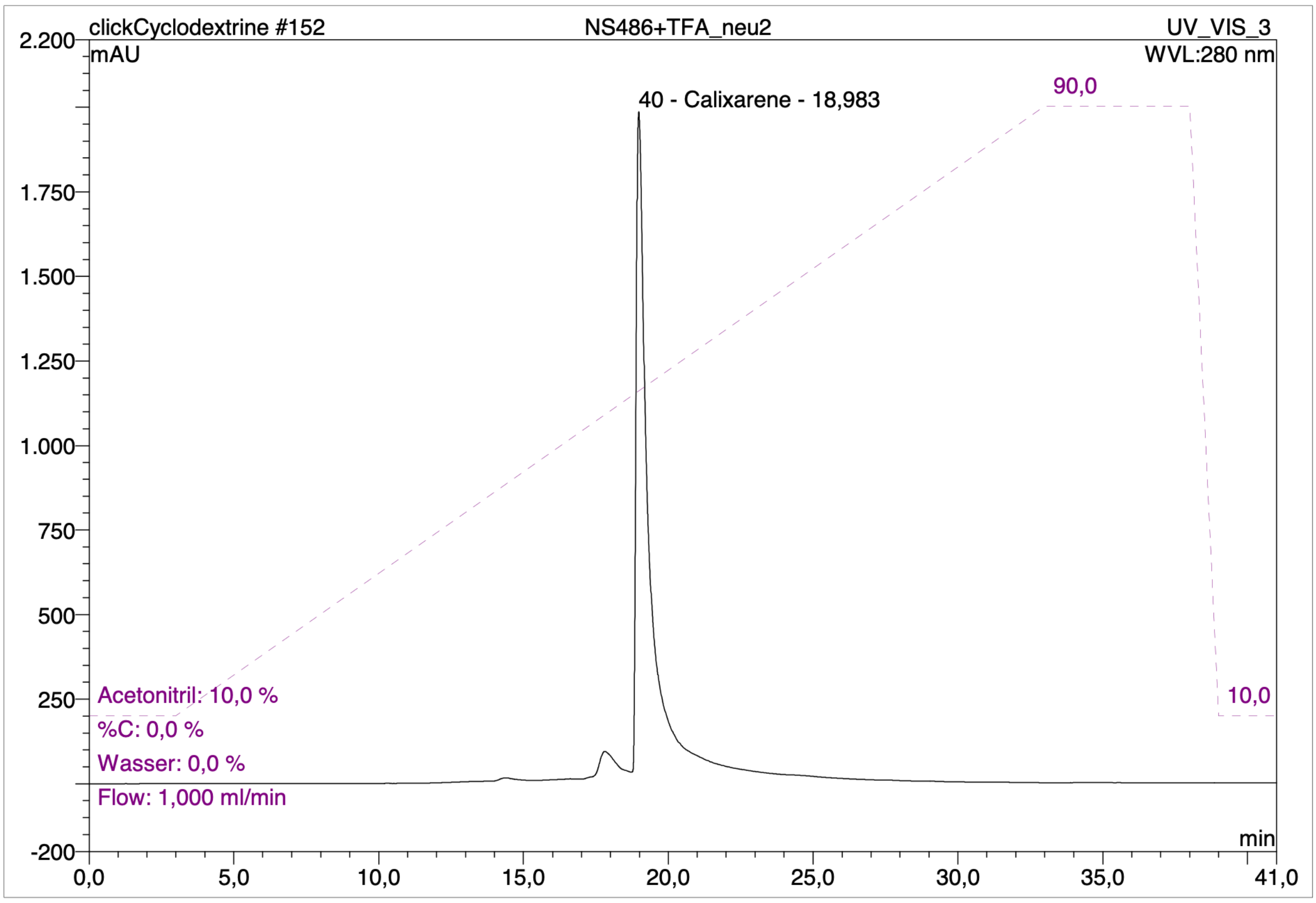

No.	Ret.Time min	Peak Name	mAU	Area mAU*min	Rel.Area %	Amount	
40	18,98	Calixarene	1985,136	1204,976	89,47	n.a.	M
**Total:**			1985,136	1204,976	89,47	0,000	

Binding Studies

NMR Spectroscopic Binding Studies. All measurements were performed in D_2O containing 1 vol% of CD_3OD as the internal standard. A stock solution of **1** (4 mM) was prepared. To an aliquot of this solution (500 µL), a defined volume of a liquid guest (10 molar equivalents, 20 µmol) was added. The resulting mixture was shaken on a laboratory shaker for 1 h at room temperature. Then, the ^1H NMR spectrum was measured without further treatment. For reference, the ^1H NMR spectra of the calixarene stock solution and of the aqueous alkane solutions prepared as described above, in the absence of the calixarene, were also measured. The volumes of the guests added were:

Guest	Formula	M.W. / g mol^{-1}	Density / g mL^{-1}	Volume / µL
n-Pentane	C_5H_{12}	72.15	0.626	2.3
n-Hexane	C_6H_{14}	86.18	0.661	2.6
n-Heptane	C_7H_{16}	100.21	0.684	2.9
n-Octane	C_8H_{18}	114.23	0.700	3.3
n-Nonane	C_9H_{20}	128.26	0.718	3.6
n-Decane	$C_{10}H_{22}$	142.29	0.730	3.9
Cyclopentane	C_5H_{10}	70.13	0.751	1.9
Cyclohexane	C_6H_{12}	84.16	0.779	2.2
Cycloheptane	C_7H_{14}	98.19	0.811	2.4
Cyclooctane	C_8H_{16}	112.22	0.834	2.7
Chloroform	$CHCl_3$	119.37	1.489	1.6
Hexafluorobenzene	C_6F_6	186.06	1.612	2.3
Perfluoro-*n*-hexane	C_6F_{14}	338.04	1.680	4.0
Benzene	C_6H_6	78.11	0.877	1.8

For the sample containing 0.5 equiv of benzene, a benzene stock solution (2 mM) in 1 vol% CH_3OD in D_2O was prepared. An aliquot of this solution was added to a known amount of **1** so that the concentration of the calixarene in the resulting solution was 4 mM. An aliquot (500 µL) of this solution was transferred to an NMR tube and the ^1H NMR spectrum was recorded. The NMR samples containing 1.0, 1.5, 2.0, 2.5, and 3.0 equiv of benzene were prepared analogously using 4 mM, 6 mM, 8 mM, 10 mM, and 12 mM benzene stock solutions. The 8 mM benzene stock solution was used to determine the chemical shift of the benzene protons in the absence of **1**.

For DOSY experiments, NMR spectra were recorded in D_2O with 1 vol% CD_3OD on a Bruker Avance III spectrometer operating at 600 MHz and equipped with a 5 mm BBO probe at 300 K. The standard stimulated echo bipolar gradient pulse sequence from the Bruker library was used with 100 ms big delta and running gradient ramp of 64 steps between 5 and 95% of maximum gradient strength of 2000 μs length. For the determination of the hydrodynamic radius of the cyclooctane complex of **1**, the generation 6 PAMAM-OH dendrimer was added as calibration reference. Within the same sample tube, temperature and viscosity are identical for the components and the Stokes-Einstein equation then allows to relate the hydrodynamic radii of two components via the measured diffusion coefficients of the capsule and the dendrimer: D(capsule) × R_h(capsule) = D(dendrimer) × R_h(dendrimer), with the G6-PAMAM-OH dendrimer diameter being 7.0 nm.[6]

^{1}H NMR Spectroscopic Investigation of Benzene Binding

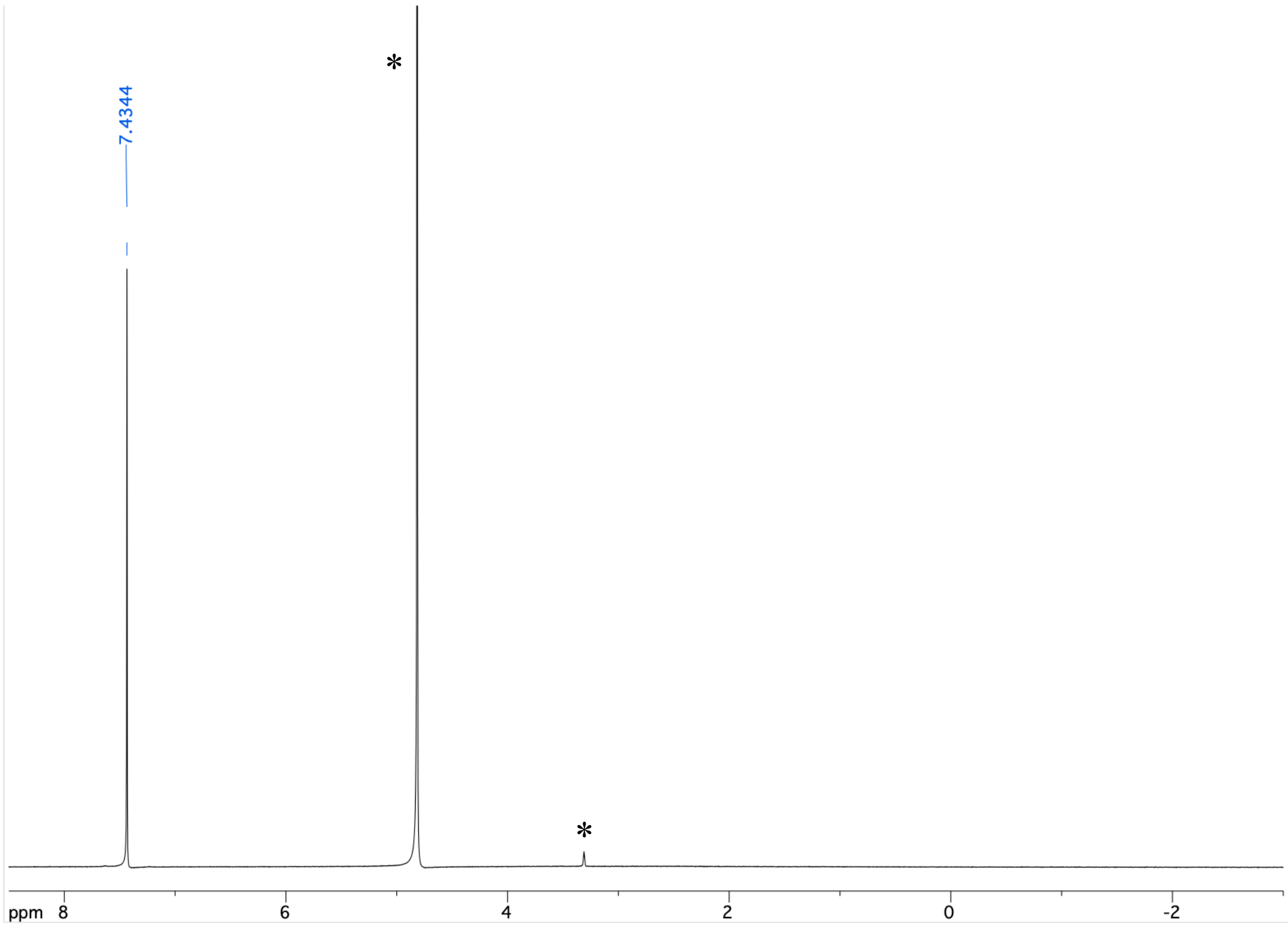

Figure S1: ^{1}H NMR spectrum of benzene in 1 vol% CD_3OD in D_2O (T = 298 K). The solvent signals are marked with asterisks.

(a)

(b)

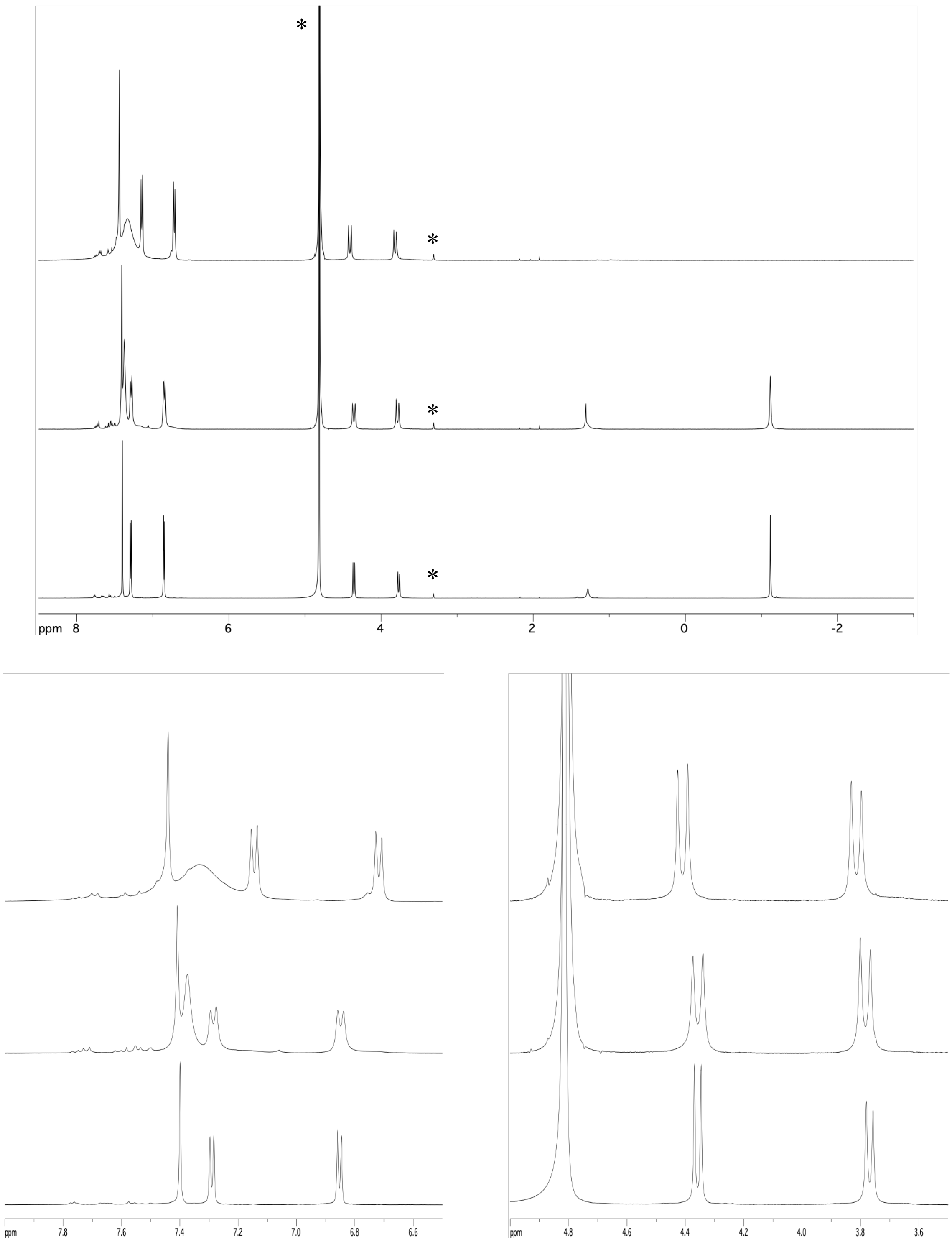

Figure S2: ^{1}H NMR spectra of **1** in 1 vol% CD_3OD in D_2O (4 mM) in the presence of 10 molar equivalents of benzene (top), 10 molar equivalents of cyclohexane (bottom), and 10 molar equivalents of benzene followed by 10 molar equivalents of cyclohexane (middle) (T = 298 K) (a). The spectra in (b) show enlarged regions of the spectra in (a).

(a)

(b)

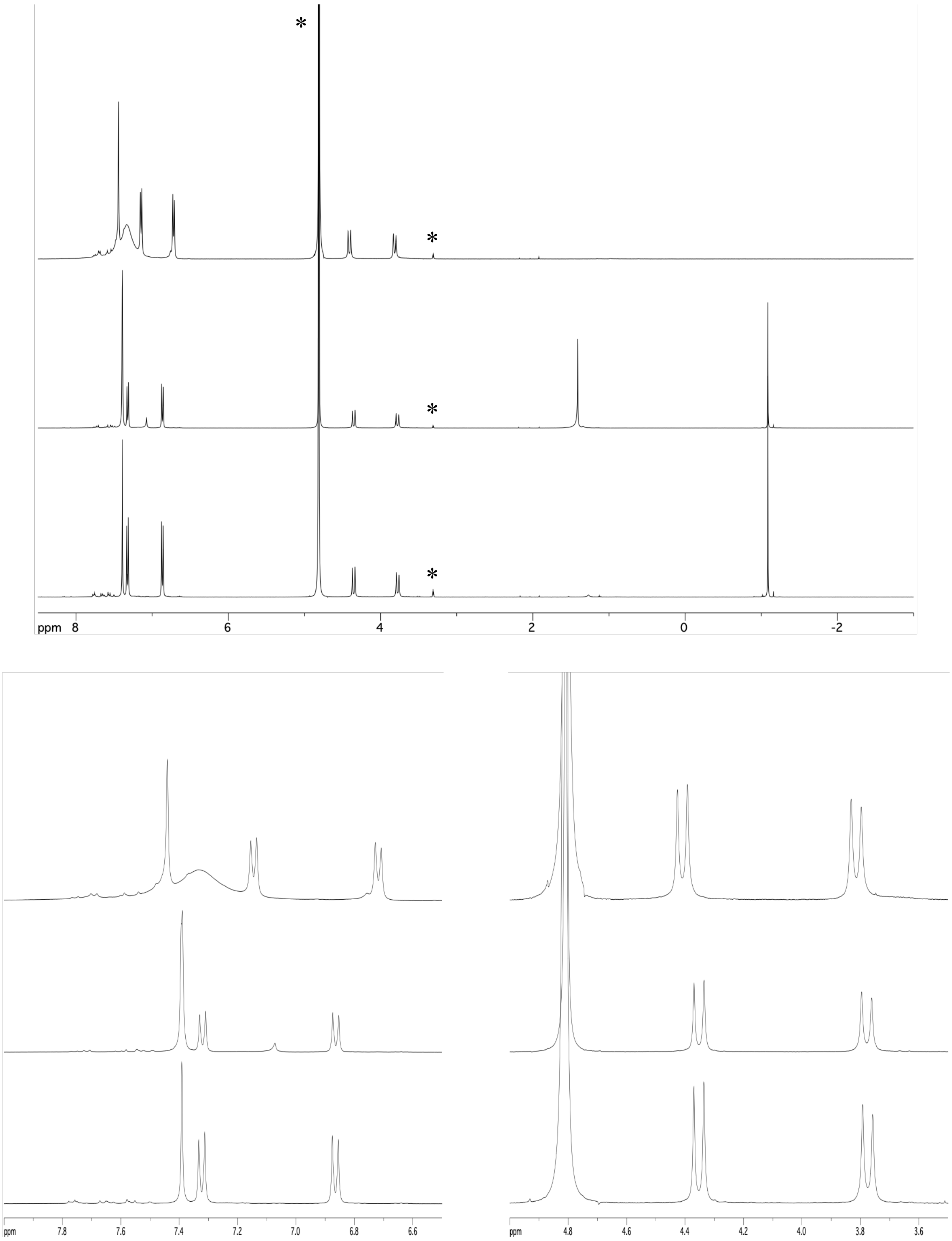

Figure S3: ^{1}H NMR spectra of **1** in 1 vol% CD_3OD in D_2O (4 mM) in the presence of 10 molar equivalents of benzene (top), 10 molar equivalents of cycloheptane (bottom), and 10 molar equivalents of benzene followed by 10 molar equivalents of cycloheptane (middle) (T = 298 K) (a). The spectra in (b) show enlarged regions of the spectra in (a).

^{1}H NMR Spectroscopic Investigation of Cycloalkane Binding

(a)

(b)

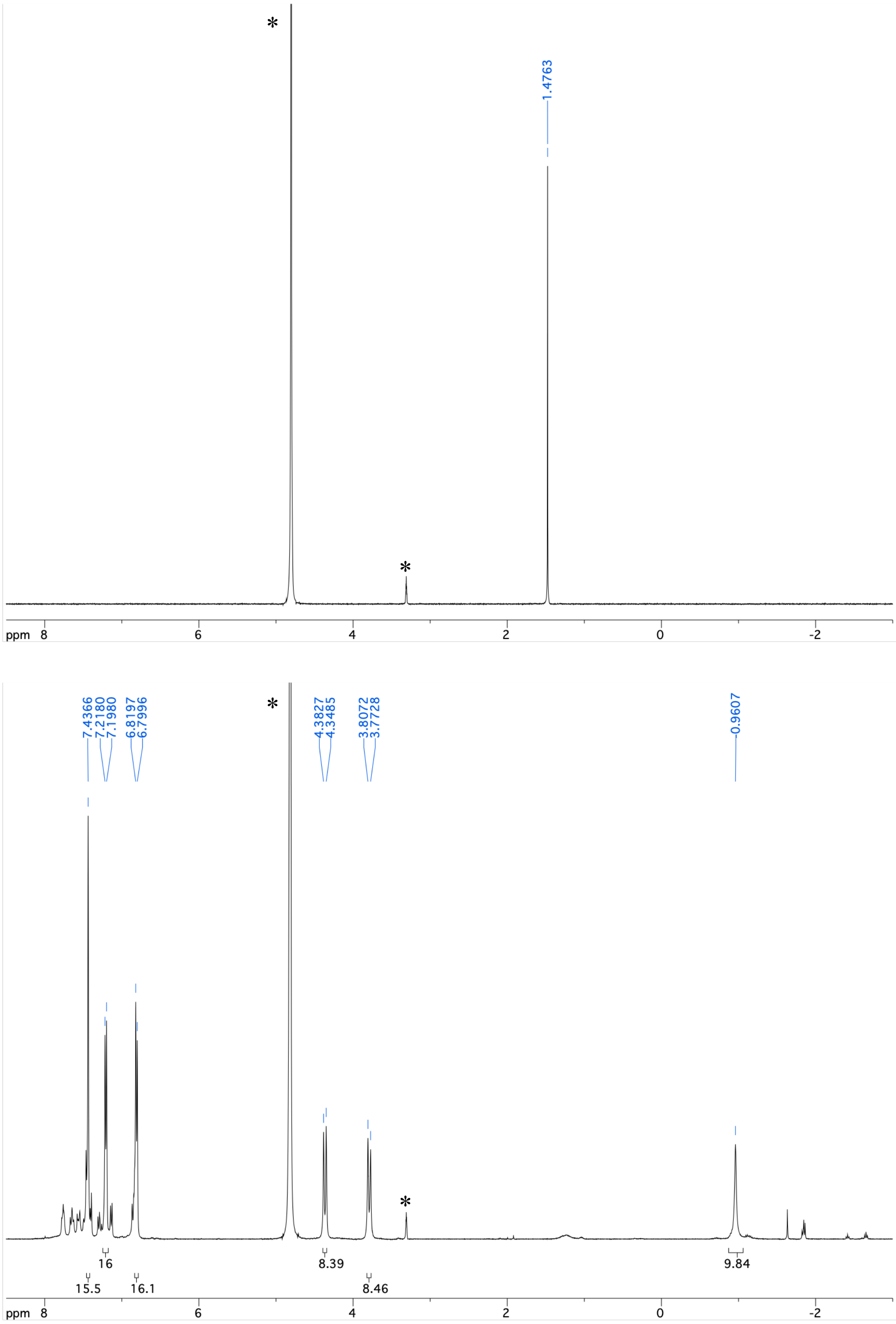

Figure S4: ^{1}H NMR spectra of cyclopentane in 1 vol% CD_3OD in D_2O (a) and of **1** (4 mM) treated with 10 molar equivalents of cyclopentane in the same solvent mixture (T = 298 K). The solvent signals are marked with asterisks.

(a)

(b)

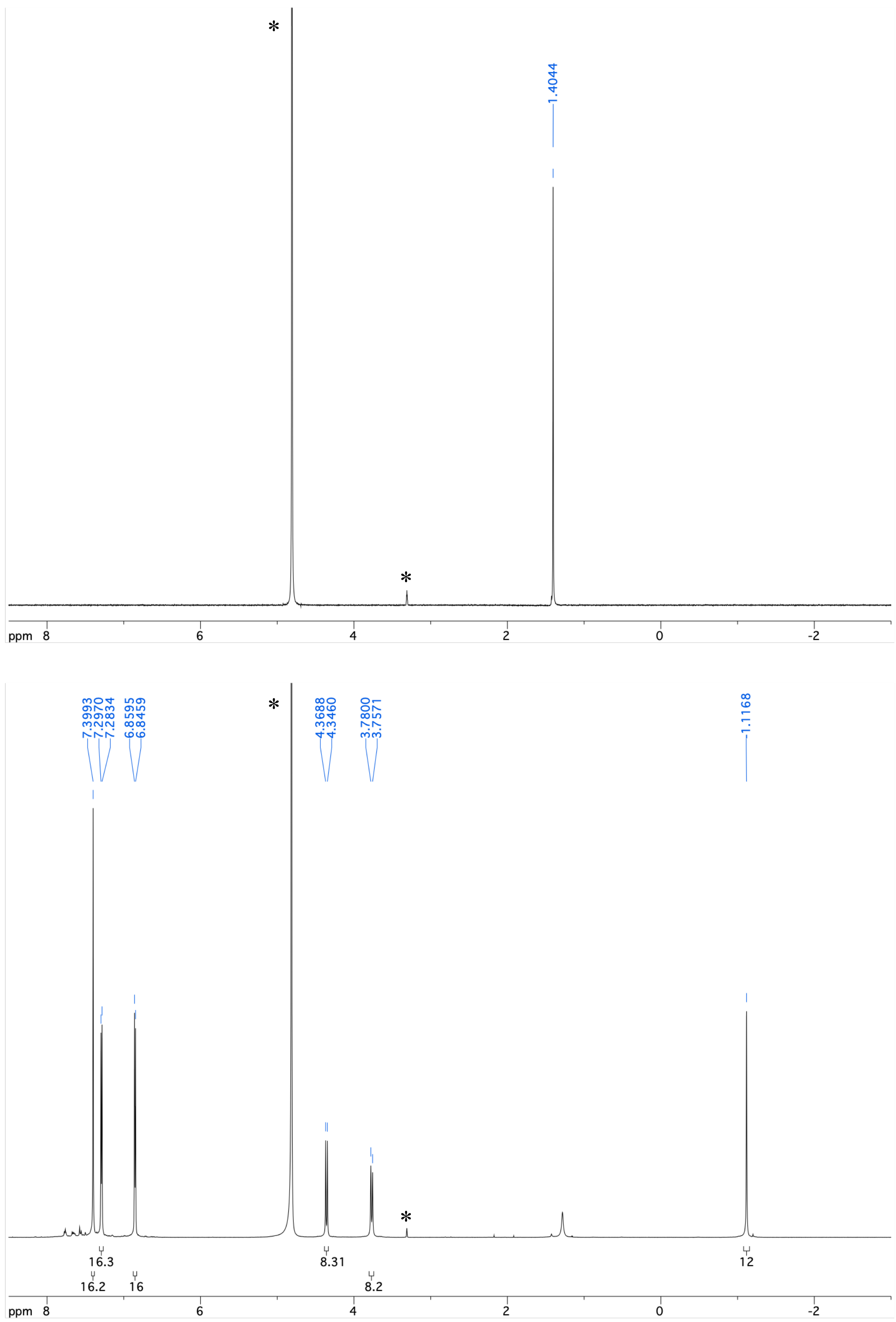

Figure S5: ^{1}H NMR spectra of cyclohexane in 1 vol% CD_3OD in D_2O (a) and of **1** (4 mM) treated with 10 molar equivalents of cyclohexane in the same solvent mixture (T = 298 K). The solvent signals are marked with asterisks.

(a)

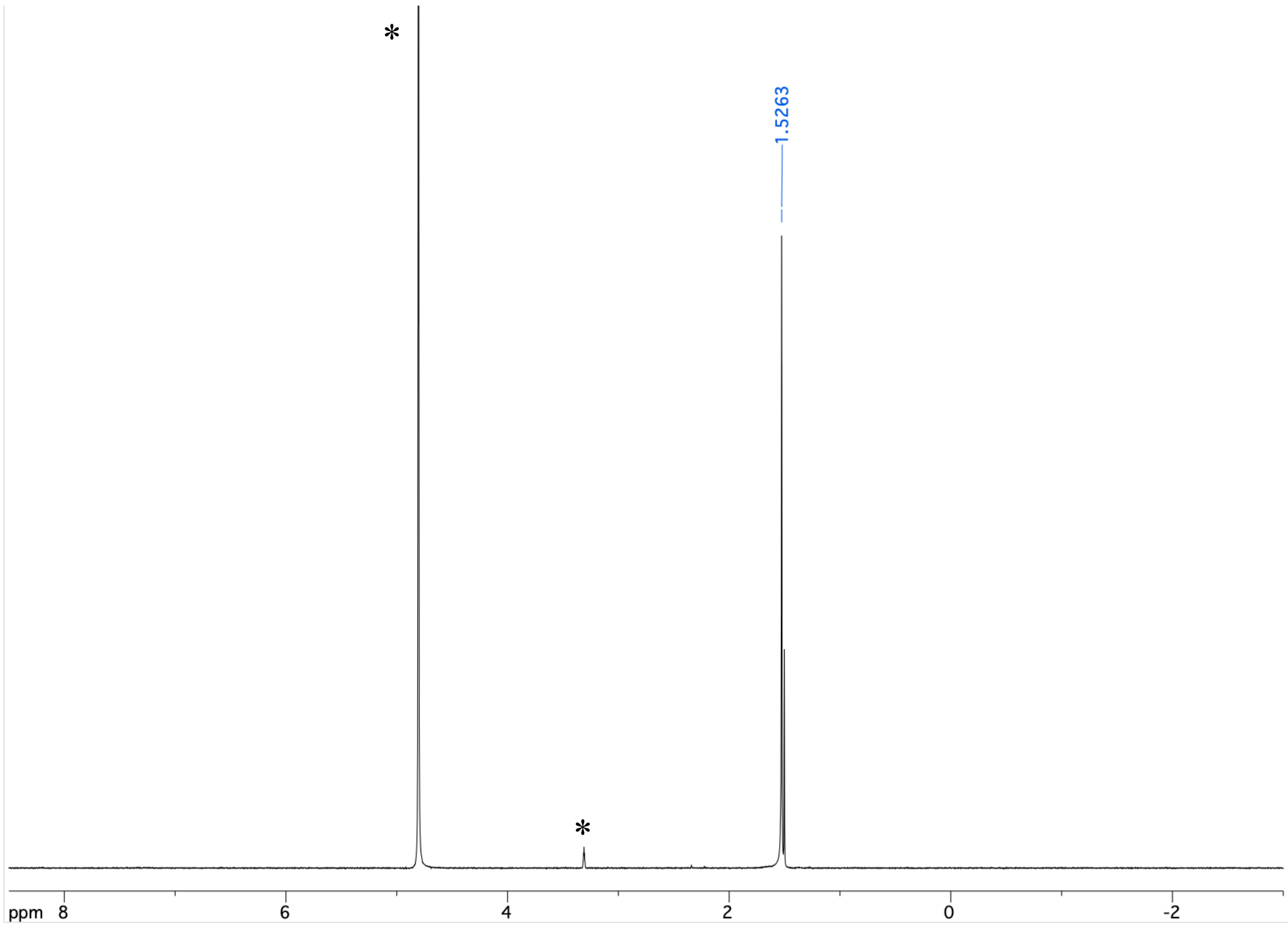

(b)

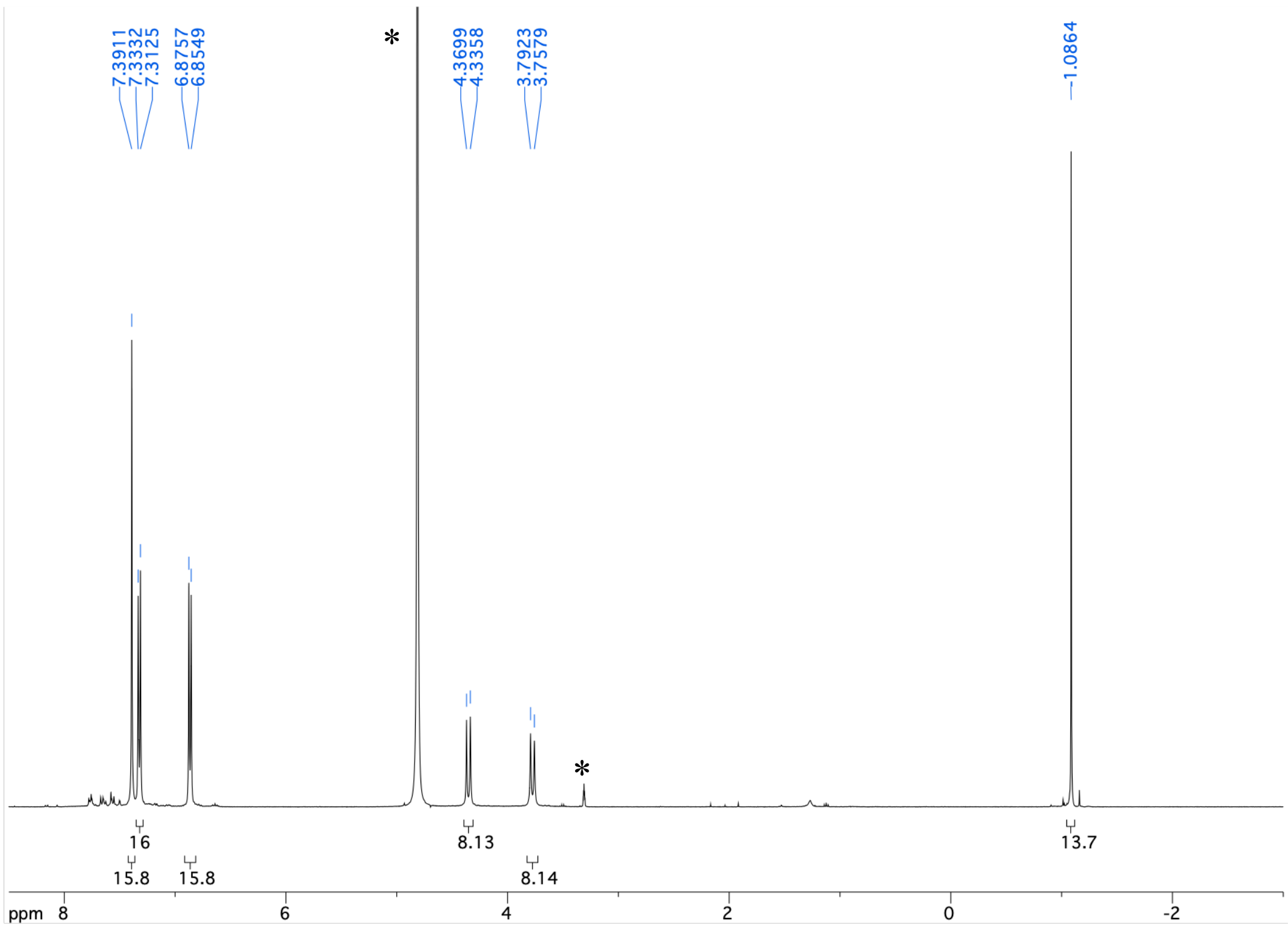

Figure S6: ^{1}H NMR spectra of cycloheptane in 1 vol% CD_3OD in D_2O (a) and of **1** (4 mM) treated with 10 molar equivalents of cycloheptane in the same solvent mixture (T = 298 K). The solvent signals are marked with asterisks.

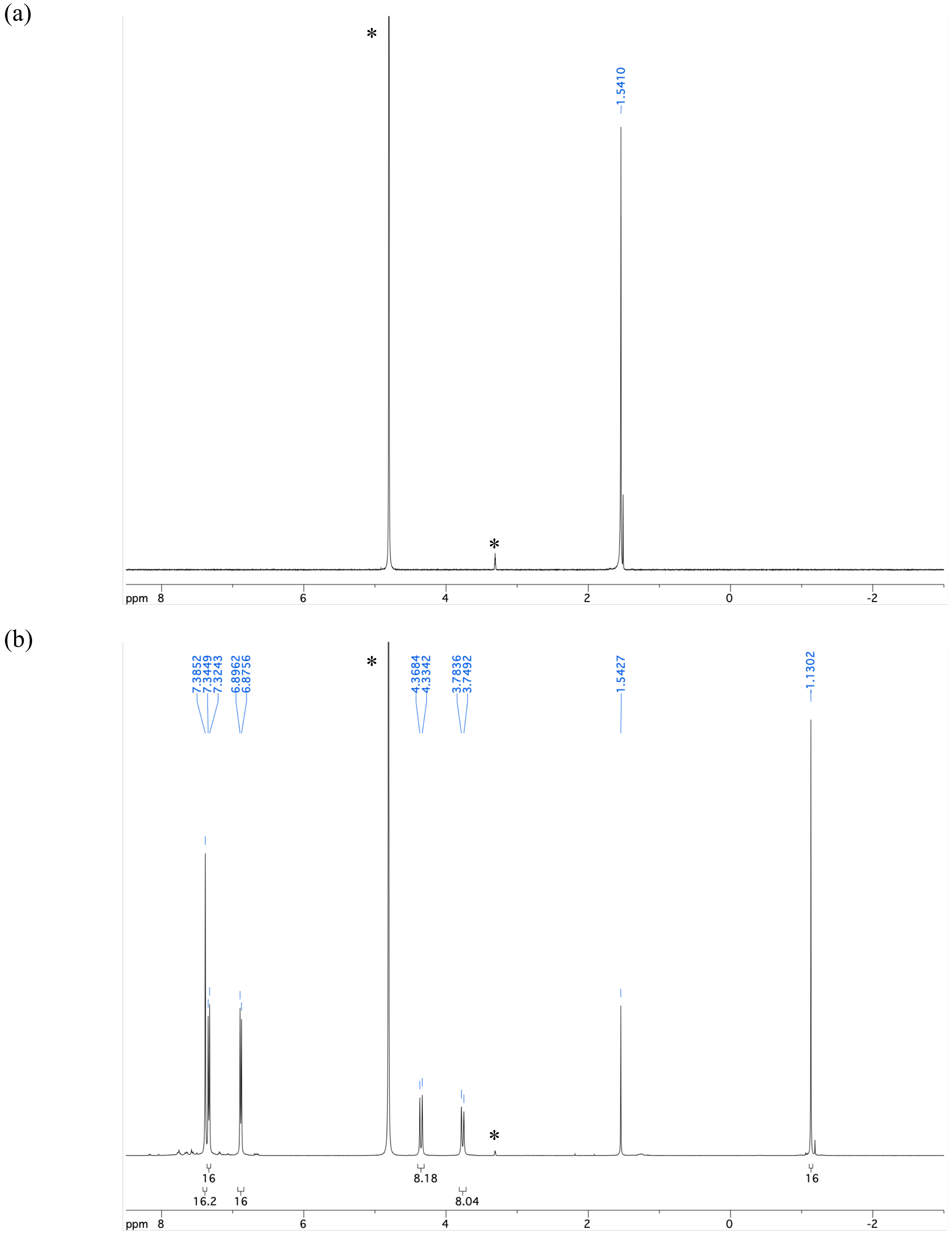

Figure S7: ^{1}H NMR spectra of cyclooctane in 1 vol% CD_3OD in D_2O (a) and of **1** (4 mM) treated with 10 molar equivalents of cyclooctane in the same solvent mixture ($T = 298$ K). The solvent signals are marked with asterisks.

ROESY Spectra of Cycloalkane Complexes

(a)

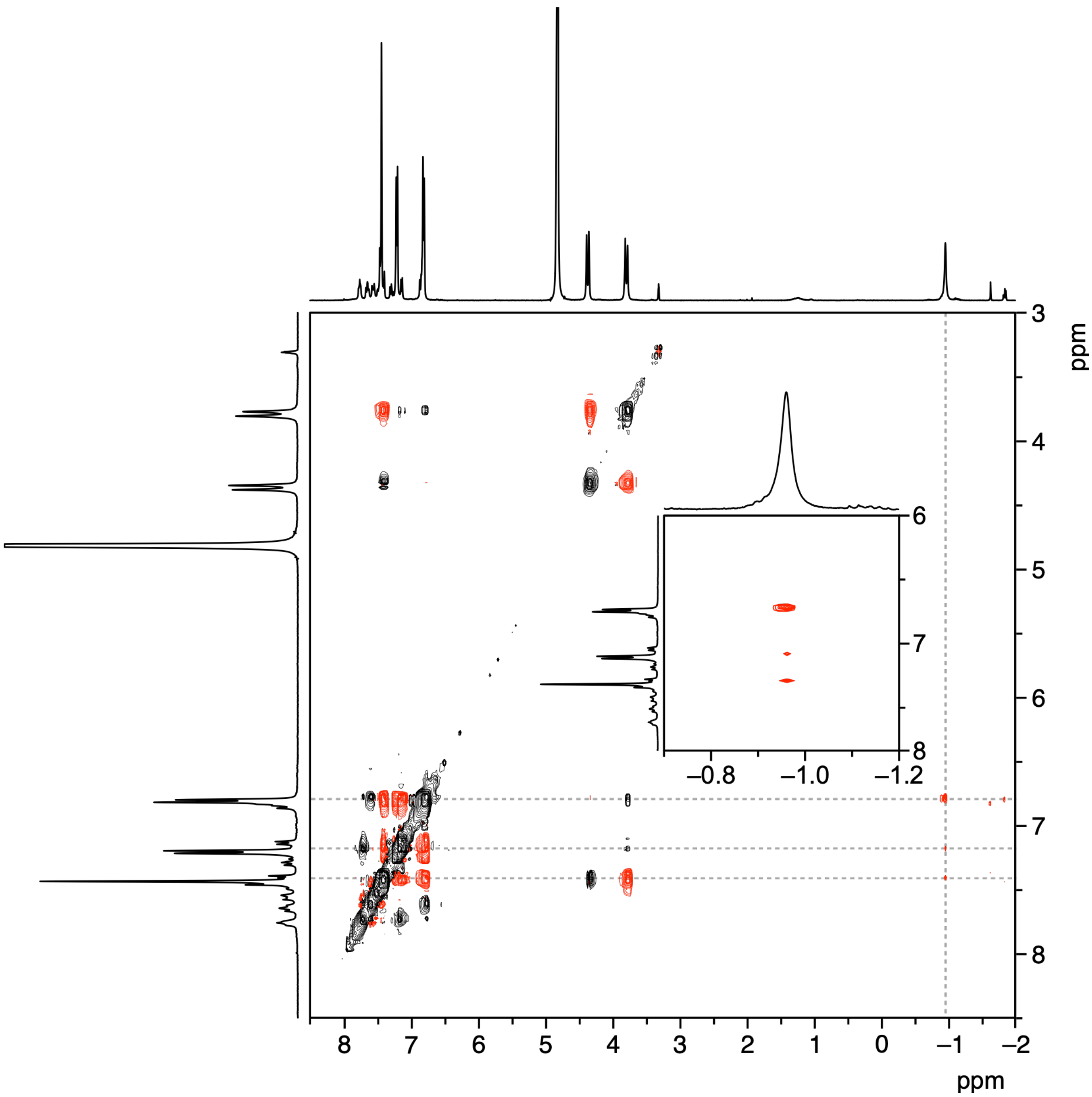

(b)

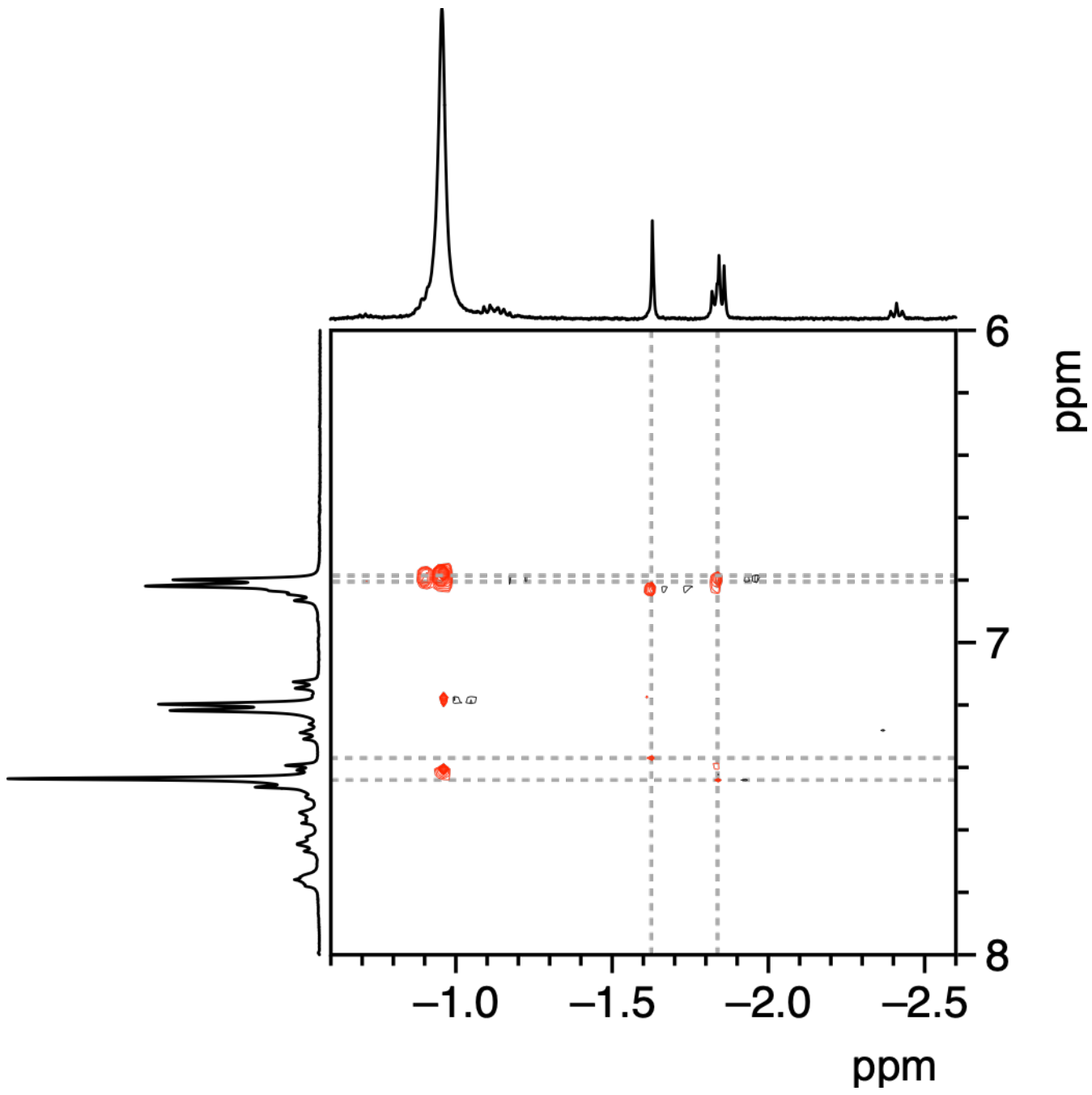

Figure S8: (a) ROESY spectrum of **1** (4 mM) treated with 10 molar equivalents of cyclopentane in 1 vol% CD_3OD in D_2O (T = 298 K). Mixing time 500 ms. The inset in (a) shows the cross peaks between the signal of the bound guest and the aromatic signals of **1**. The section of the spectrum in (b) shows the cross peaks between guest and host signals of the minor complex species.

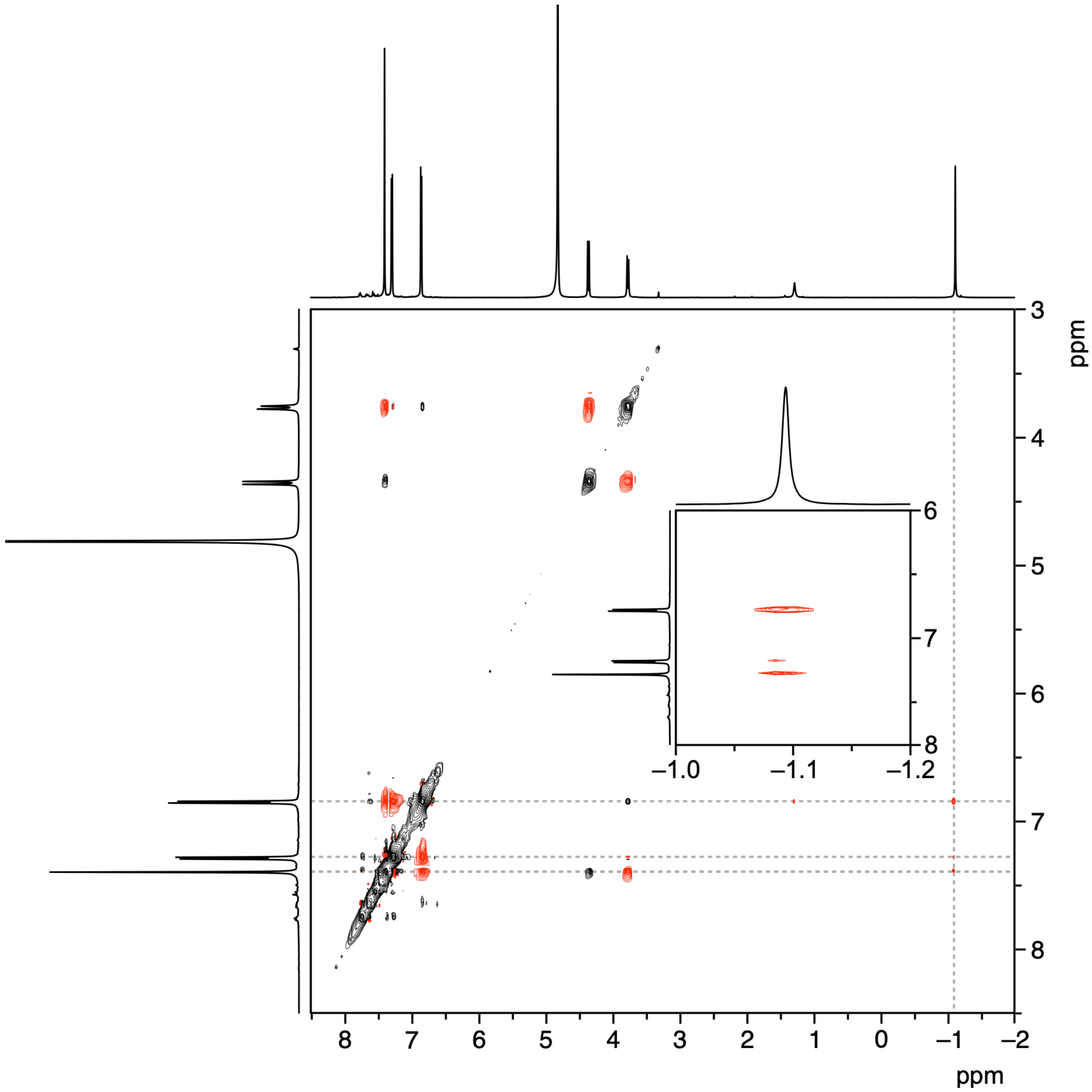

Figure S9: ROESY spectrum of **1** (4 mM) treated with 10 molar equivalents of cyclohexane in 1 vol% CD_3OD in D_2O ($T = 298$ K). Mixing time 500 ms. The inset shows the cross peaks between the signal of the bound guest and the aromatic signals of **1**.

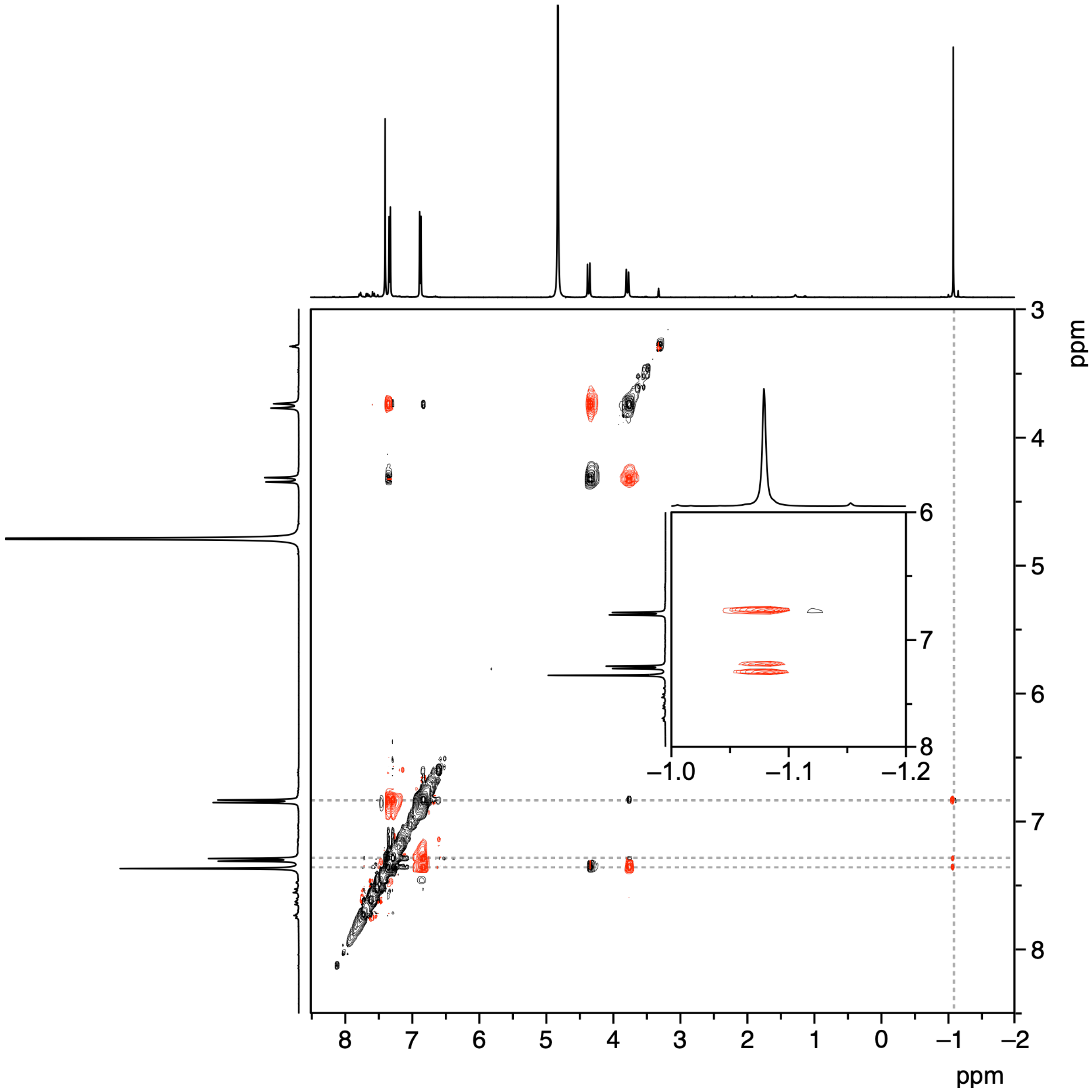

Figure S10: ROESY spectrum of **1** (4 mM) treated with 10 molar equivalents of cycloheptane in 1 vol% CD_3OD in D_2O ($T = 298$ K). Mixing time 500 ms. The inset shows the cross peaks between the signal of the bound guest and the aromatic signals of **1**.

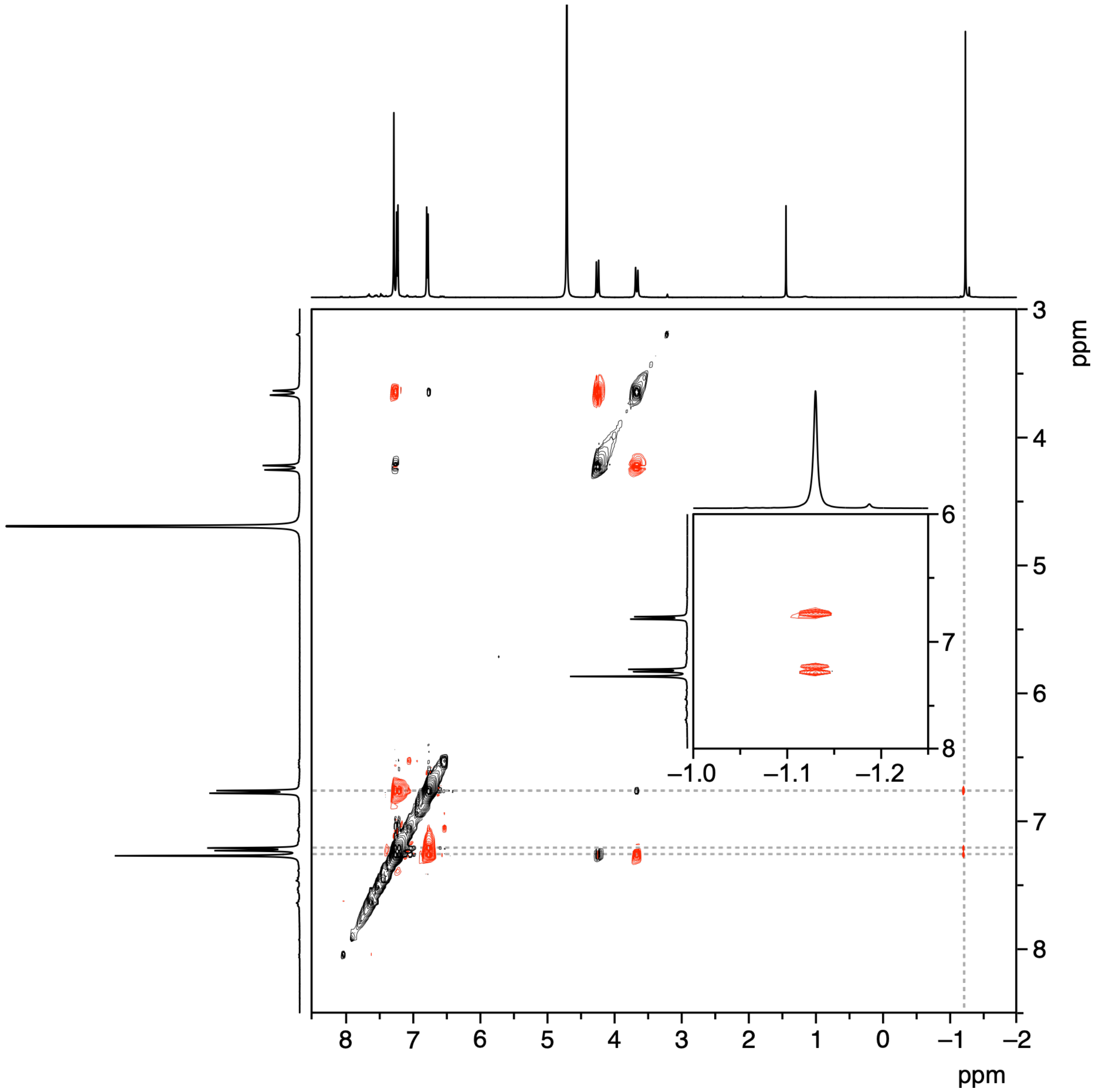

Figure S11: ROESY spectrum of **1** (4 mM) treated with 10 molar equivalents of cyclooctane in 1 vol% CD_3OD in D_2O ($T = 298$ K). Mixing time 500 ms. The inset shows the cross peaks between the signal of the bound guest and the aromatic signals of **1**.

DOSY Spectra of Cycloalkane Complexes

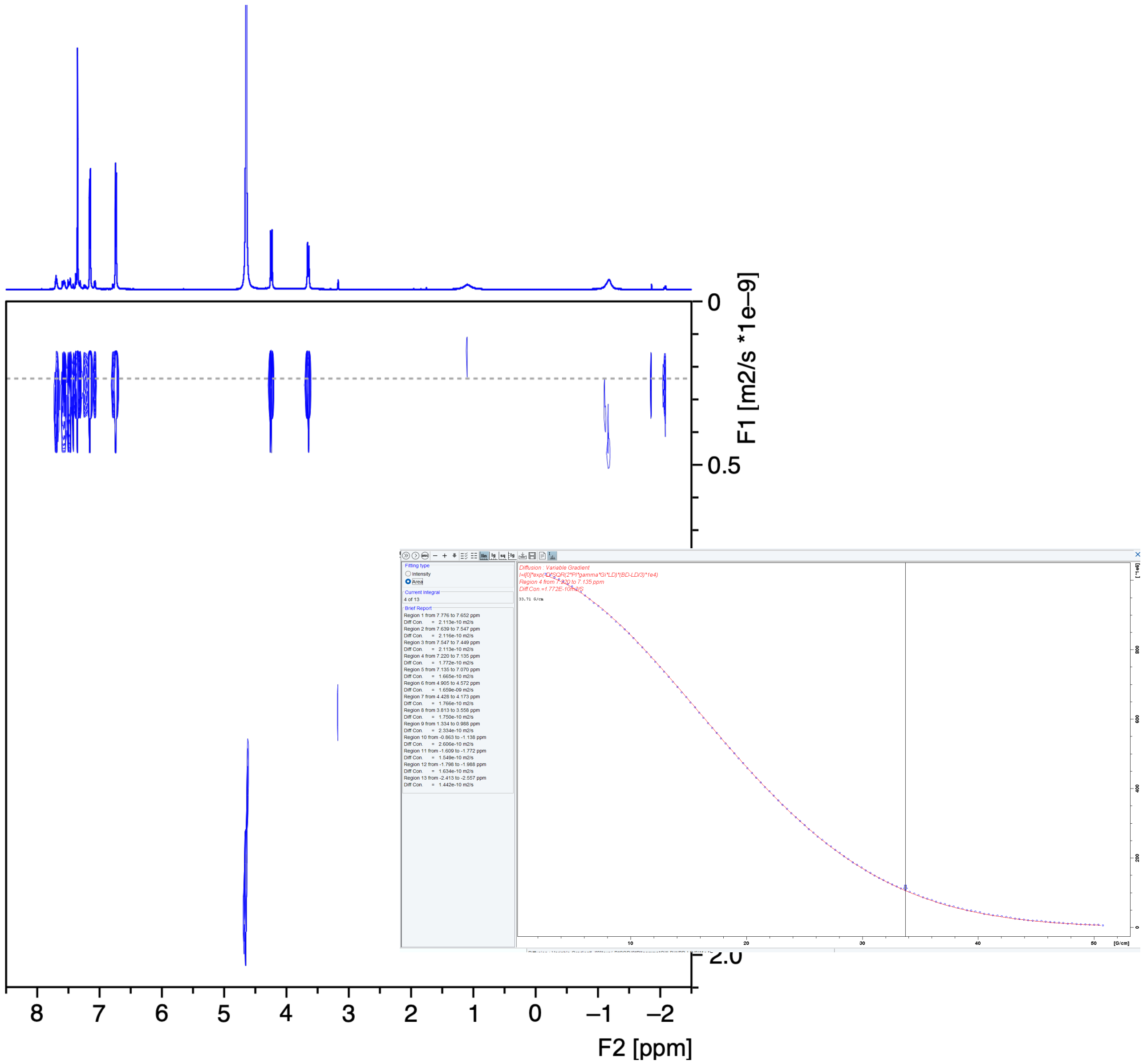

Figure S12: DOSY spectrum of **1** (4 mM) treated with 10 molar equivalents of cyclopentane in 1 vol% CD_3OD in D_2O ($T = 300$ K). The graph shows the non-normalized fitted D values for a selection of peaks.

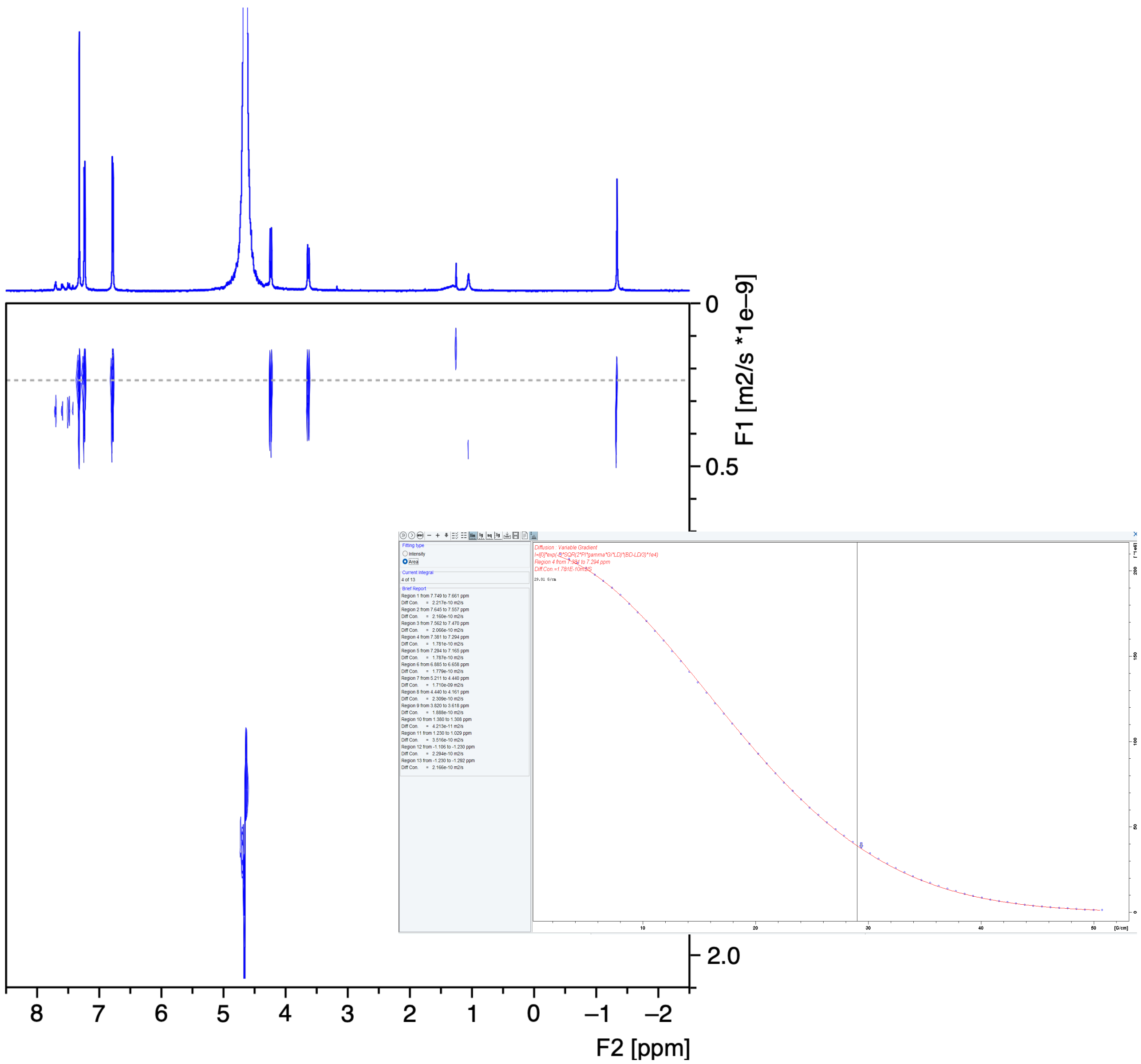

Figure S13: DOSY spectrum of **1** (4 mM) treated with 10 molar equivalents of cyclohexane in 1 vol% CD_3OD in D_2O (T = 300 K). The graph shows the non-normalized fitted D values for a selection of peaks.

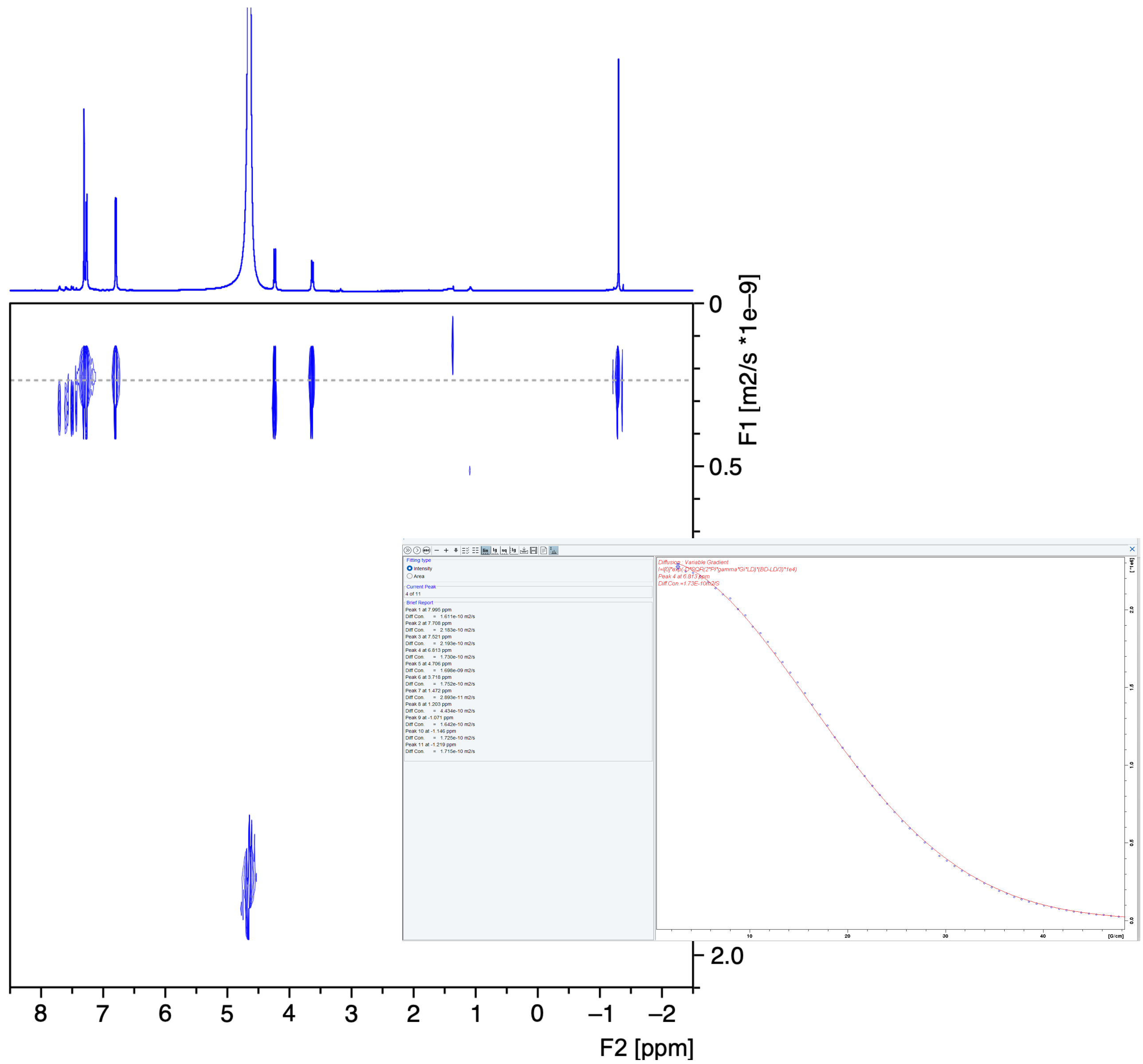

Figure S14: DOSY spectrum of **1** (4 mM) treated with 10 molar equivalents of cycloheptane in 1 vol% CD_3OD in D_2O (T = 300 K). The graph shows the non-normalized fitted D values for a selection of peaks.

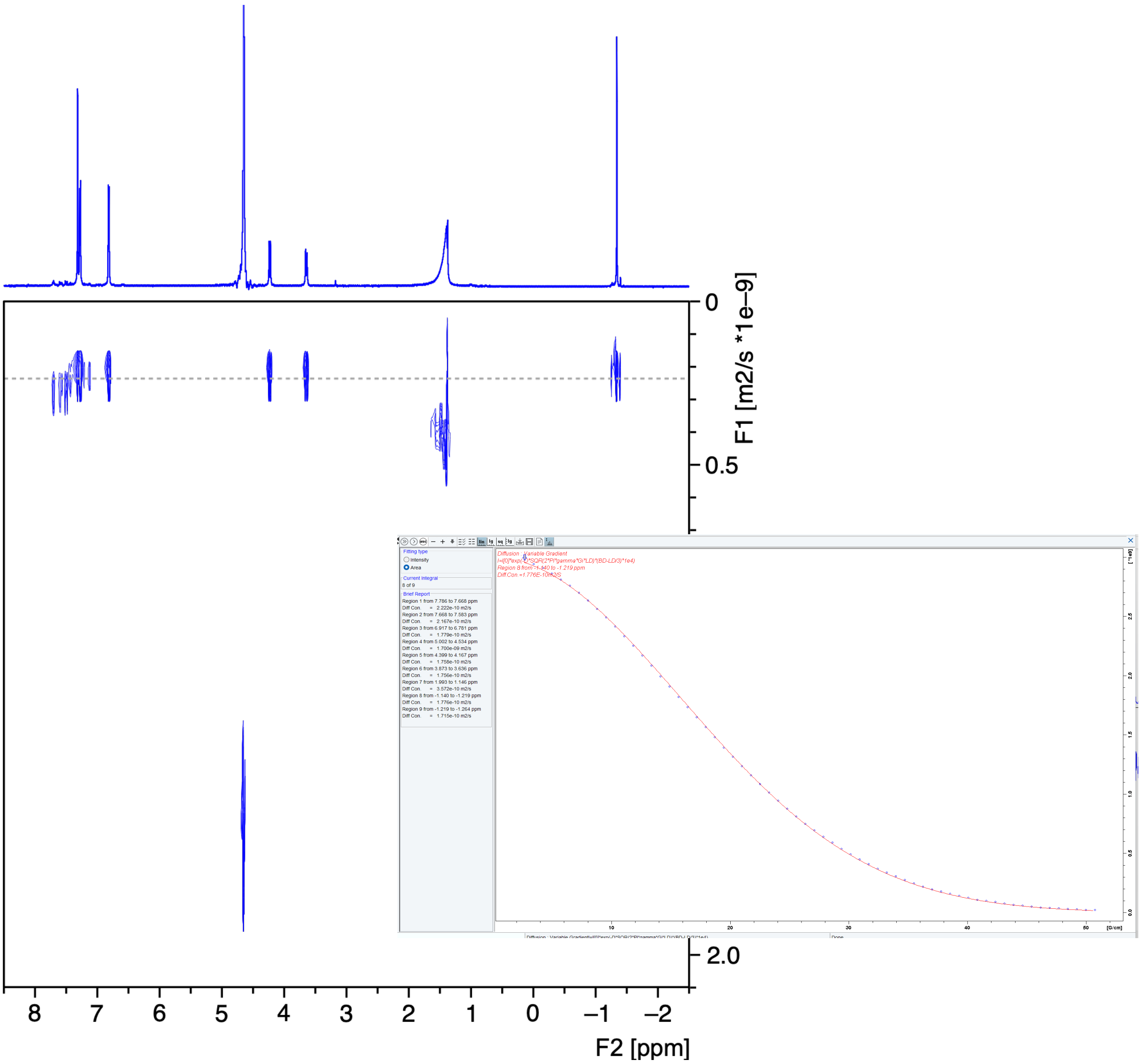

Figure S15: DOSY spectrum of **1** (4 mM) treated with 10 molar equivalents of cyclooctane in 1 vol% CD_3OD in D_2O (T = 300 K). The graph shows the non-normalized fitted D values for a selection of peaks.

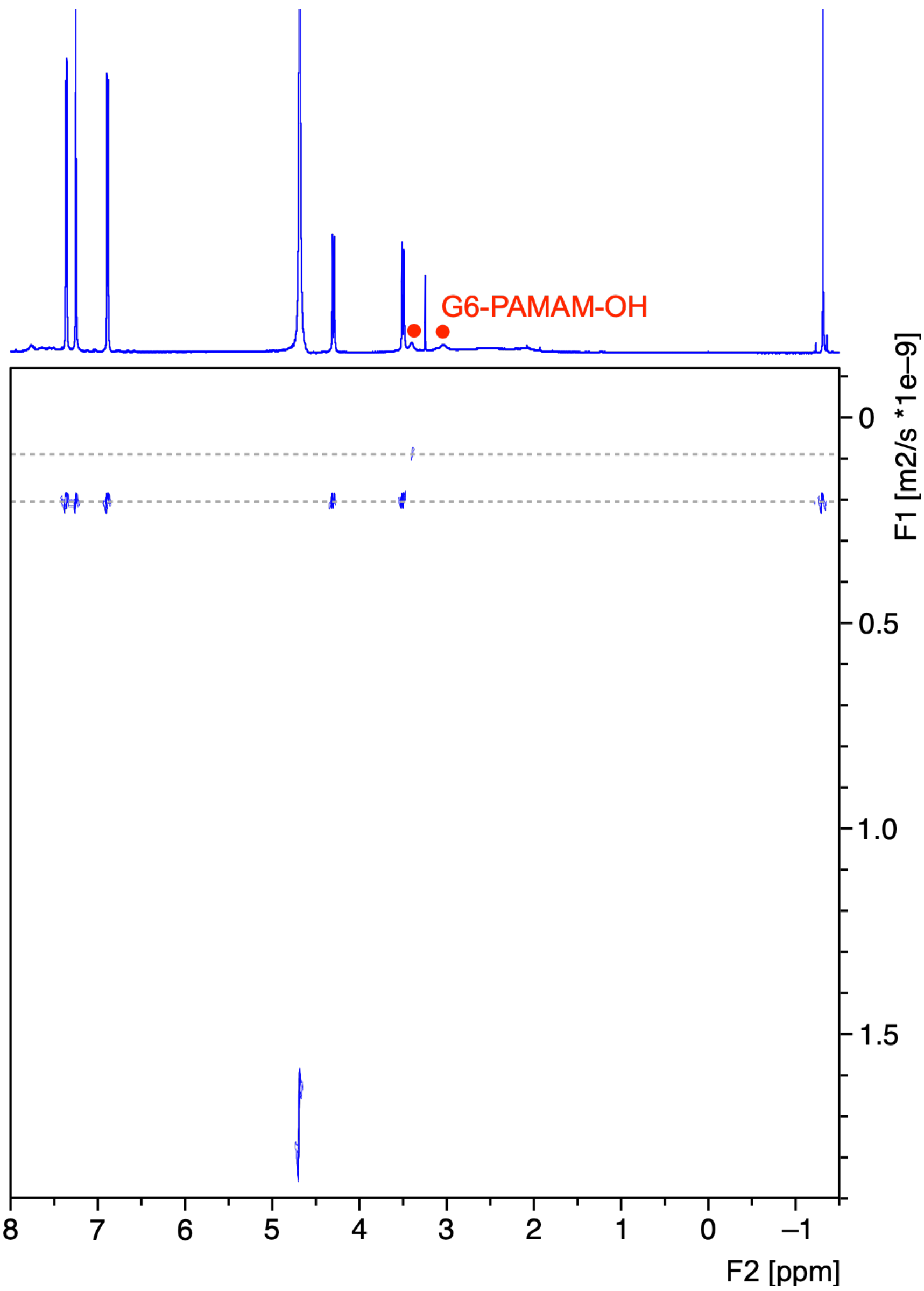

Figure S16: DOSY spectrum of **1** (4 mM) treated with 10 molar equivalents of cyclooctane in 1 vol% CD_3OD in D_2O in the presence of generation G6-PAMAM-OH (T = 300 K).

The diffusion coefficients as measured for the capsule and dendrimer signals at 3.54 and 3.44 ppm, respectively, were 1.94 and 0.89 × 10^{-10} m^2/s. Following from the Stokes-Einstein equation, the ratio of the respective diffusion coefficients, *D*(capsule)/*D*(dendrimer), is inversely proportional to the corresponding hydrodynamic radii of the two components, R_h(dendrimer)/R_h(capsule). Taking 7.0 nm for the diameter of the G6-PAMAM-OH results in a hydrodynamic diameter of the dimeric capsule of 3.2 nm.

^{1}H NMR Spectra of Competition Experiments with Cycloalkanes

(a)

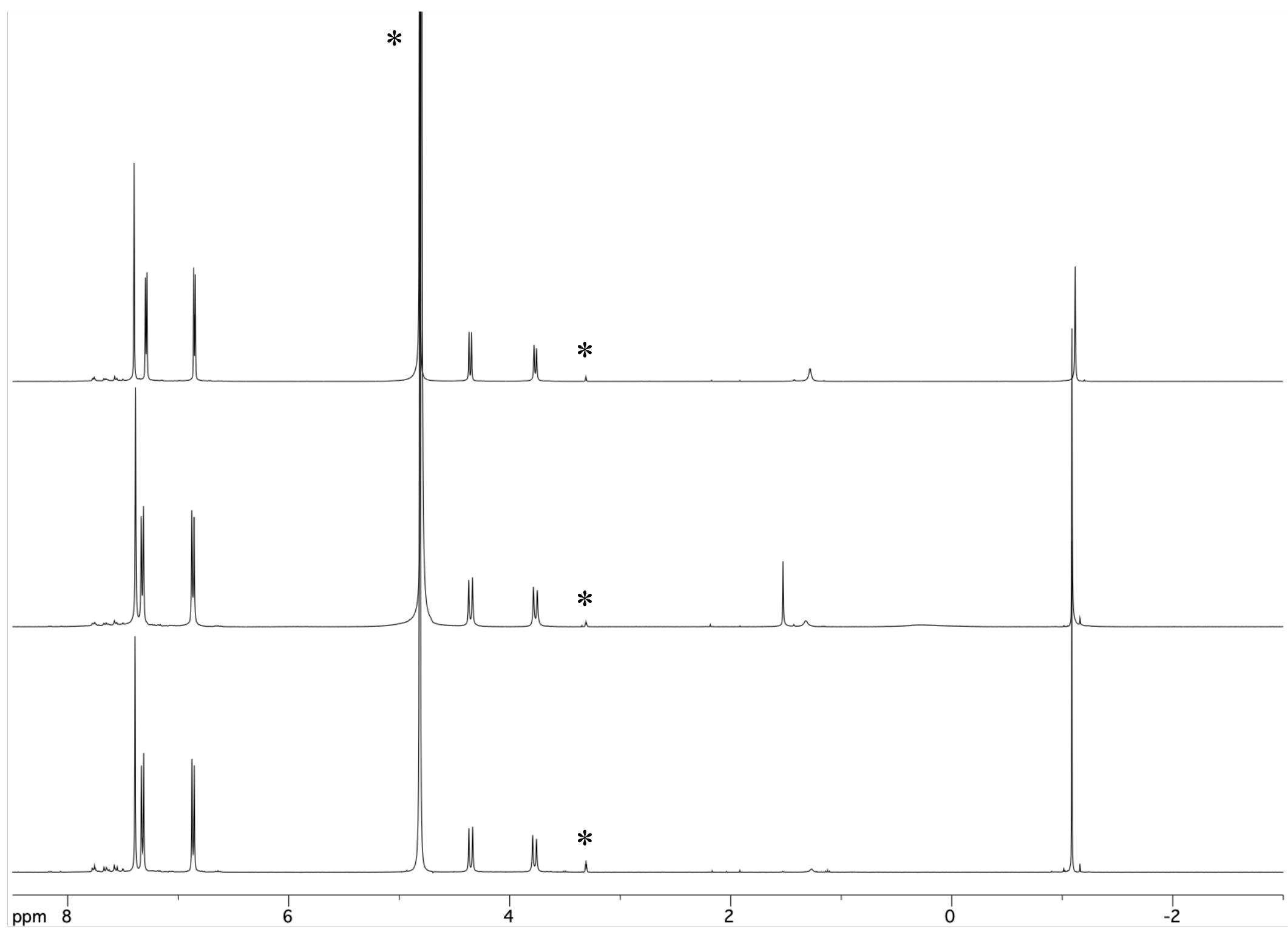

(b)

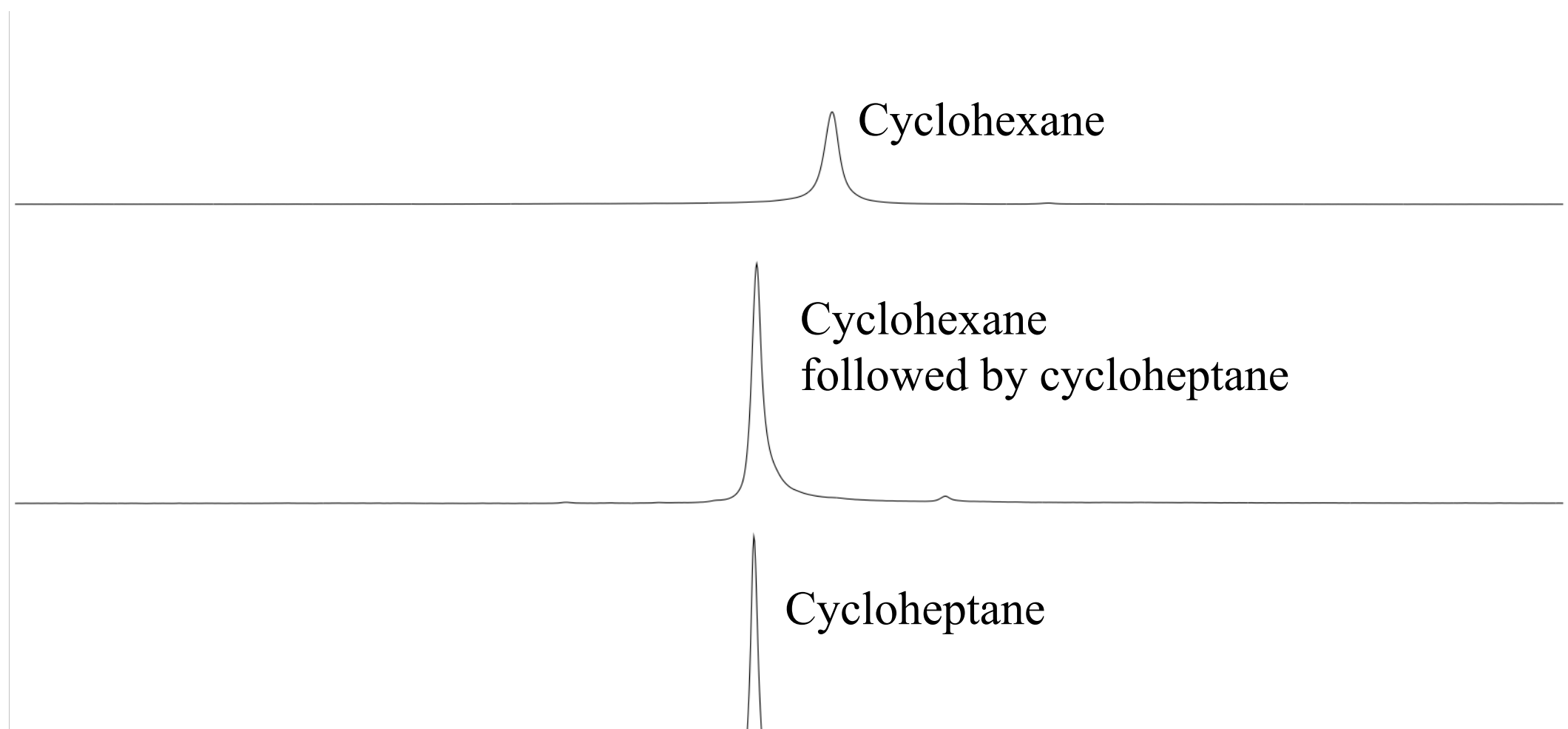

Figure S17: ^{1}H NMR spectra of **1** in 1 vol% CD_3OD in D_2O (4 mM) treated with 10 molar equivalents of cyclohexane (top), cycloheptane (bottom), and cyclohexane followed by the addition of 10 molar equivalents of cycloheptane (middle) (T = 298 K) (a). The spectra in (b) show the enlarged region of the spectra containing the signals of the bound guest.

(a)

(b)

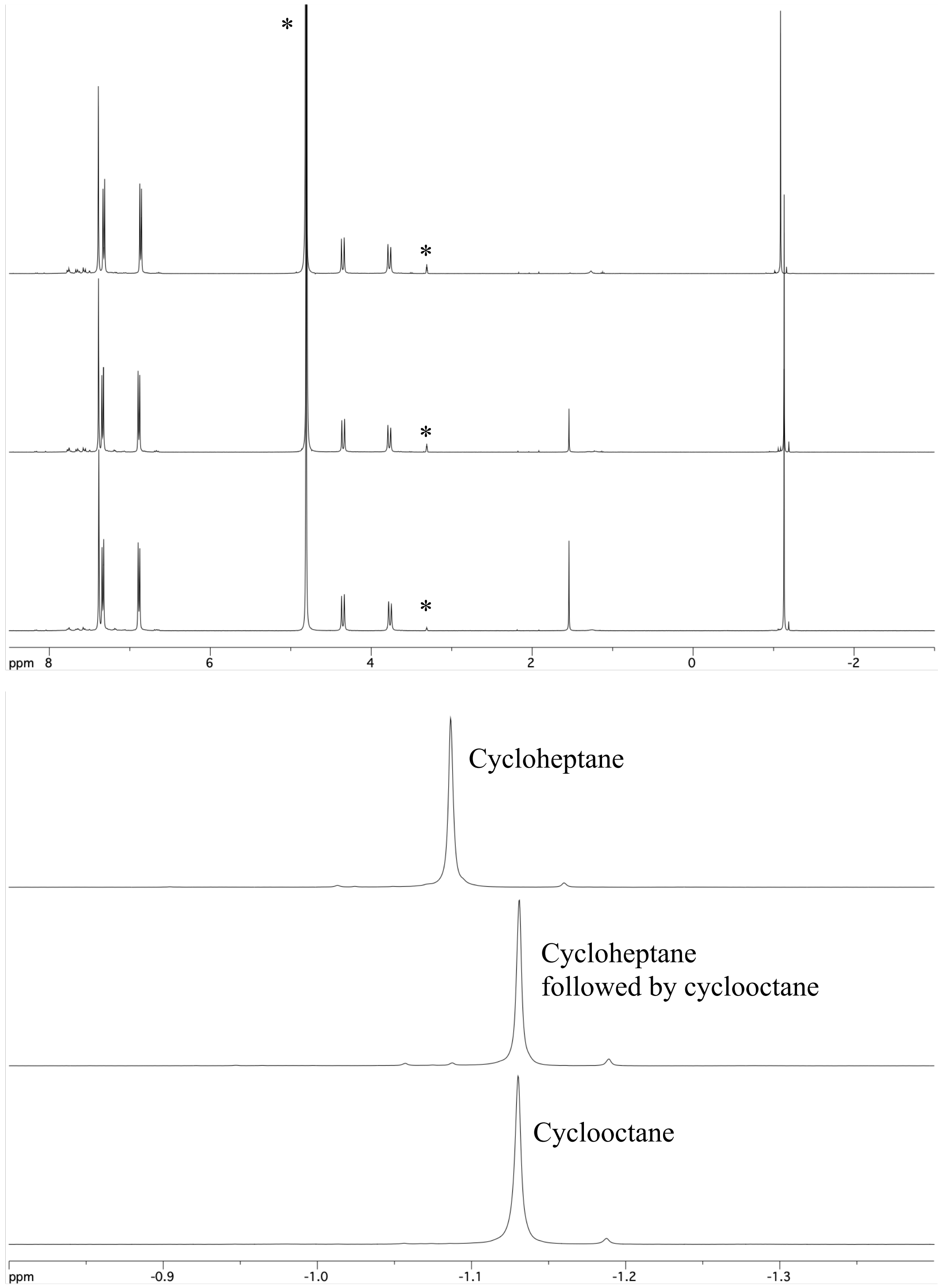

Figure S18: ^{1}H NMR spectra of **1** in 1 vol% CD_3OD in D_2O (4 mM) treated with 10 molar equivalents of cycloheptane (top), cyclooctane (bottom), and cycloheptane followed by the addition of 10 molar equivalents of cyclooctane (middle) (T = 298 K) (a). The spectra in (b) show the enlarged region of the spectra containing the signals of the bound guest.

^{1}H NMR Spectroscopic Investigation of *n*-Alkane Binding

(a)

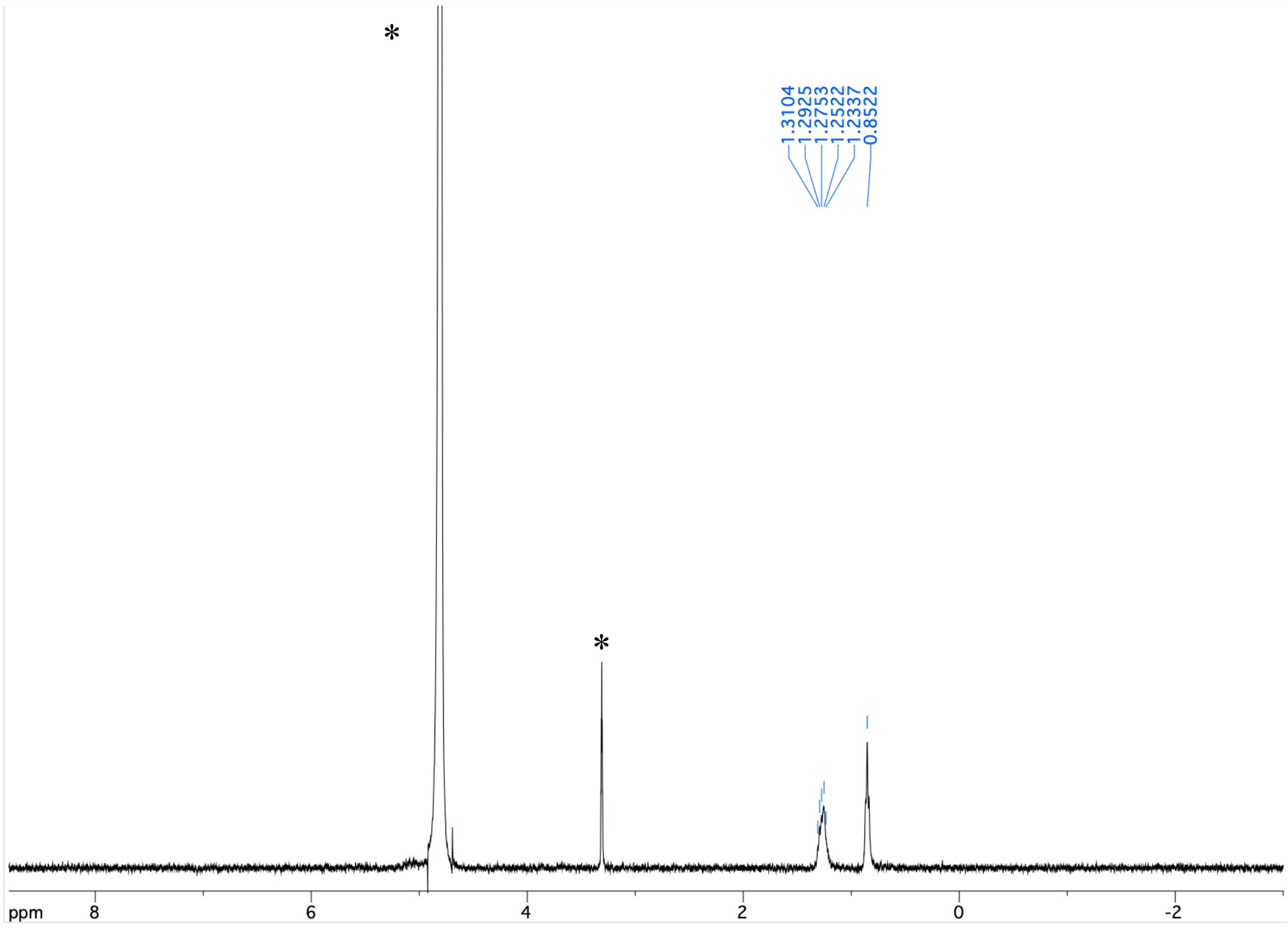

(b)

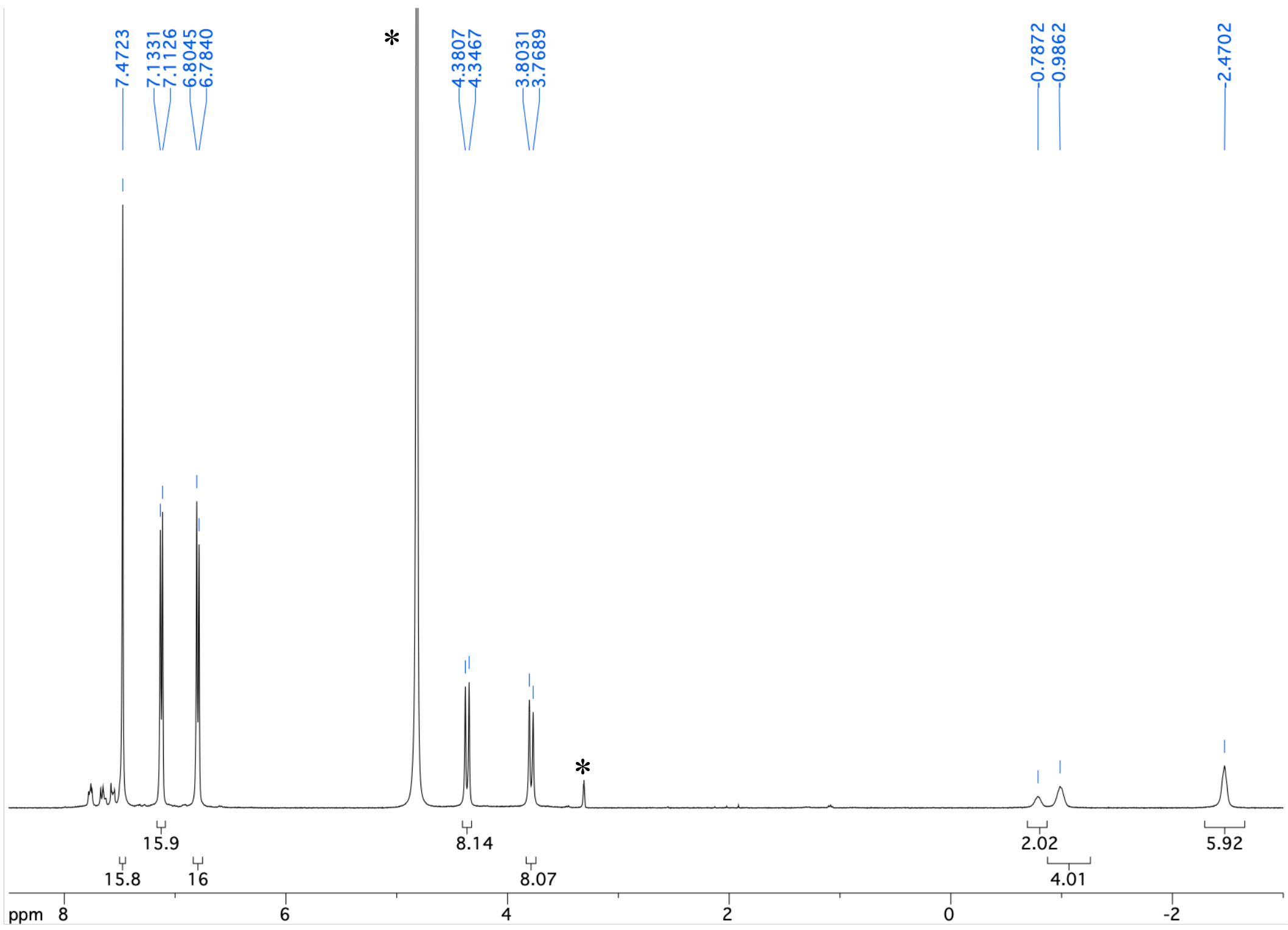

Figure S19: ^{1}H NMR spectra of *n*-pentane in 1 vol% CD_3OD in D_2O (a) and of **1** (4 mM) treated with 10 molar equivalents of *n*-pentane in the same solvent mixture (T = 298 K). The solvent signals are marked with asterisks.

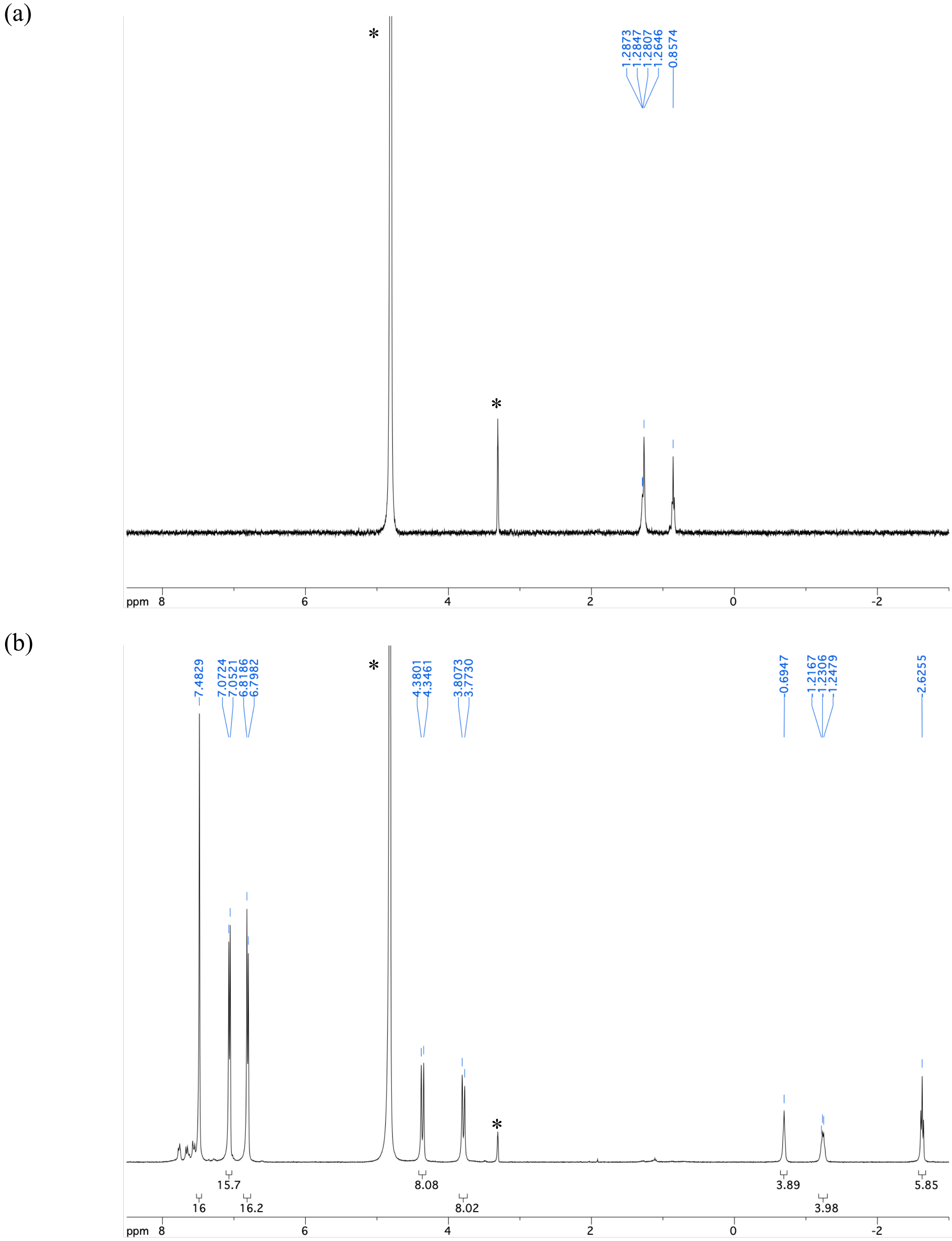

Figure S20: ^{1}H NMR spectra of *n*-hexane in 1 vol% CD_3OD in D_2O (a) and of **1** (4 mM) treated with 10 molar equivalents of *n*-hexane in the same solvent mixture (T = 298 K). The solvent signals are marked with asterisks.

(a)

(b)

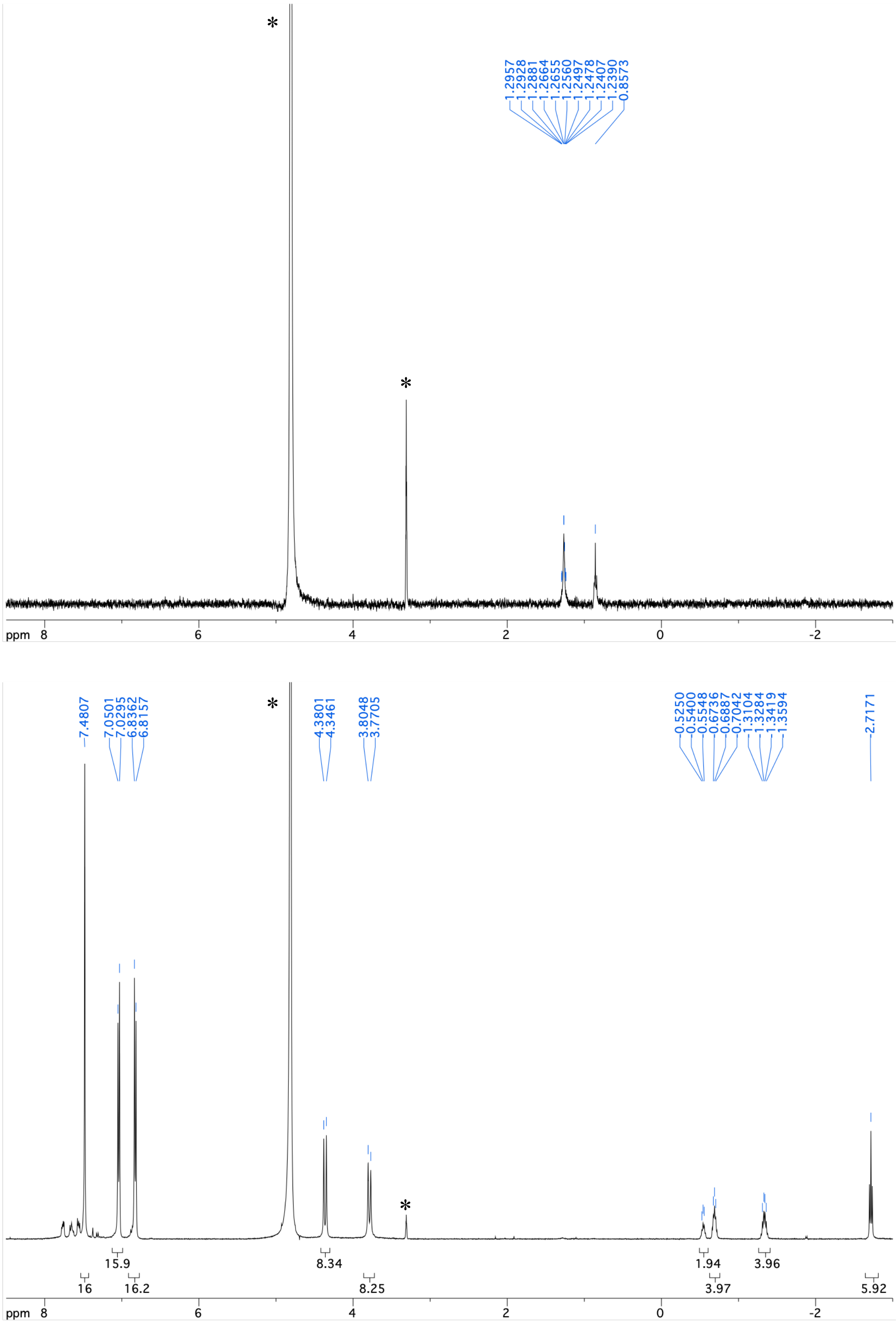

Figure S21: ^{1}H NMR spectra of *n*-heptane in 1 vol% CD_3OD in D_2O (a) and of **1** (4 mM) treated with 10 molar equivalents of *n*-heptane in the same solvent mixture (T = 298 K). The solvent signals are marked with asterisks.

(a)

(b)

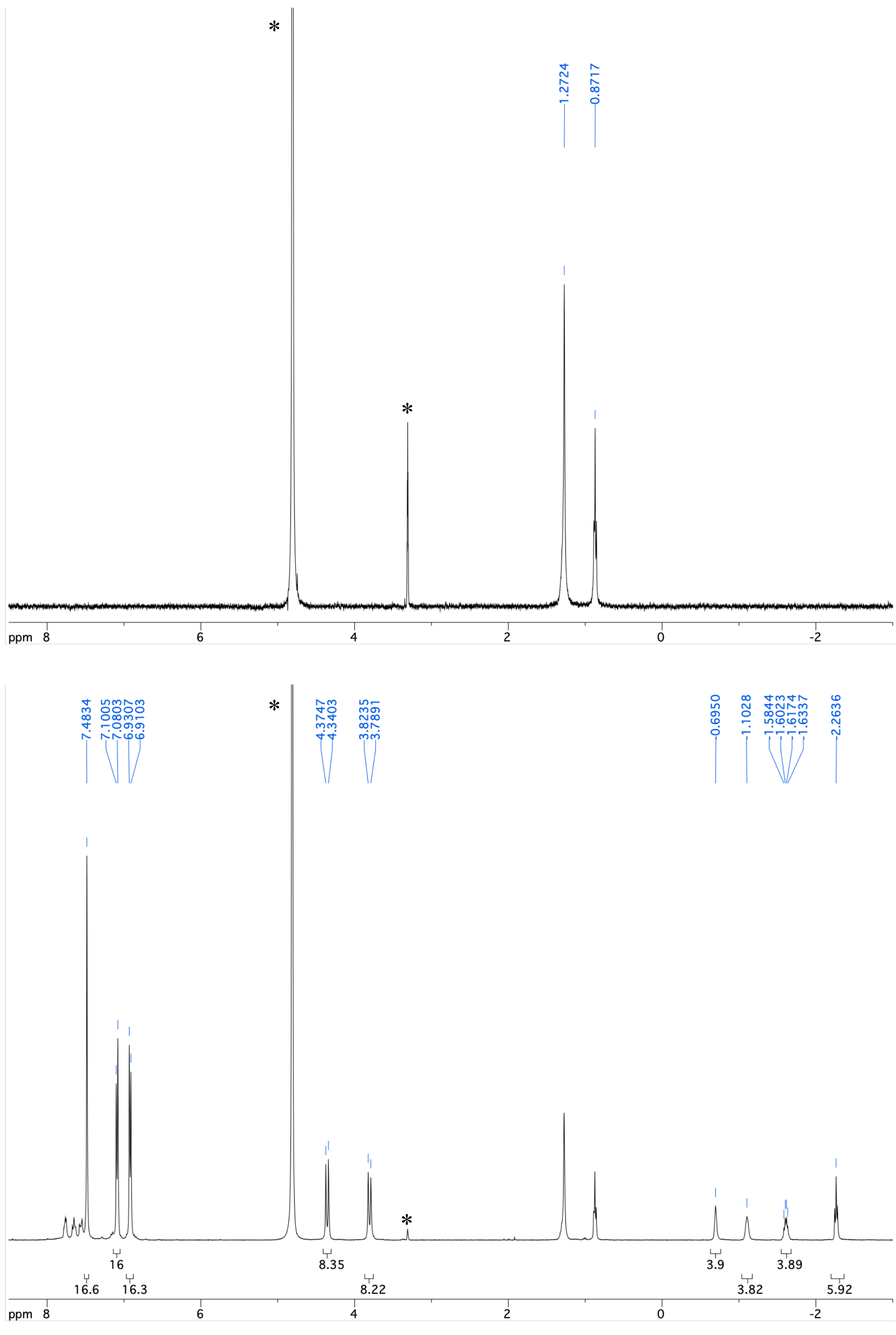

Figure S22: ^{1}H NMR spectra of *n*-octane in 1 vol% CD_3OD in D_2O (a) and of **1** (4 mM) treated with 10 molar equivalents of *n*-octane in the same solvent mixture (T = 298 K). The solvent signals are marked with asterisks.

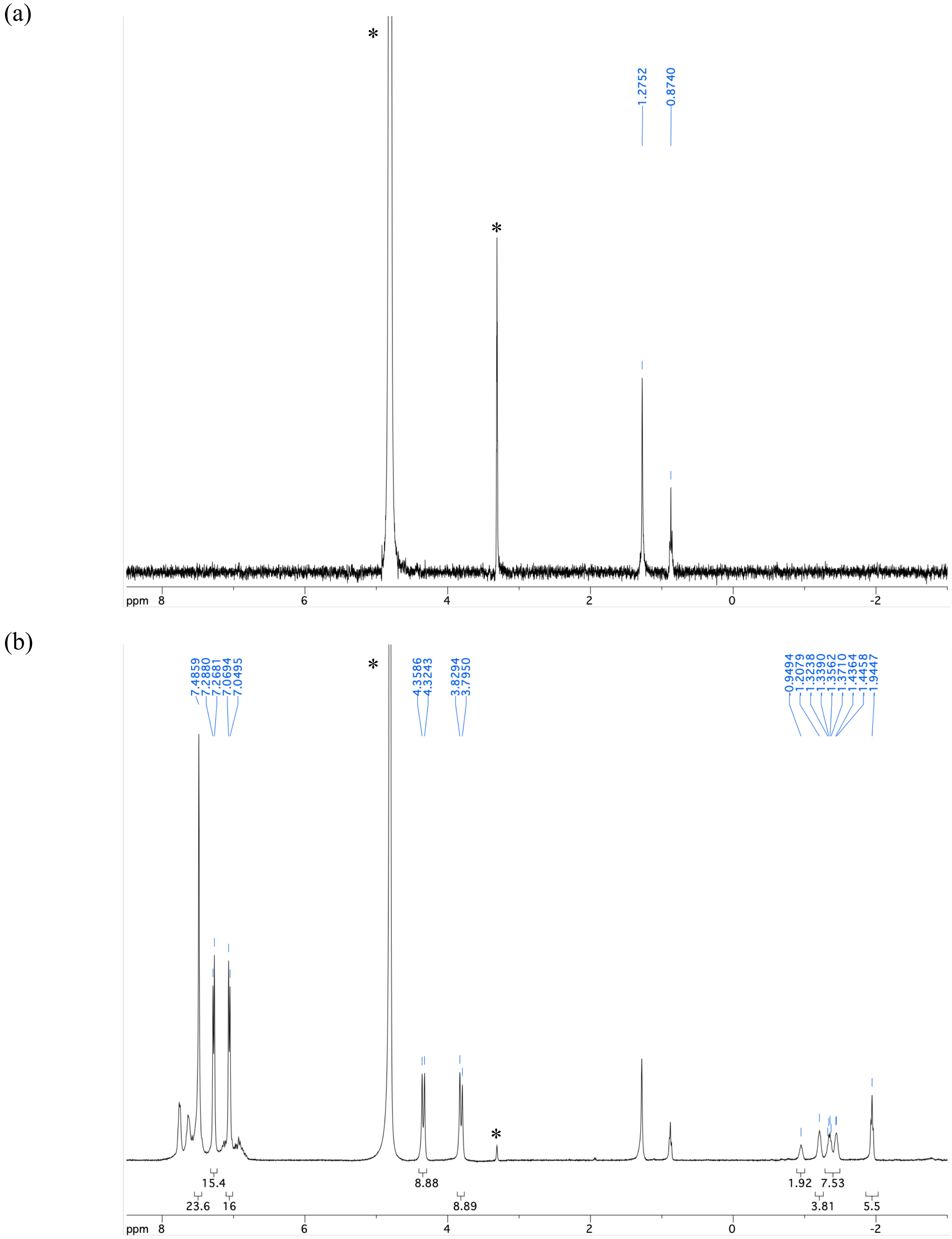

Figure S23: ^{1}H NMR spectra of *n*-nonane in 1 vol% CD_3OD in D_2O (a) and of **1** (4 mM) treated with 10 molar equivalents of *n*-nonane in the same solvent mixture (T = 298 K). The solvent signals are marked with asterisks.

(a)

(b)

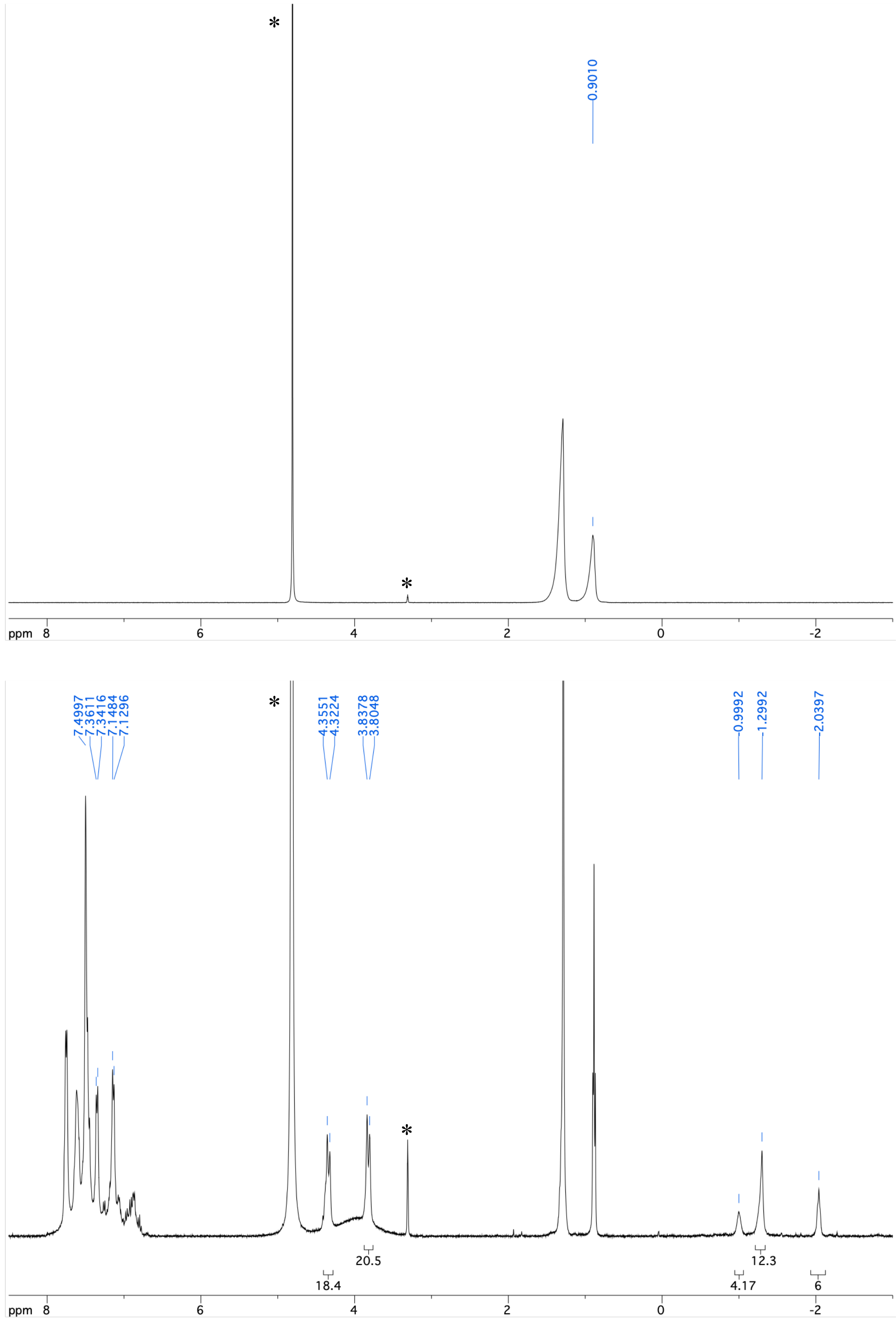

Figure S24: ¹H NMR spectra of *n*-decane in 1 vol% CD_3OD in D_2O (a) and of **1** (4 mM) treated with 10 molar equivalents of *n*-decane in the same solvent mixture (T = 298 K). The solvent signals are marked with asterisks.

Table S1: Chemical shifts in ppm of the calixarene and *n*-alkane protons in the spectra in Figures S19-S24. The chemical shifts of the free alkanes were measured using the same procedure but without the addition of **1**. The δ value refers to the shift of the terminal methyl protons. For the signal assignment, see Scheme 1 in the main manuscript. The chemical shifts of the apparent doublets of the aromatic protons and the doublets of the methylene protons are specified as the center of the two signals.

	$\delta(H^a)$	$\delta(H^b)$	$\delta(H^c)$	$\delta(H^d)$	$\delta(H^e)$	δ(free guest)	δ (bound guest)	$\Delta\delta$[a]
n-Pentane	7.47	6.79	7.12	4.36	3.79	0.85	-2.47	3.32
n- Hexane	7.48	6.81	7.06	4.36	3.79	0.86	-2.63	3.48
n-Heptane	7.48	6.83	7.04	4.36	3.79	0.86	-2.72	3.57
n-Octane	7.48	6.92	7.09	4.36	3.81	0.87	-2.26	3.13
n-Nonane	7.49	7.06	7.27	4.34	3.81	0.87	-1.94	2.82
n-Decane	7.50	7.14	7.35	4.34	3.82	0.90	-2.04	2.94

[a] $\Delta\delta = \delta$(free guest)–δ(bound guest)

ROESY Spectra of *n*-Alkane Complexes

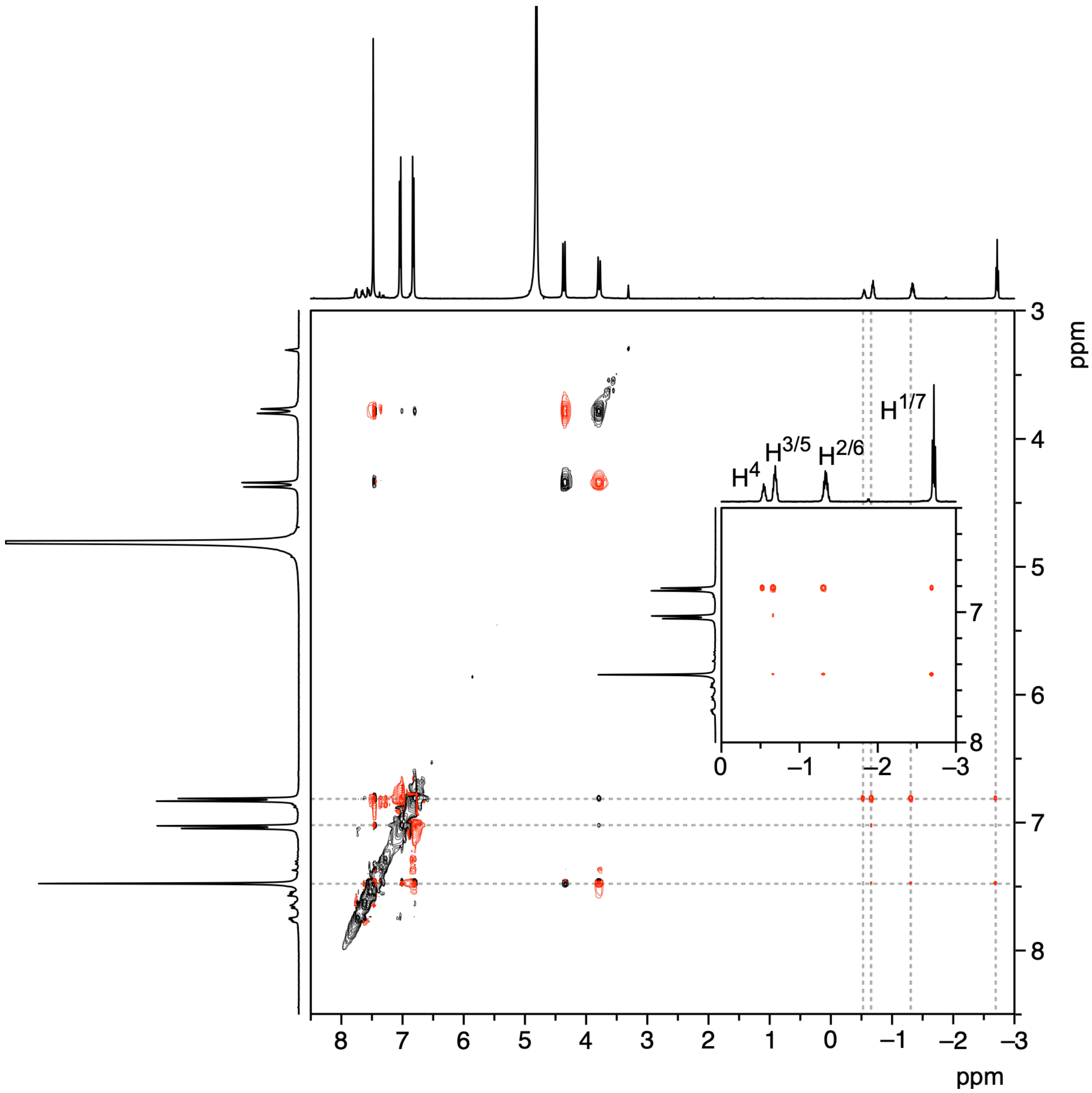

Figure S25: ROESY spectrum of **1** (4 mM) treated with 10 molar equivalents of *n*-heptane in 1 vol% CD_3OD in D_2O (T = 298 K). Mixing time 500 ms. The inset shows the cross peaks between the signal of the bound guest and the aromatic signals of **1**.

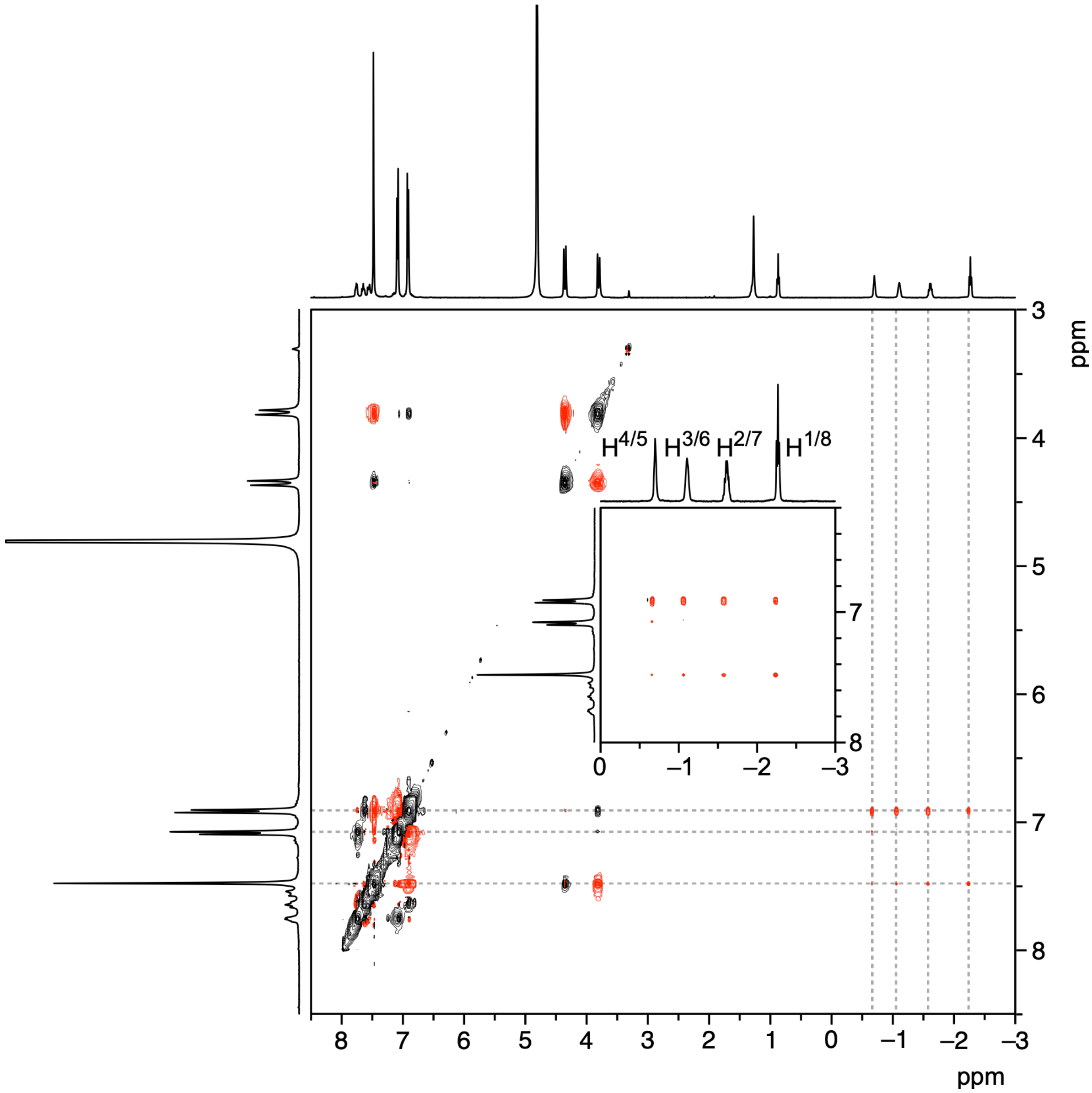

Figure S26: ROESY spectrum of **1** (4 mM) treated with 10 molar equivalents of *n*-octane in 1 vol% CD_3OD in D_2O (T = 298 K). Mixing time 500 ms. The inset shows the cross peaks between the signal of the bound guest and the aromatic signals of **1**.

DOSY Spectrum of *n*-Heptane Complex

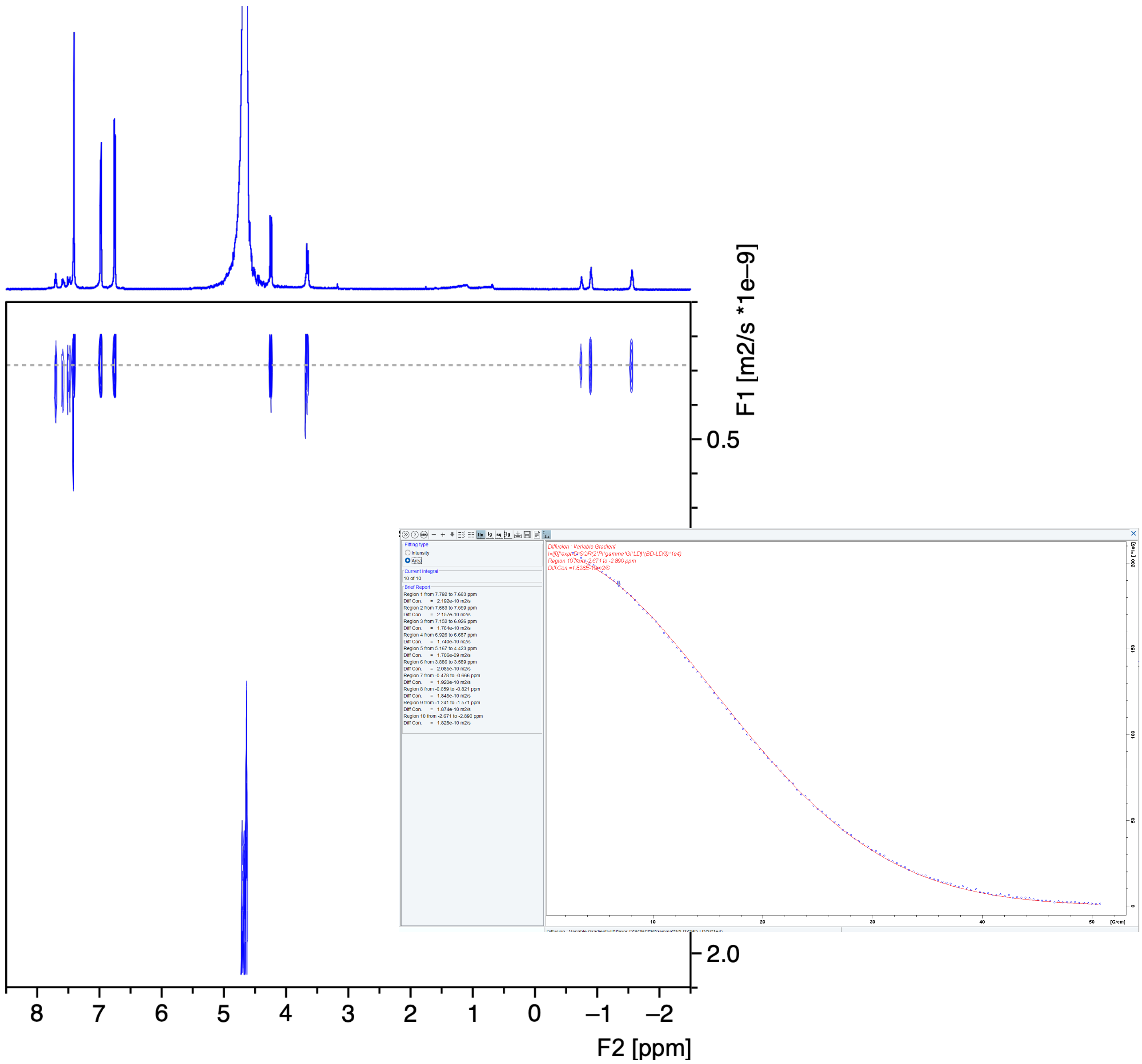

Figure S27: DOSY spectrum of **1** (4 mM) treated with 10 molar equivalents of *n*-heptane in 1 vol% CD_3OD in D_2O (T = 300 K). The graph shows the non-normalized fitted D values for a selection of peaks.

Table S2: Averaged diffusion coefficients D_{avg} in $m^2 s^{-1}$ derived from the DOSY spectra of the cycloalkane complexes (Fig. S12–S15) and the *n*-heptane complex (Fig. S27) for the signals assigned to the capsule of **1** and the bound guest.

	D_{avg}(**1**) × 10^{10}	D_{avg}(bound guest) × 10^{10}
Cyclopentane	1.72	2.61
Cyclohexane	1.79	2.23
Cycloheptane	1.73	1.69
Cyclooctane	1.75	1.75
n-Heptane	1.74	1.87

^{1}H NMR Spectra of Competition Experiments with *n*-Alkanes

(a)

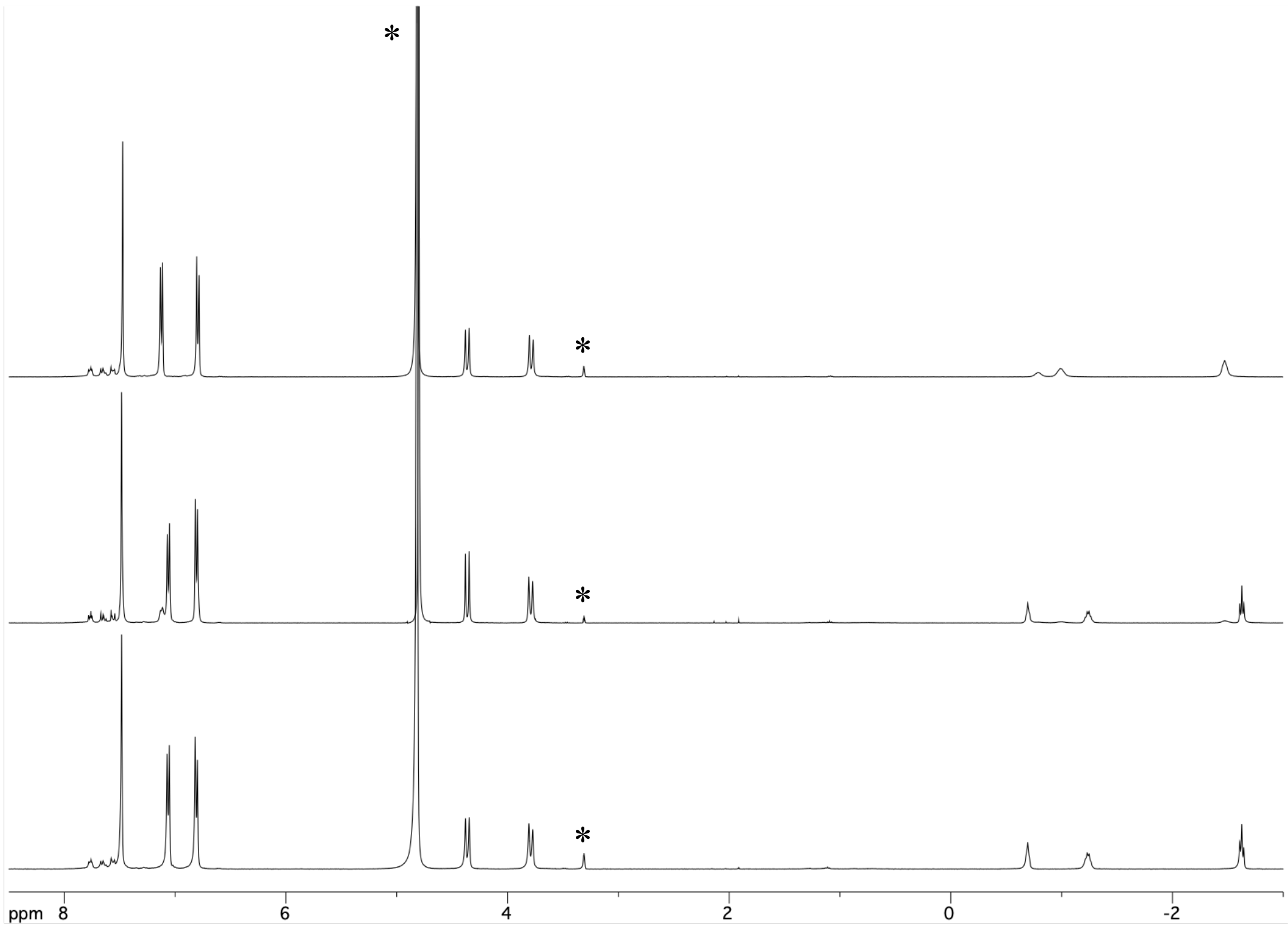

(b)

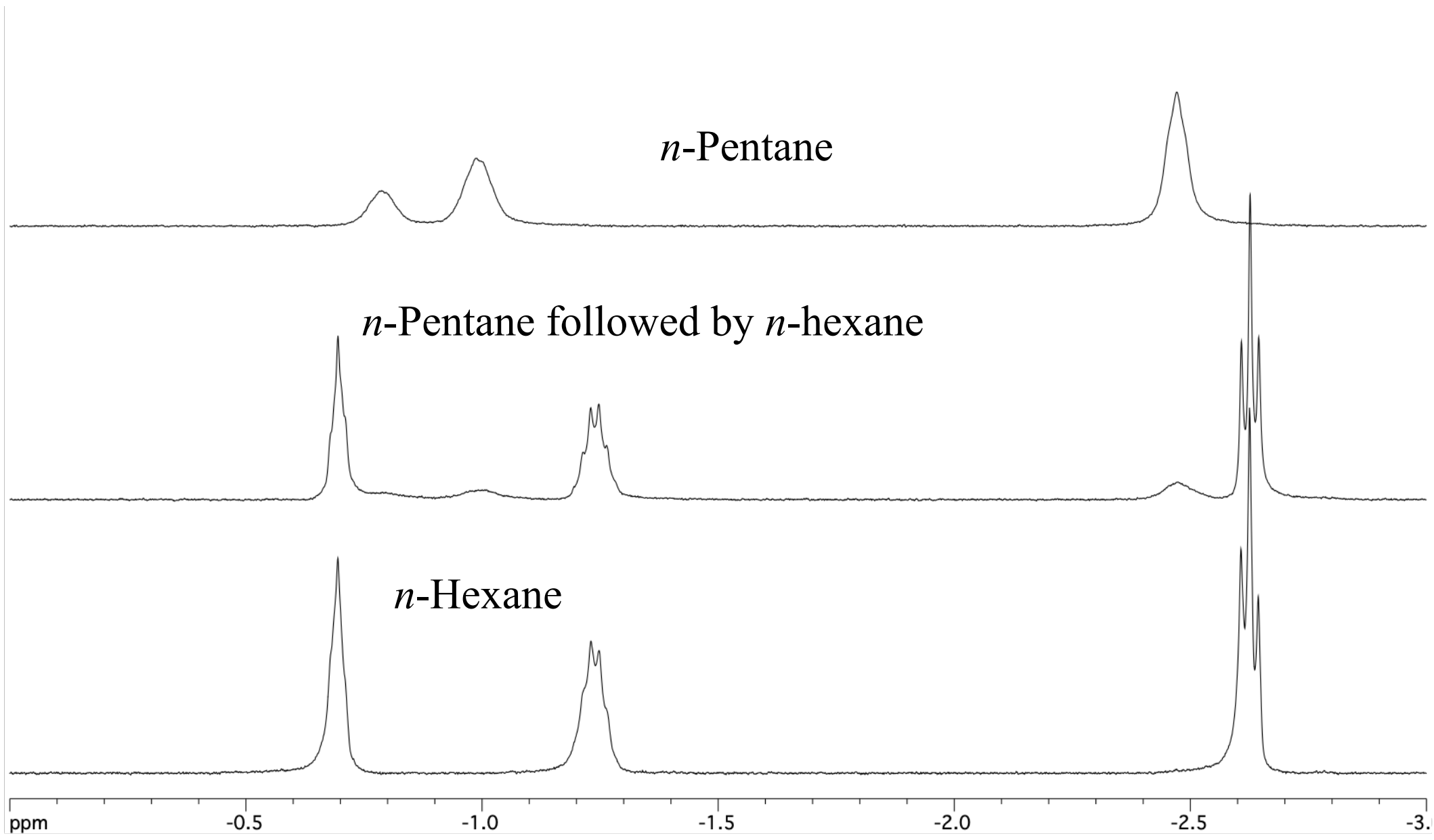

Figure S28: ^{1}H NMR spectra of **1** in 1 vol% CD_3OD in D_2O (4 mM) treated with 10 molar equivalents of *n*-pentane (top), *n*-hexane (bottom), and *n*-pentane followed by the addition of 10 molar equivalents of *n*-hexane (middle) (T = 298 K) (a). The spectra in (b) show the enlarged region of the spectra containing the signals of the bound guest.

(a)

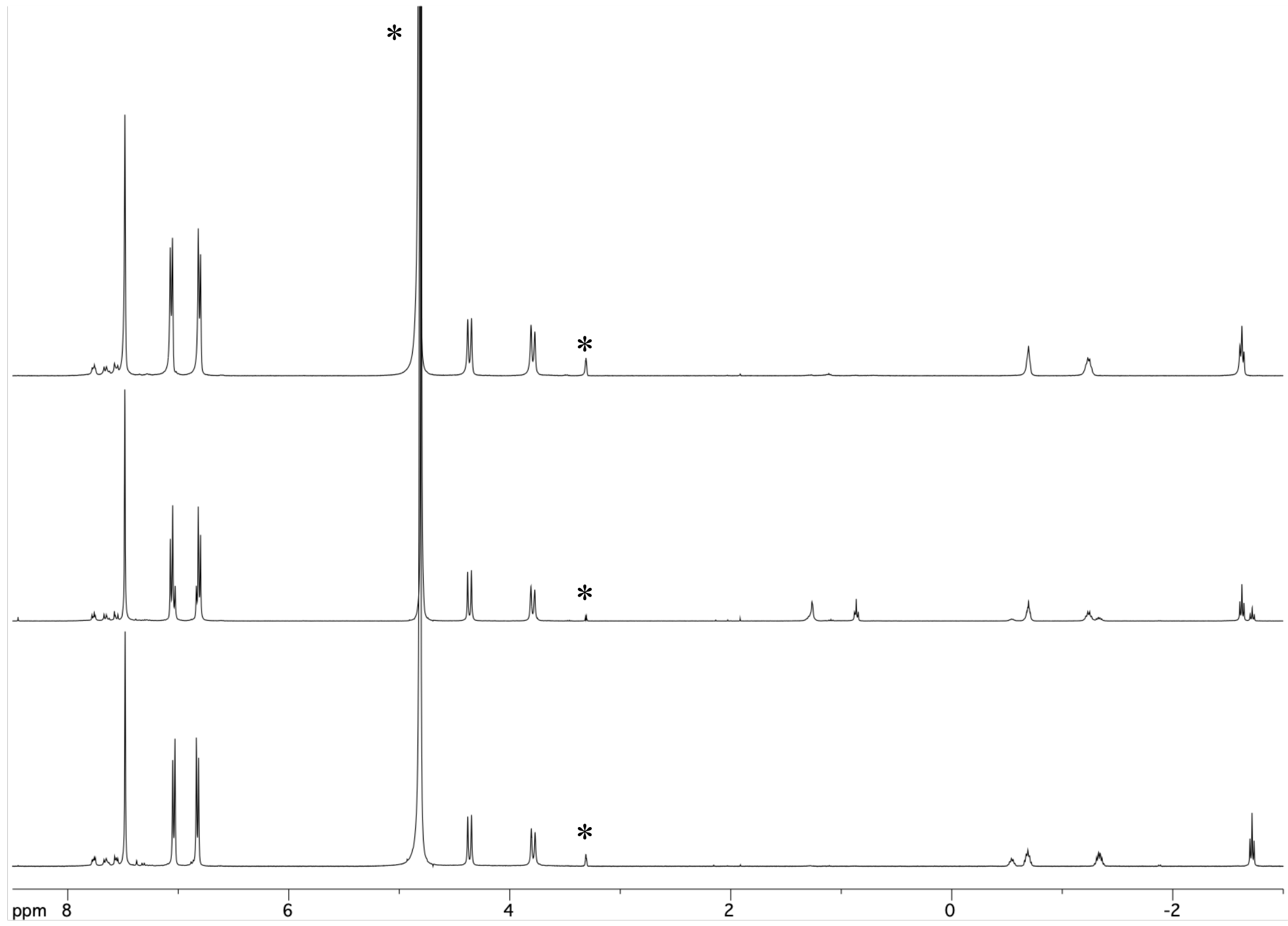

(b)

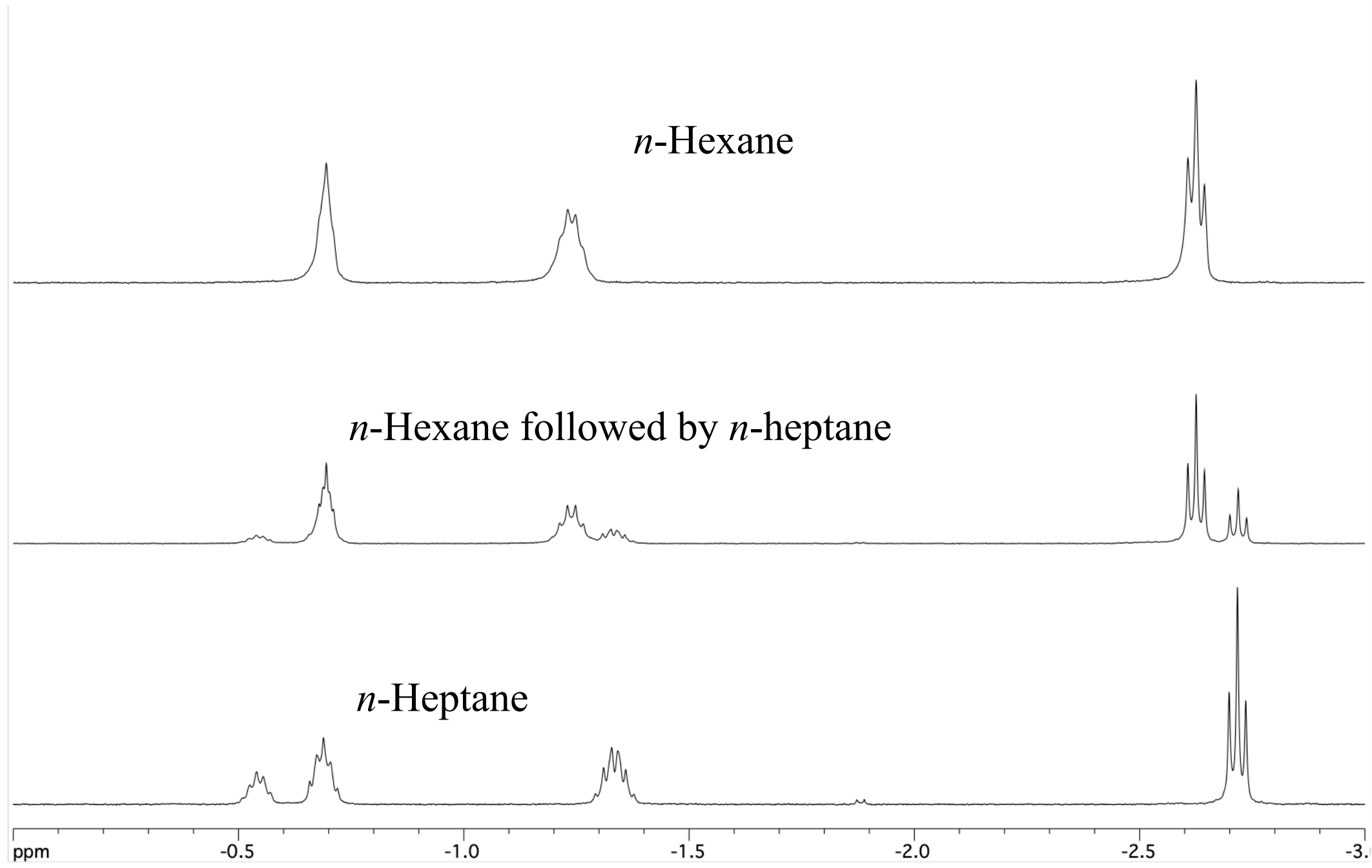

Figure S29: ^{1}H NMR spectra of **1** in 1 vol% CD_3OD in D_2O (4 mM) treated with 10 molar equivalents of *n*-hexane (top), *n*-heptane (bottom), and *n*-hexane followed by the addition of 10 molar equivalents of *n*-heptane (middle) (T = 298 K) (a). The spectra in (b) show the enlarged region of the spectra containing the signals of the bound guest.

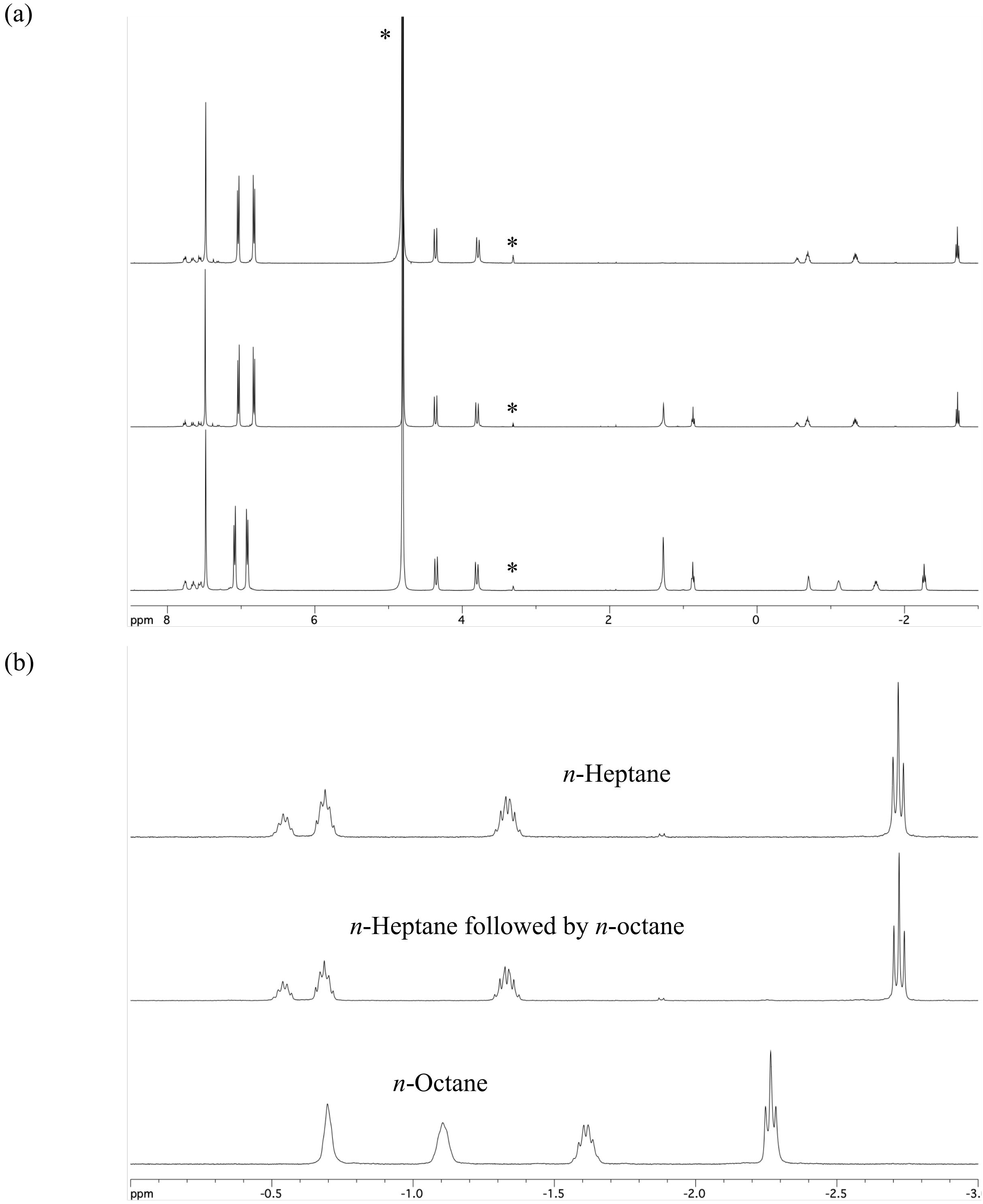

Figure S30: ^{1}H NMR spectra of **1** in 1 vol% CD_3OD in D_2O (4 mM) treated with 10 molar equivalents of *n*-heptane (top), *n*-octane (bottom), and *n*-heptane followed by the addition of 10 molar equivalents of *n*-octane (middle) (T = 298 K) (a). The spectra in (b) show the enlarged region of the spectra containing the signals of the bound guest.

(a)

(b)

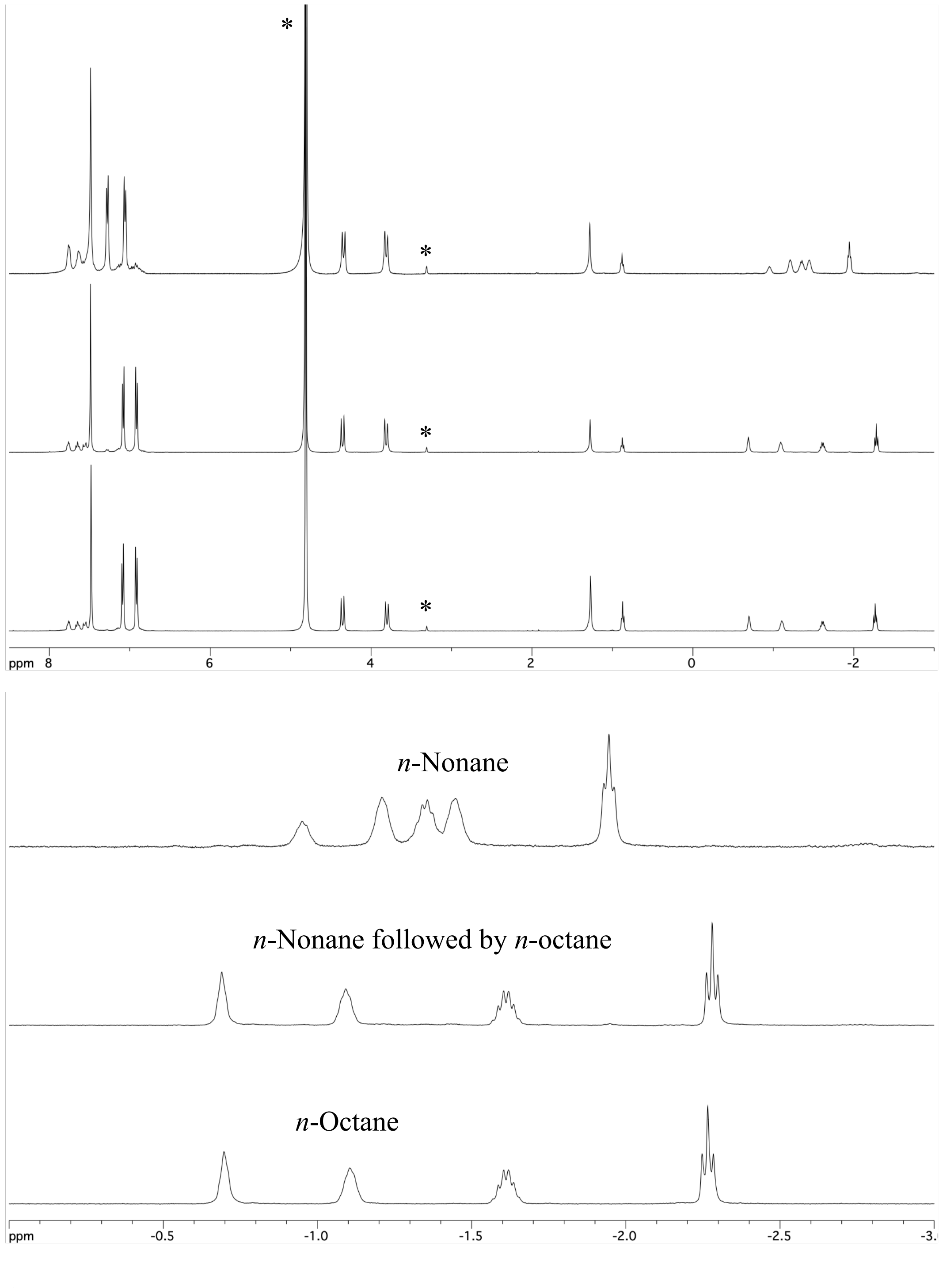

Figure S31: ^{1}H NMR spectra of **1** in 1 vol% CD_3OD in D_2O (4 mM) treated with 10 molar equivalents of *n*-nonane (top), *n*-octane (bottom), and *n*-nonane followed by the addition of 10 molar equivalents of *n*-octane (middle) (T = 298 K) (a). The spectra in (b) show the enlarged region of the spectra containing the signals of the bound guest.

(a)

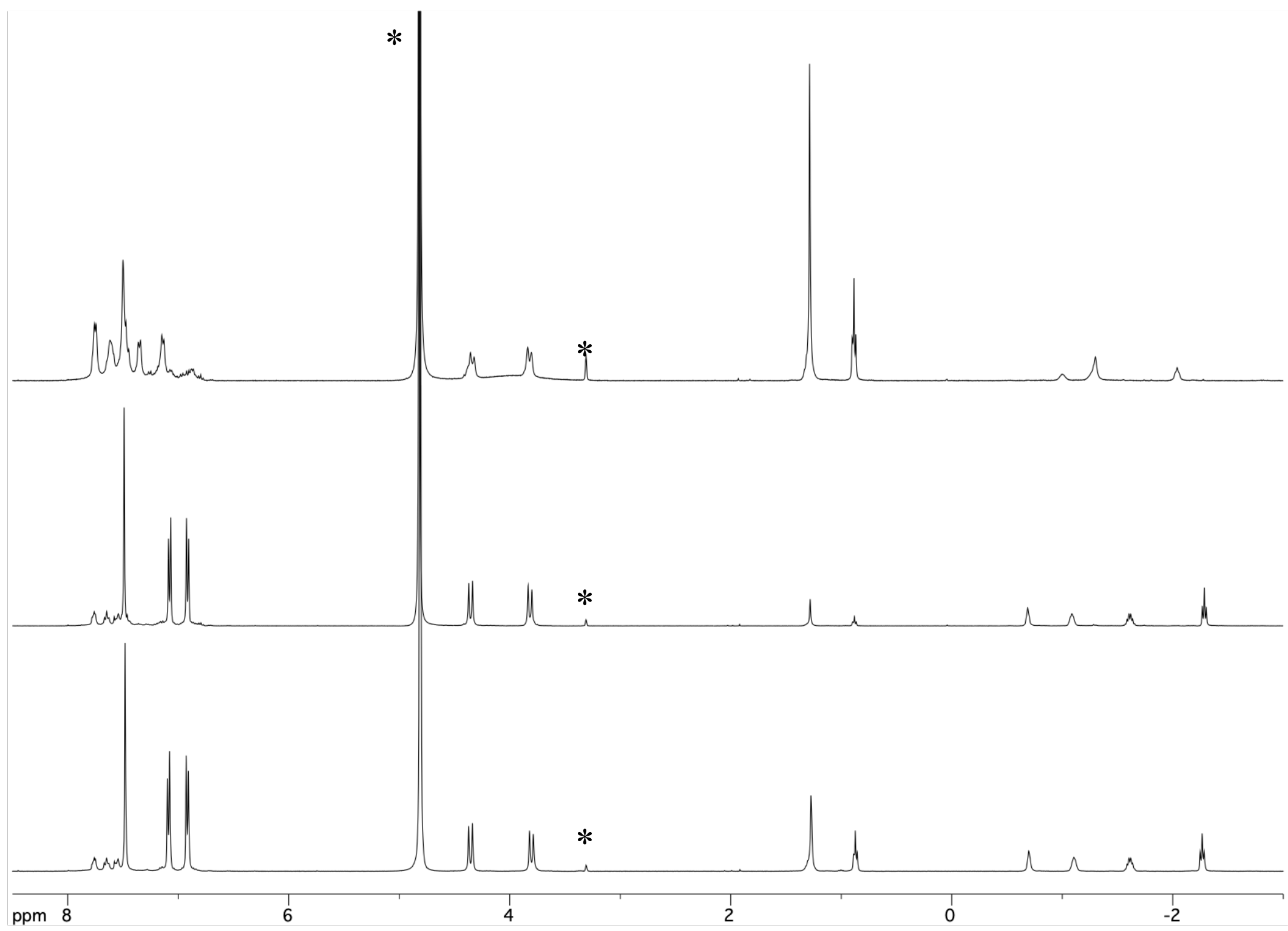

(b)

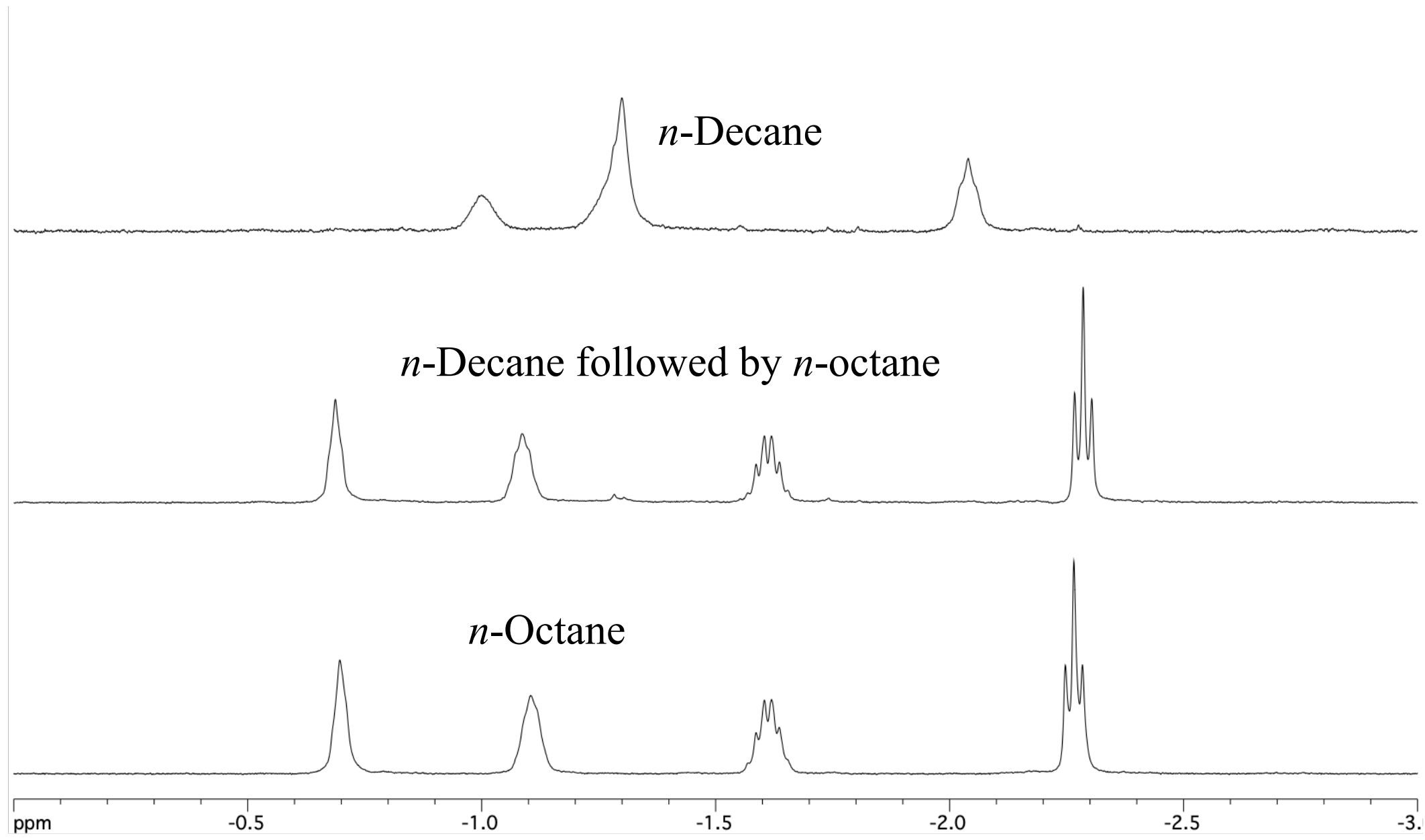

Figure S32: ^{1}H NMR spectra of **1** in 1 vol% CD_3OD in D_2O (4 mM) treated with 10 molar equivalents of *n*-decane (top), *n*-octane (bottom), and *n*-decane followed by the addition of 10 molar equivalents of *n*-octane (middle) (T = 298 K) (a). The spectra in (b) show the enlarged region of the spectra containing the signals of the bound guest.

^{1}H NMR Spectra of Competition Experiments with *n*-Alkanes/Cycloalkanes

(a)

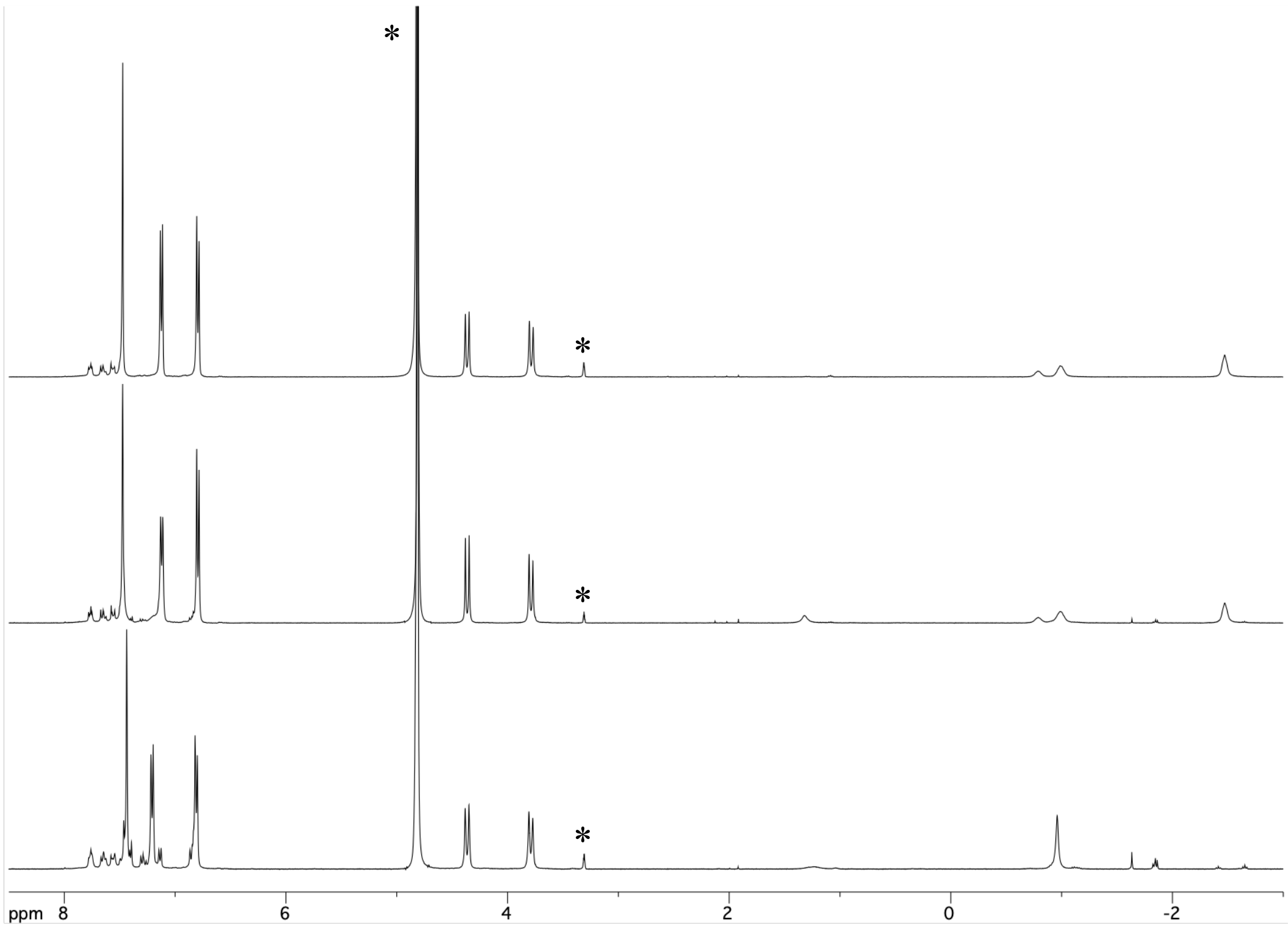

(b)

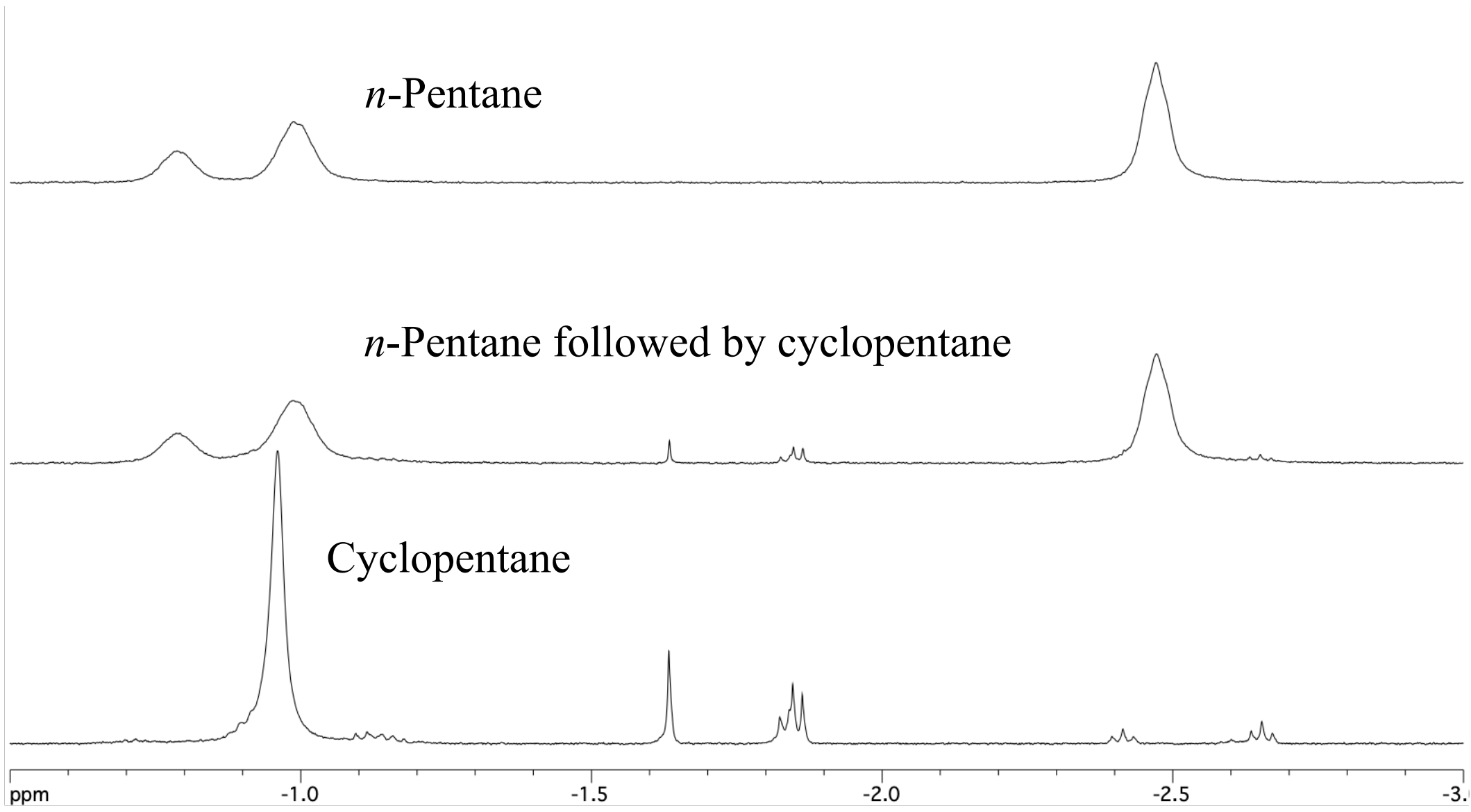

Figure S33: ^{1}H NMR spectra of **1** in 1 vol% CD_3OD in D_2O (4 mM) treated with 10 molar equivalents of *n*-pentane (top), cyclopentane (bottom), and *n*-pentane followed by the addition of 10 molar equivalents of cyclopentane (middle) (T = 298 K) (a). The spectra in (b) show the enlarged region of the spectra containing the signals of the bound guest.

(a)

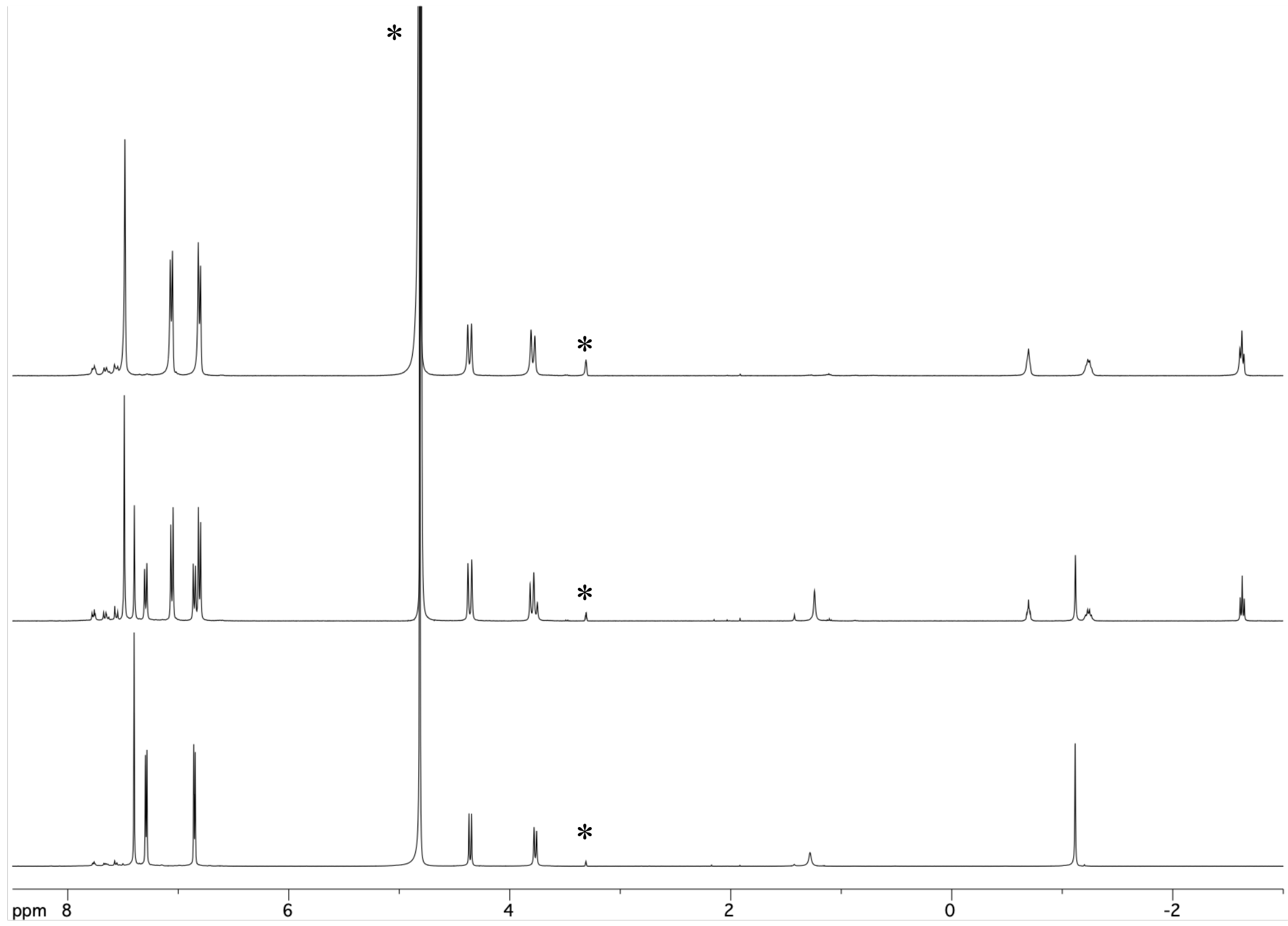

(b)

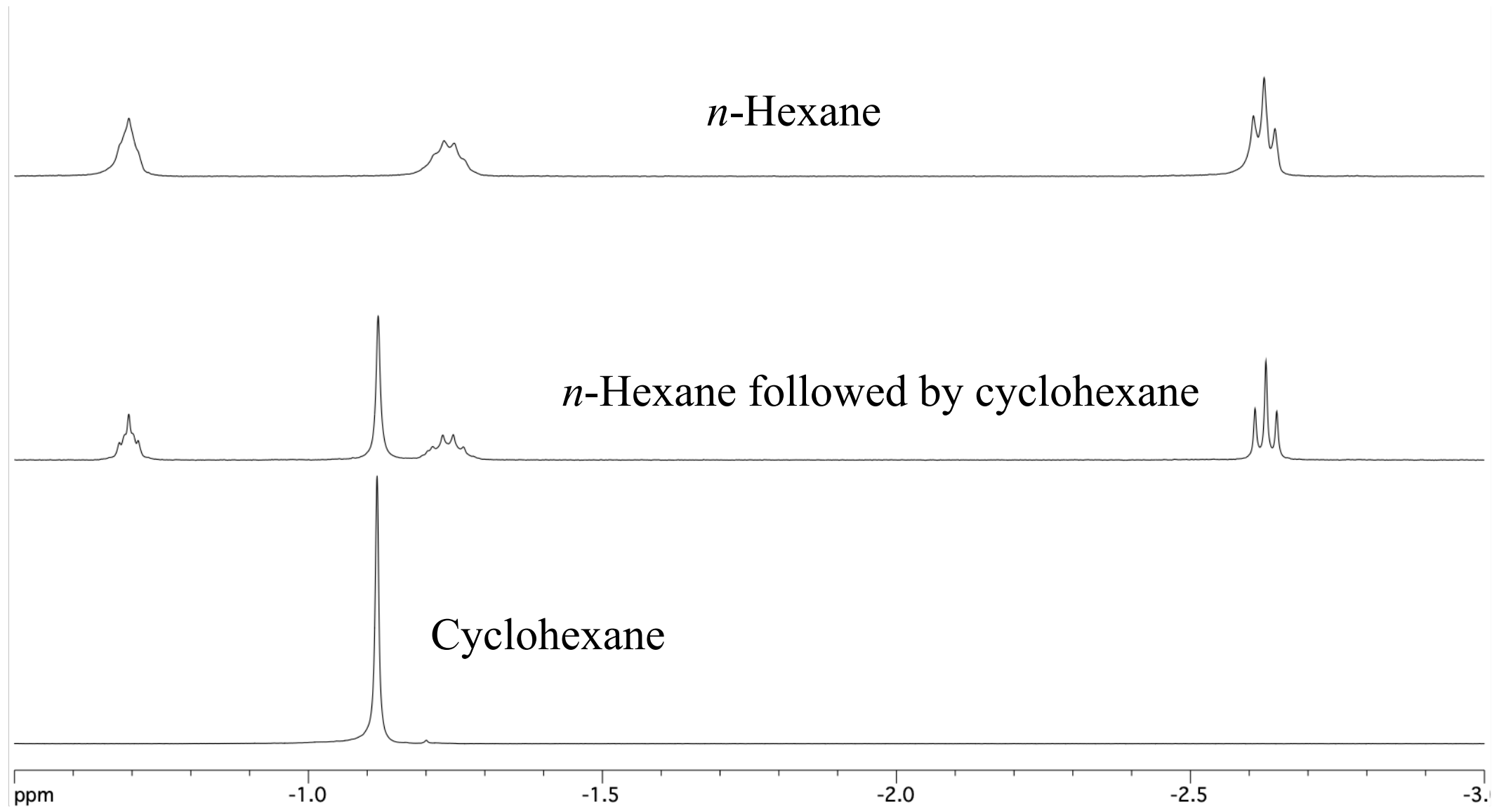

Figure S34: ^{1}H NMR spectra of **1** in 1 vol% CD_3OD in D_2O (4 mM) treated with 10 molar equivalents of *n*-hexane (top), cyclohexane (bottom), and *n*-hexane followed by the addition of 10 molar equivalents of cyclohexane (middle) (T = 298 K) (a). The spectra in (b) show the enlarged region of the spectra containing the signals of the bound guest.

(a)

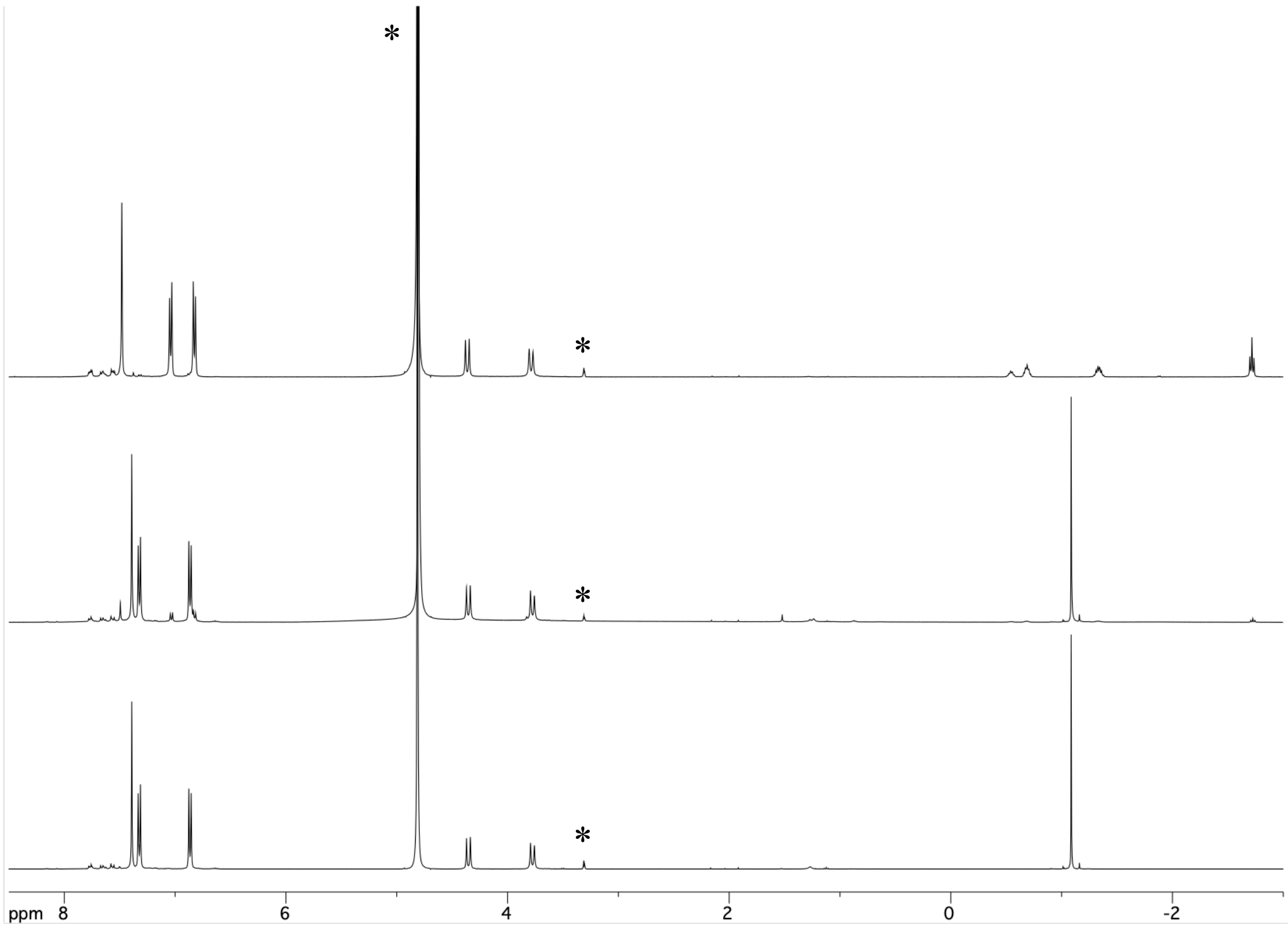

(b)

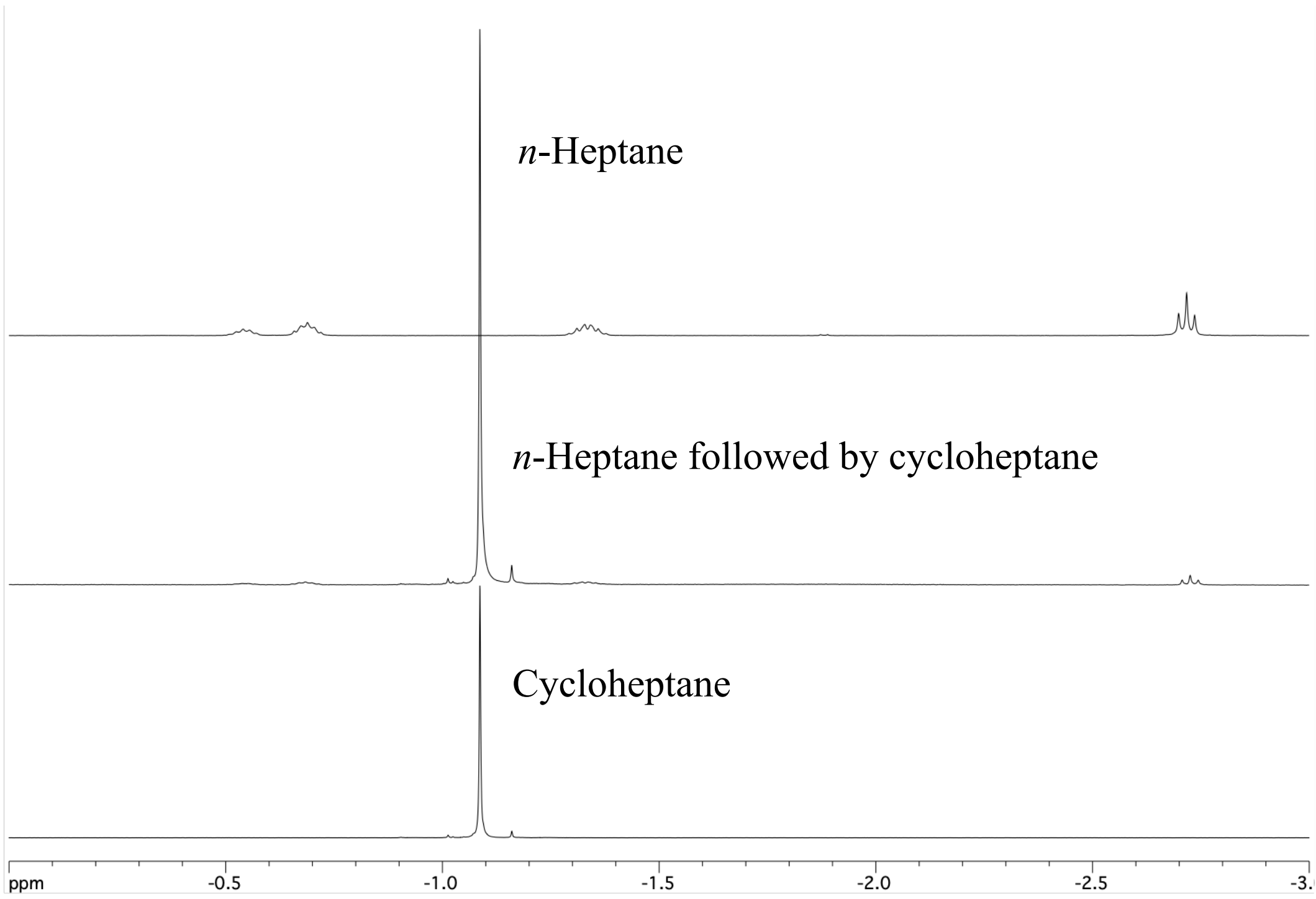

Figure S35: ^{1}H NMR spectra of **1** in 1 vol% CD_3OD in D_2O (4 mM) treated with 10 molar equivalents of *n*-heptane (top), cycloheptane (bottom), and *n*-heptane followed by the addition of 10 molar equivalents of cycloheptane (middle) (T = 298 K) (a). The spectra in (b) show the enlarged region of the spectra containing the signals of the bound guest.

(a)

(b)

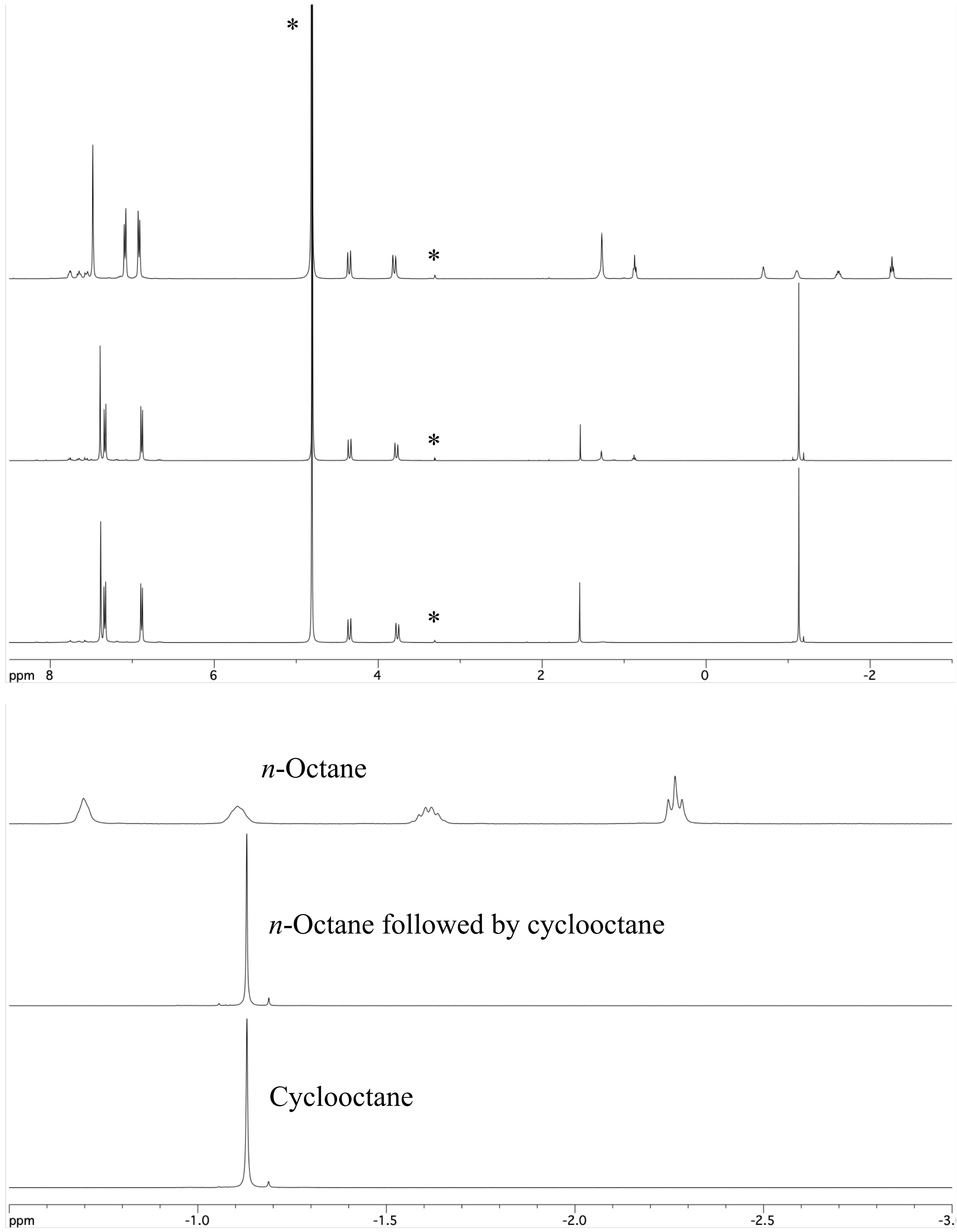

Figure S36: ^{1}H NMR spectra of **1** in 1 vol% CD_3OD in D_2O (4 mM) treated with 10 molar equivalents of *n*-octane (top), cyclooctane (bottom), and *n*-octane followed by the addition of 10 molar equivalents of cyclooctane (middle) (T = 298 K) (a). The spectra in (b) show the enlarged region of the spectra containing the signals of the bound guest.

^{1}H NMR Spectroscopic Investigation of Hexafluorobenzene Binding

(a)

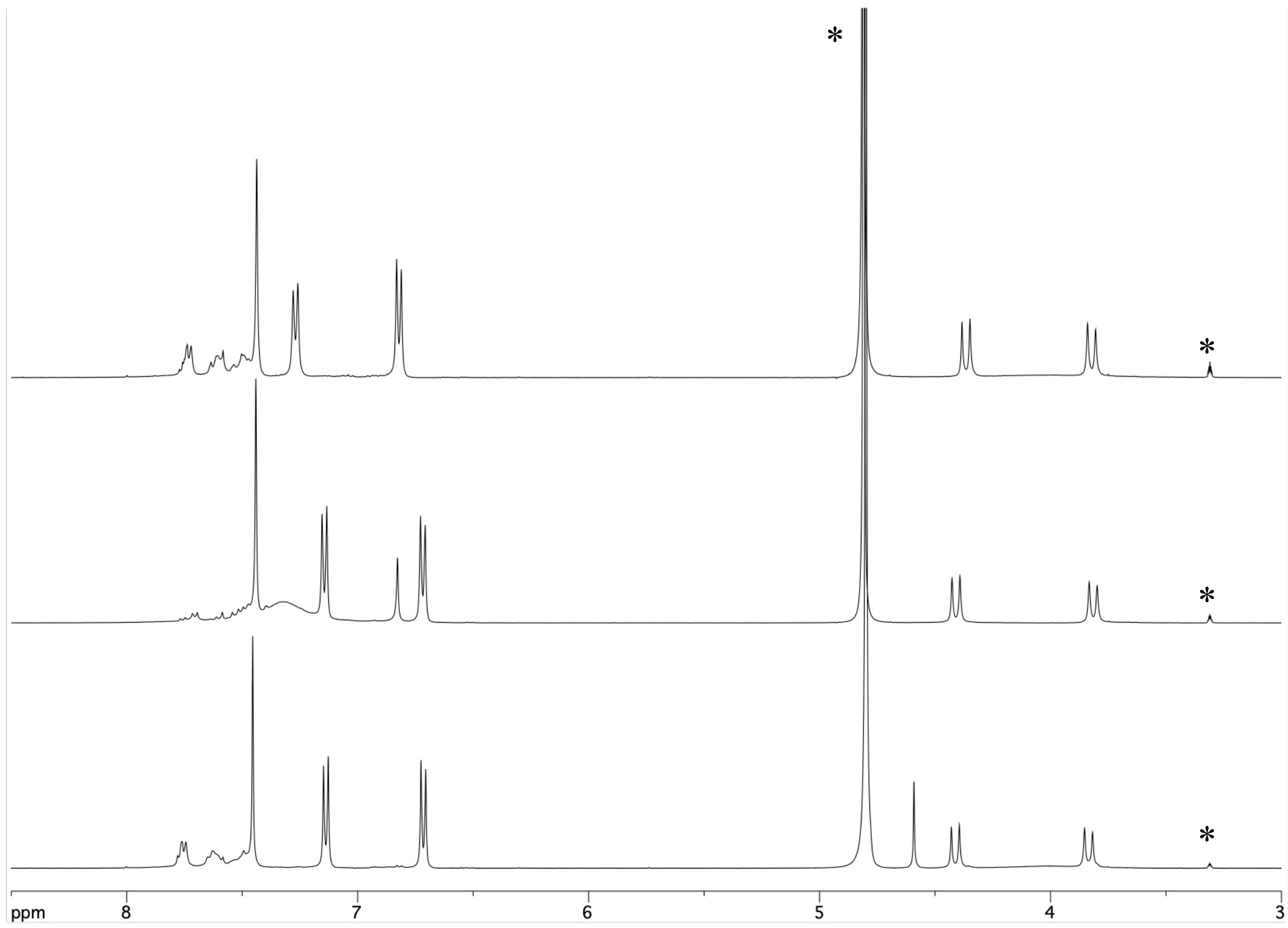

(b)

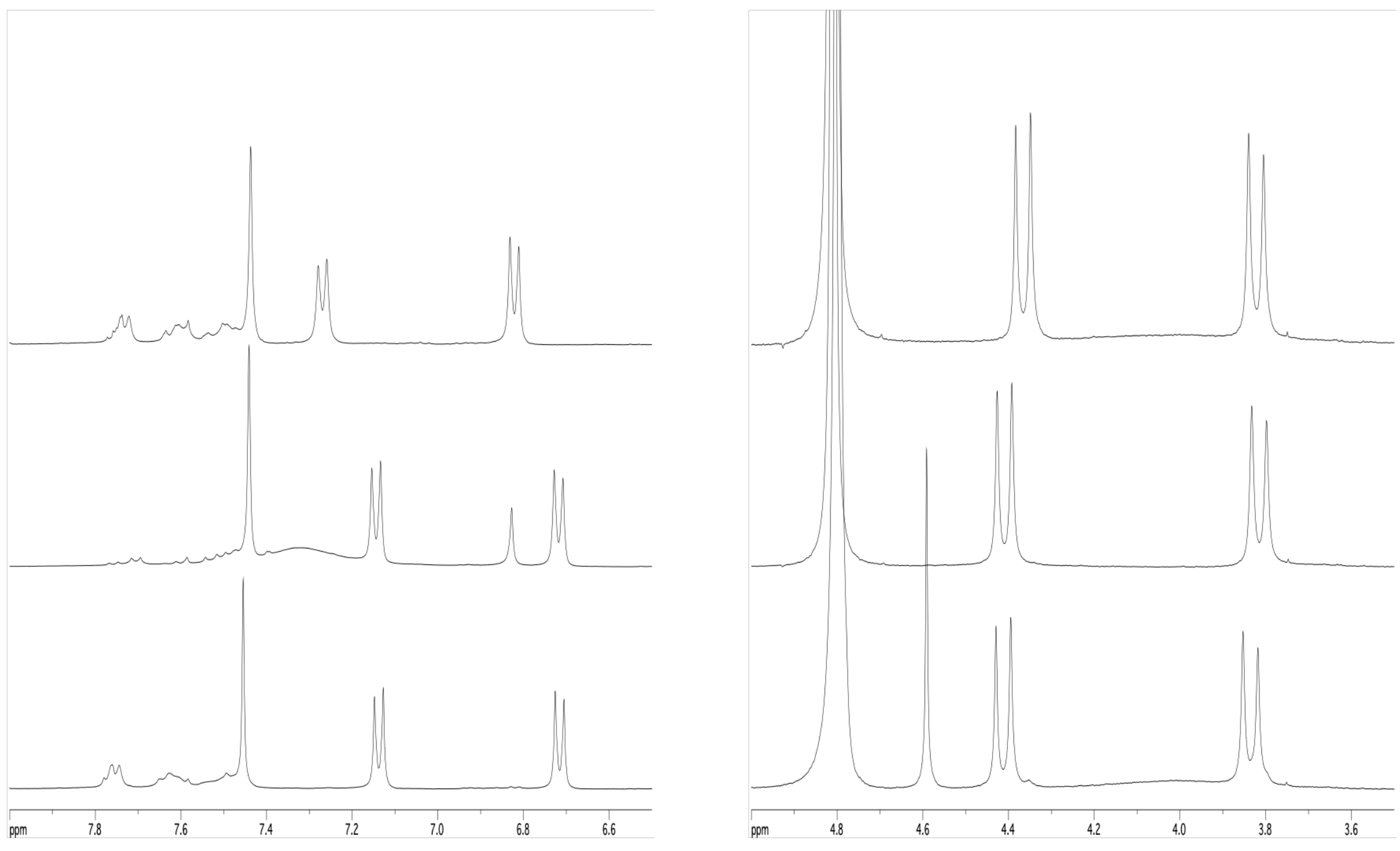

Figure S37: ^{1}H NMR spectra of **1** in 1 vol% CD_3OD in D_2O (4 mM) in the presence of 10 molar equivalents of hexafluorobenzene (top), 10 molar equivalents of benzene (bottom), and 10 molar equivalents of hexafluorobenzene followed by 10 molar equivalents of benzene (middle) (T = 298 K) (a). The spectra in (b) show enlarged regions of the spectra in (a).

(a)

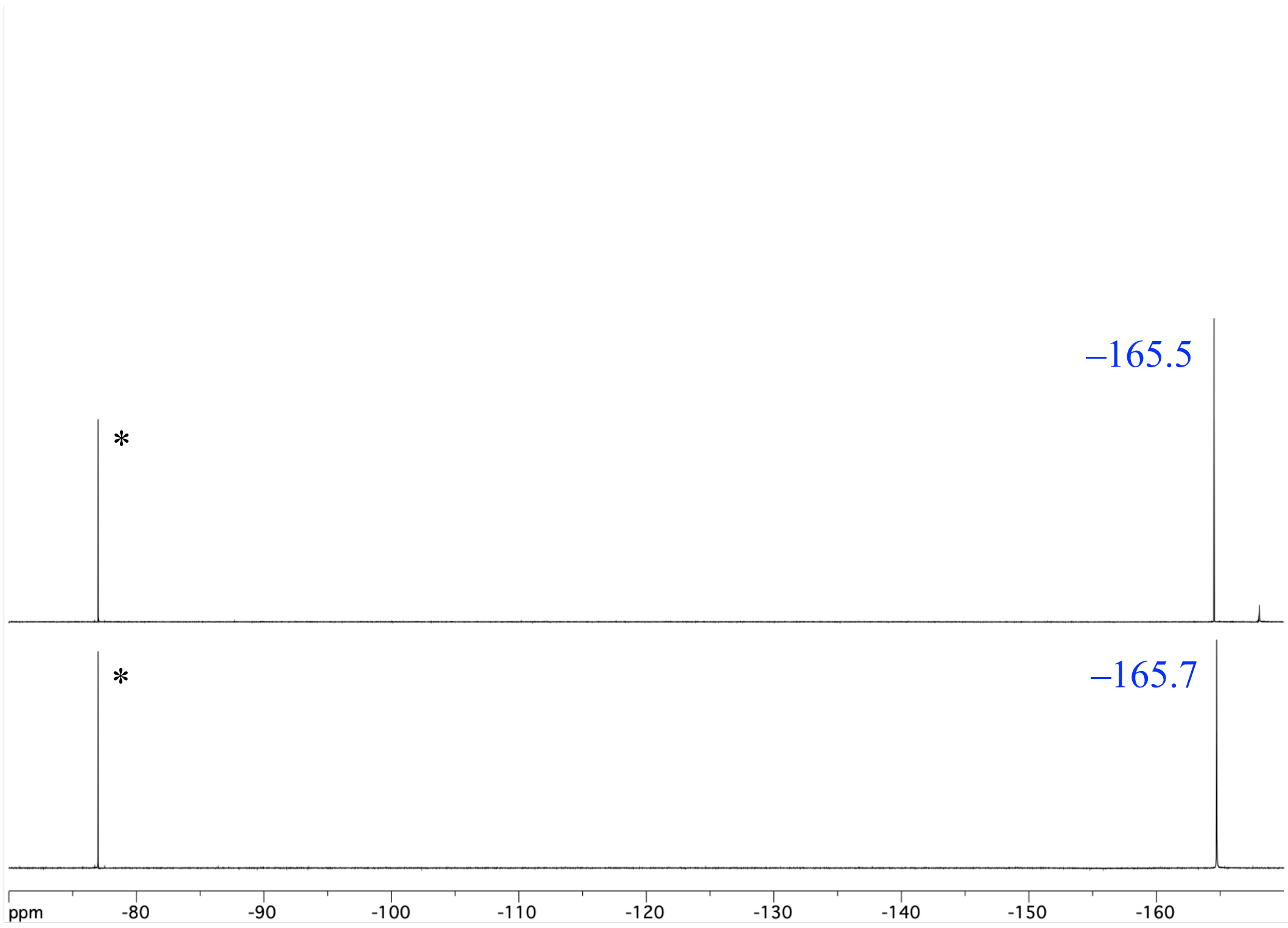

(b)

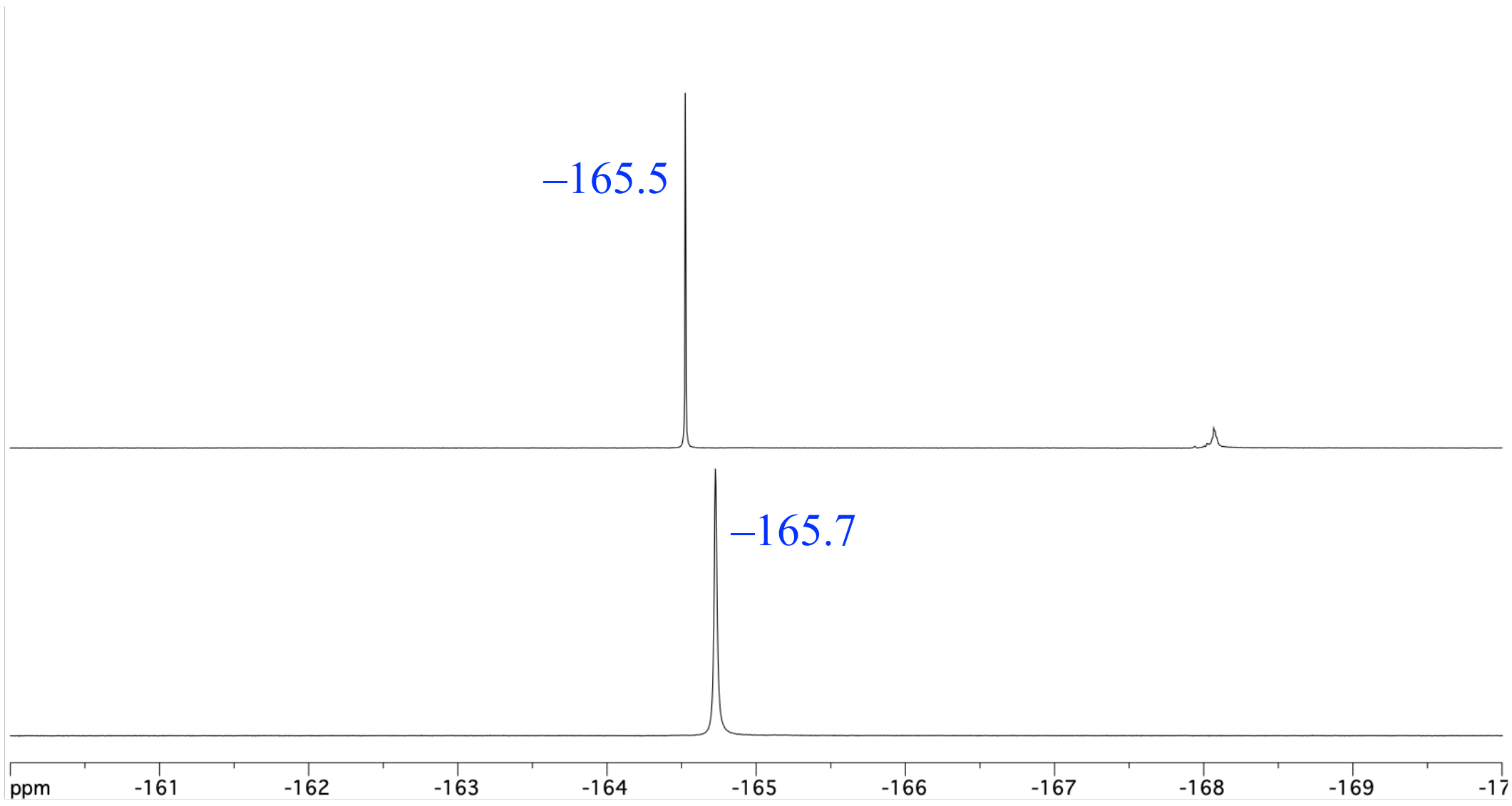

Figure S38: ^{19}F NMR spectra of hexafluorobenzene in 1 vol% CD_3OD in D_2O (top) and of **1** (4 mM) treated with 10 molar equivalents of hexafluorobenzene in the same solvent mixture (bottom) (T = 298 K) (a). The signals of trifluoroacetic acid (δ = –77 ppm), which was used as a reference, are marked with asterisks. In (b), enlarged regions of the spectra with the signals of free (top) and complexed (bottom) hexafluorobenzene are shown.

^{1}H NMR Spectroscopic Binding Studies in Methanol

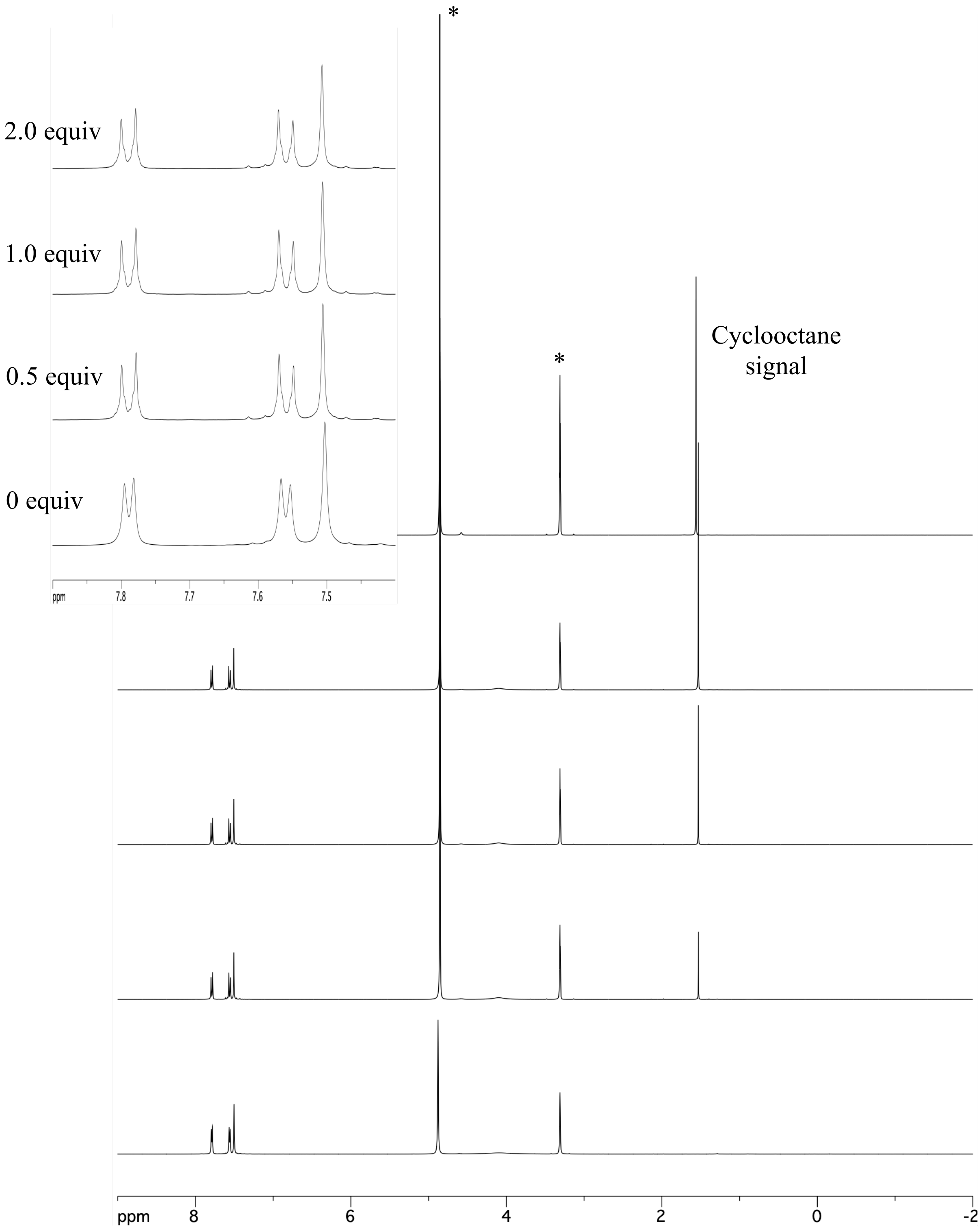

Figure S39: ^{1}H NMR spectra of **1** in CD_3OD (4 mM) in the presence of 0, 0.5, 1.0, and 2.0 molar equivalents of cyclooctane (from bottom). The top spectrum shows the ^{1}H NMR spectrum of cyclooctane in methanol. The solvent and water signals are marked with asterisks. The inset shows an enlargement of the aromatic regions of the spectra.

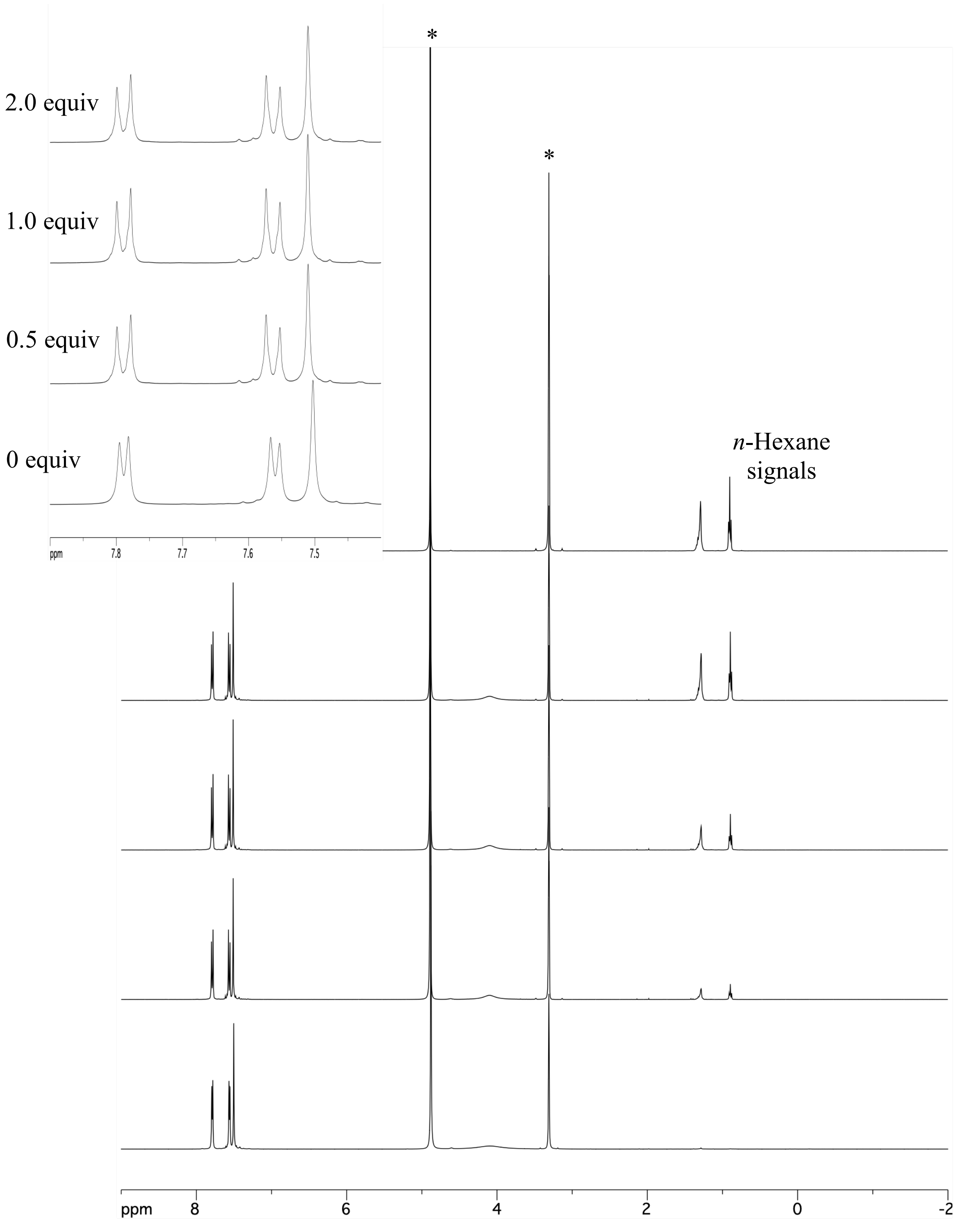

Figure S40: ^{1}H NMR spectra of **1** in CD_3OD (4 mM) in the presence of 0, 0.5, 1.0, and 2.0 molar equivalents of *n*-hexane (from bottom). The top spectrum shows the ^{1}H NMR spectrum of *n*-hexane in methanol. The solvent and water signals are marked with asterisks. The inset shows an enlargement of the aromatic regions of the spectra.

ITC Measurements

The ITC titrations were carried out by using a Microcal VP-ITC. The titrations were performed in H_2O. The calixarene **1** was weighed using an analytical precision balance and dissolved in known volumes of water to achieve a concentration of 0.5 mM. Benzene (89 µL, 1 mmol) was added to water (100 mL) using a Hamilton syringe, resulting in a solution with a 10 mM concentration. Both solutions were loaded into the system for immediate analysis. An ITC experiment involved the titration of the benzene solution into the calixarene solution at 25 °C by using 30 injections of 8 µL at a rate of 0.5 µL s^{-1}, separated by an interval of 180 s. Only in the first injection, a volume of 4 µL was used. Binding constants and enthalpies of binding were obtained by curve fitting of the titration data using the two-site binding model implemented in the SupraFit.[7] The peak produced by the first injection was discarded prior to data processing. The titration was performed in triplicate, and the standard deviations were calculated from the results of the three measurements. The thermograms and binding isotherms of the titrations are shown in Figure S41.

(a)

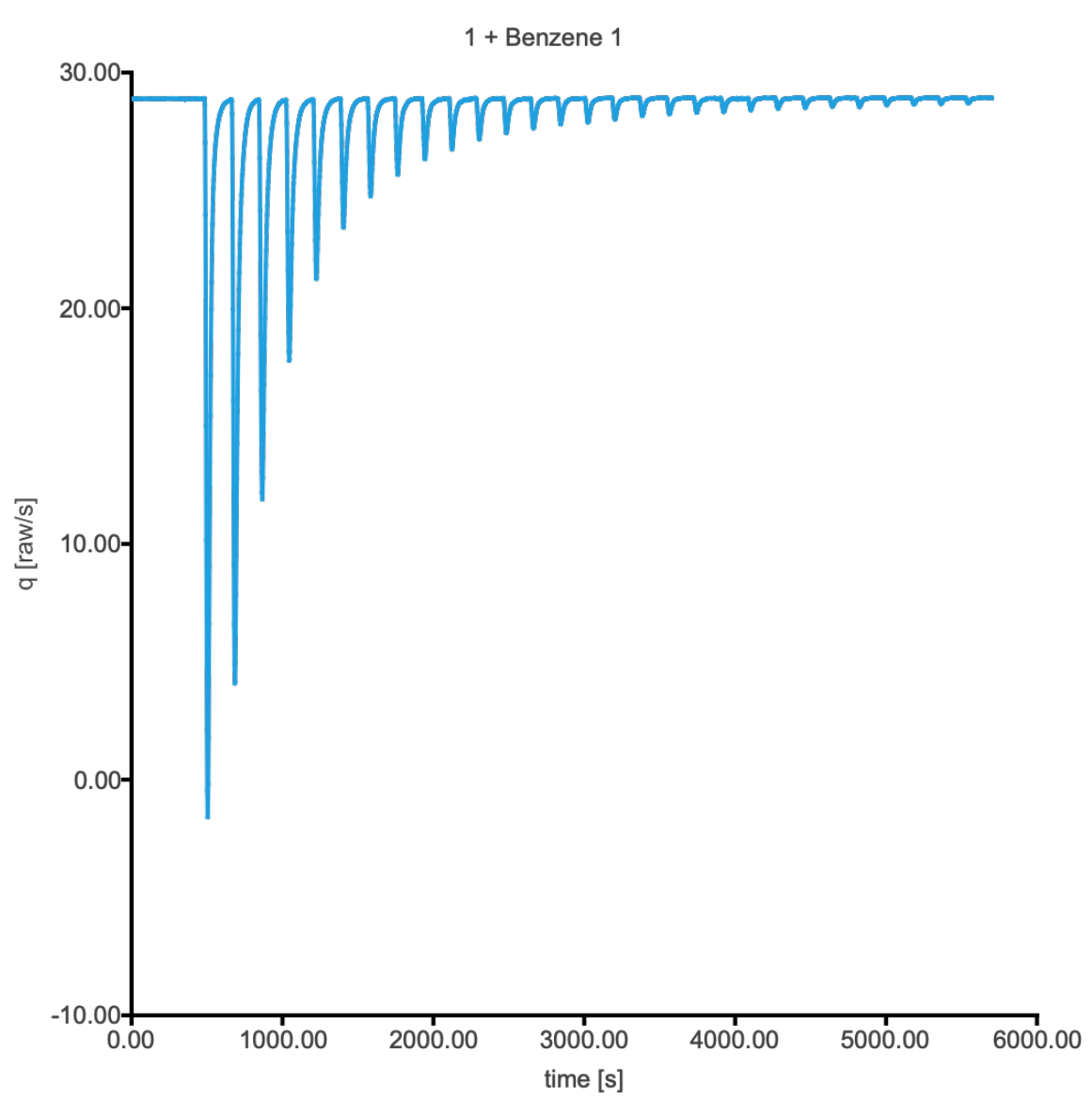

(b)

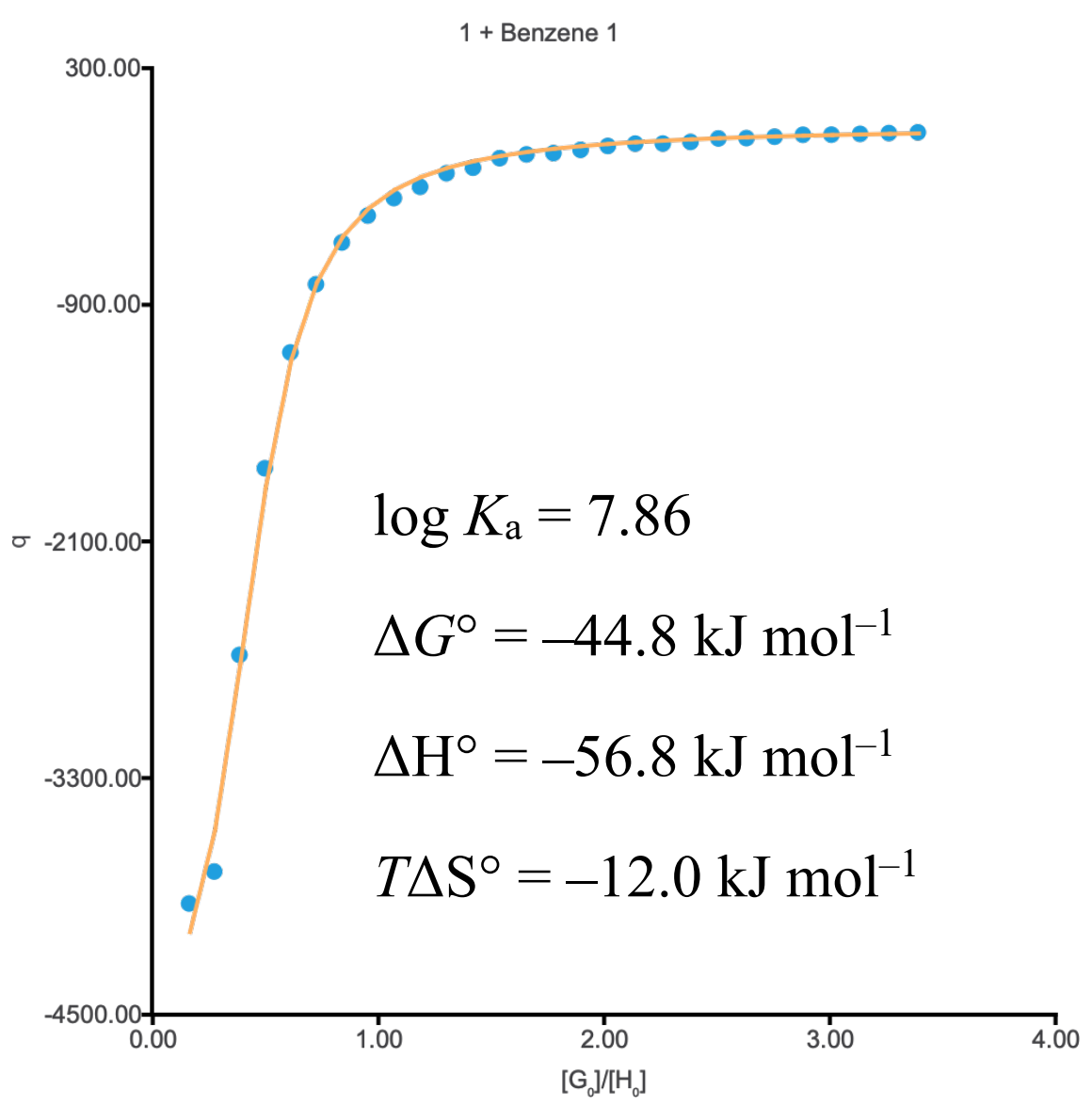

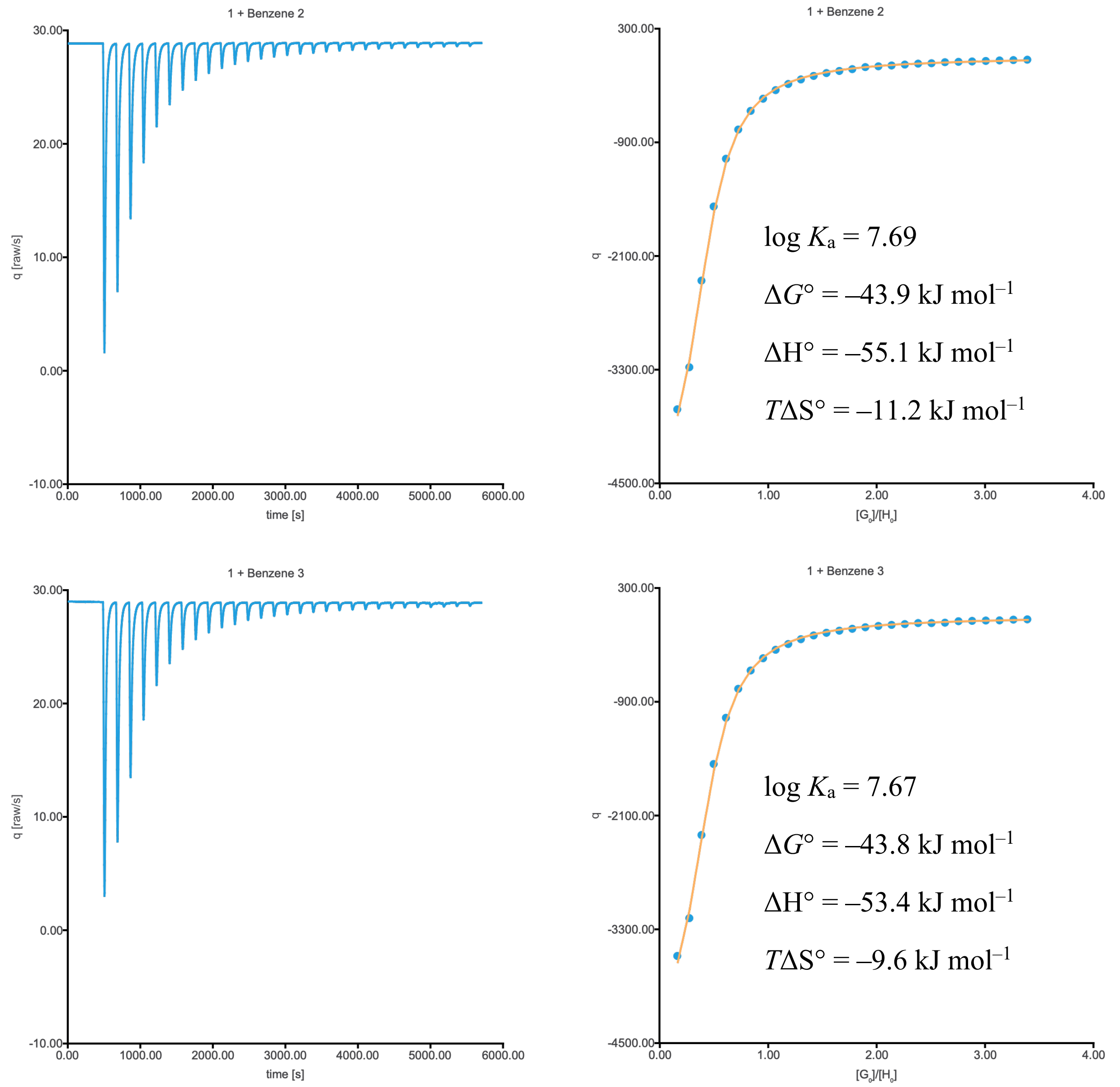

Figure S41: Thermograms (a) and binding isotherms (b) of the ITC titrations of **1** with benzene in H_2O at 298 K.

Details of the Computational Studies

All complexes were optimized using TURBOMOLE[8,9] at the BP86[10,11]-D4[12]/def2-SVP[13] level of theory without symmetry constraints in water using the Conductor-like Screening Model (COSMO) implicit solvation model.[14] Interior cavity volumes and guest volumes were calculated from the optimized structures, and the packing coefficient was obtained as the ratio of guest volume to cavity volume using Swiss-PdbViewer.[15] Because the conformational flexibility of the monomeric calixarene subunits precludes a reliable direct calculation of binding or interaction energies, the relative affinity of the different guests was instead assessed from substitution energies (ΔE_{sub}), calculated for the reaction guest⊂(**1**)$_2$ + *n*-octane → *n*-octane⊂(**1**)$_2$ + guest, using the *n*-octane complex as internal reference. A negative ΔE_{sub} value indicates that substitution of the guest by *n*-octane is thermodynamically favorable (i.e., the *n*-octane complex is more stable); a positive value indicates the opposite.

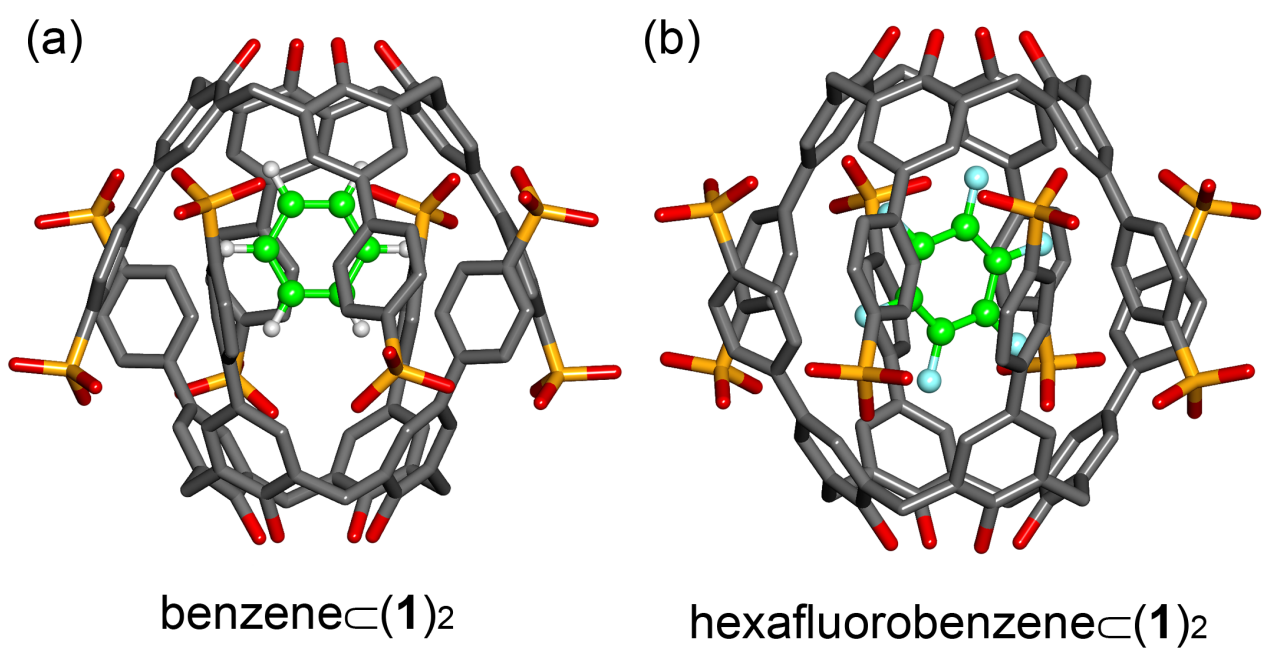

Figure S42: Optimized structures (BP86-D4(COSMO=water)/def2-SVP) of the benzene⊂(**1**)$_2$ complex (a) and the hexafluorobenzene⊂(**1**)$_2$ complex (b). Hydrogen atoms in the two capsule halves are omitted for clarity.

Figure S43 shows the shortest F···π distances evidencing that the C_6F_6 guest avoids π-interactions since all distances are longer than the ideal 3.2 Å, In fact, only one contact is shorter than 3.5 Å.

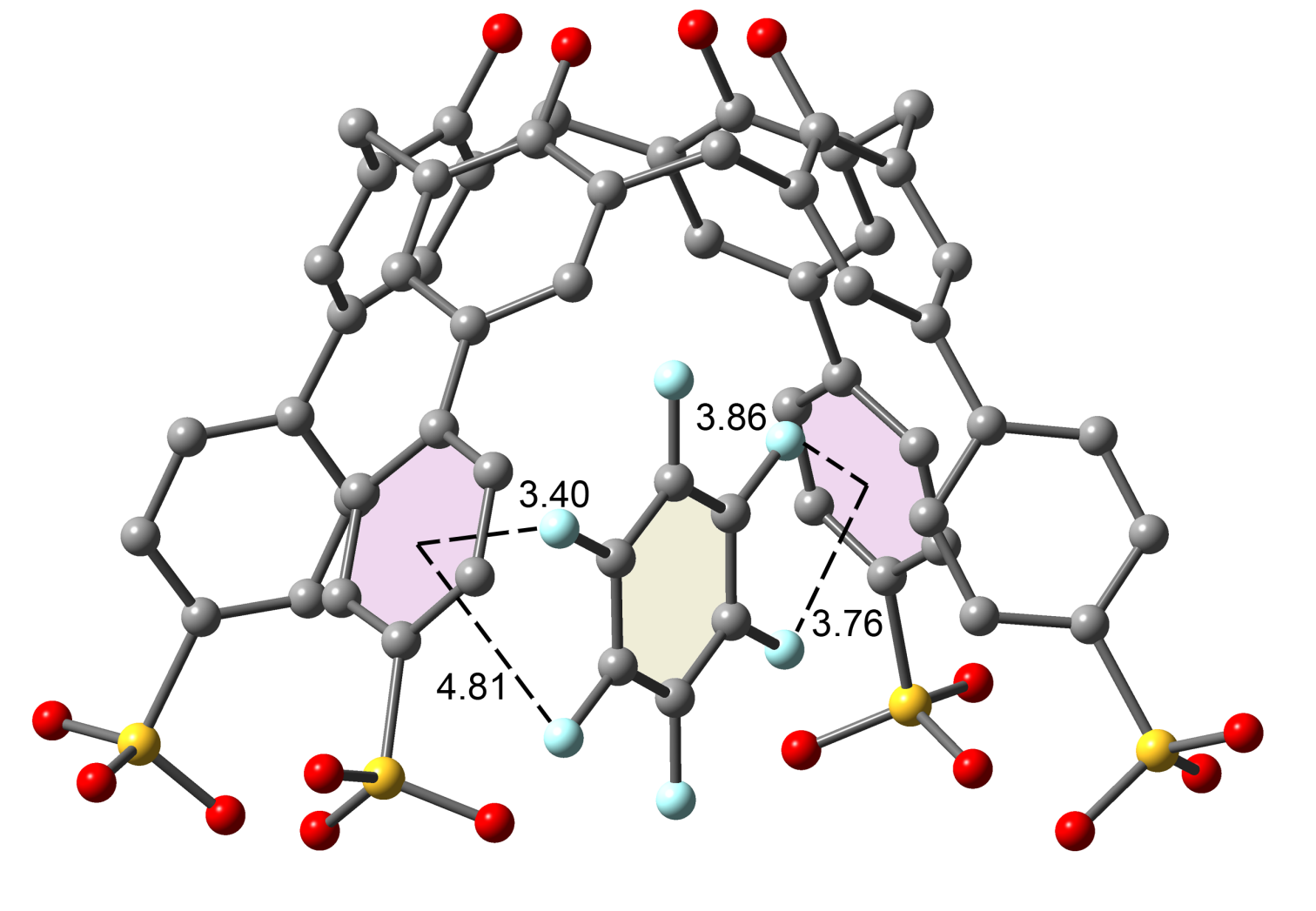

Figure S43: Partial view of the hexafluorobenzene⊂$(\mathbf{1})_2$ complex showing the four shortest F···π distances given in Å

References

[1] H. E. Gottlieb, V. Kotlyar, A. Nudelman "NMR chemical shifts of common laboratory solvents as trace impurities," *J. Org. Chem.* **1997**, *62*, 7512–7515.

[2] B. Mokhtari, K. Pourabdollah "Effect of crown ring size and upper moiety on the extraction of S-block metals by ionizable calixcrown nano-baskets," *Bull. Korean Chem. Soc.* **2011**, *32*, 3979–3990.

[3] T. E. Clark, M. Makha, A. N. Sobolev, H. Rohrs, J. L. Atwood, C. L. Raston "Engineering nanorafts of calixarene polyphosphonates," *Chem. Eur. J.* **2008**, *14*, 3931–3938.

[4] K. Paek, H. Ihm, K. No "Cone-Structured deep cavity formation at upper-rim of calix[4]arene tetrahexyl ether," *Bull. Korean. Chem. Soc.* **1994**, *15*, 422–423.

[5] M. Makha, C. L. Raston "Direct synthesis of calixarenes with extended arms: *p*-phenylcalix[4,5,6,8]arenes and their water-soluble sulfonated derivatives," *Tetrahedron Lett.* **2001**, *42*, 6215–6217.

[6] R. Kaup, A. H. Velders, “Controlling trapping, release, and exchange dynamics of micellar core components,” *ACS Nano* **2022**, *16*, 14611−14621.

[7] C. Hübler, “SupraFit – An open source Qt based fitting application to determine stability constants from titration experiments,” *Chem. Methods* **2022**, *2*, e202200006.

[8] R. Ahlrichs, M. Bär, M. Häser, H. Horn, C. Kölmel, "Electronic structure calculations on workstation computers: the program system TURBOMOLE,” *Chem. Phys. Lett.* **1989**, *162*, 165–169.

[9] S. G. Balasubramani, G. P. Chen, S. Coriani, M. Diedenhofen, M. S. Frank, Y. J. Franzke, F. Furche, R. Grotjahn, M. E. Harding, C. Hättig, A. Hellweg, B. Helmich-Paris, C. Holzer, U. Huniar, M. Kaupp, A. Marefat Khah, S. Karbalaei Khani, T. Müller, F. Mack, B. D. Nguyen, S. M. Parker, E. Perlt, D. Rappoport, K. Reiter, S. Roy, M. Rückert, G. Schmitz, M. Sierka, E. Tapavicza, D. P. Tew, C. van Wüllen, V. K. Voora, F. Weigend, A. Wodyński, J. M. Yu, “TURBOMOLE: modular program suite for *ab initio* quantum-chemical and condensed-matter simulations,” *J. Chem. Phys.* **2020**, *152*, 184107.

[10] A. D. Becke, “Density-functional exchange-energy approximation with correct asymptotic

behavior," *Phys. Rev. A* **1988**, *38*, 3098–3100.

[11] J. P. Perdew, "Density-functional approximation for the correlation energy of the inhomogeneous electron gas," *Phys. Rev. B* **1986**, *33*, 8822–8824.

[12] E. Caldeweyher, S. Ehlert, A. Hansen, H. Neugebauer, S. Spicher, C. Bannwarth, S. Grimme, "A generally applicable atomic-charge dependent London dispersion correction," *J. Chem. Phys.* **2019**, *150*, 154122.

[13] F. Weigend, R. Ahlrichs, "Balanced basis sets of split valence, triple zeta valence and quadruple zeta valence quality for H to Rn: design and assessment of accuracy," *Phys. Chem. Chem. Phys.* **2005**, *7*, 3297–3305.

[14] A. Klamt, G. Schüürmann, "COSMO: a new approach to dielectric screening in solvents with explicit expressions for the screening energy and its gradient," *J. Chem. Soc., Perkin Trans. 2* **1993**, *22*, 799–805.

[15] N. Guex, M. C. Peitsch, "SWISS-MODEL and the Swiss-Pdb Viewer: an environment for comparative protein modeling," *Electrophoresis* **1997**, *18*, 2714–2723.
